# Binding of Nitriles and Isonitriles to V(III) and Mo(III) Complexes: Ligand vs Metal Controlled Mechanism

**DOI:** 10.1021/acs.inorgchem.3c00595

**Published:** 2023-06-28

**Authors:** Taryn D. Palluccio, Meaghan E. Germain, Marco Marazzi, Manuel Temprado, Jared S. Silvia, Peter Müller, Christopher C. Cummins, Jack V. Davis, Leonardo F. Serafim, Burjor Captain, Carl D. Hoff, Elena V. Rybak-Akimova

**Affiliations:** † Department of Chemistry, Tufts University, Medford, Massachusetts 02155, United States; ‡ Departamento de Química Analítica, Química Física e Ingeniería Química, Grupo de Reactividad y Estructura Molecular (RESMOL), Universidad de Alcalá, Alcalá de Henares, Madrid 28805, Spain; § Instituto de Investigación Química ‘Andrés M. del Río’’ (IQAR), Universidad de Alcalá, Alcalá de Henares, Madrid 28805, Spain; ∥ Department of Chemistry, Massachusetts Institute of Technology, Cambridge, Massachusetts 02139, United States; ⊥ Department of Chemistry, University of Miami, Coral Gables, Florida 33146, United States

## Abstract

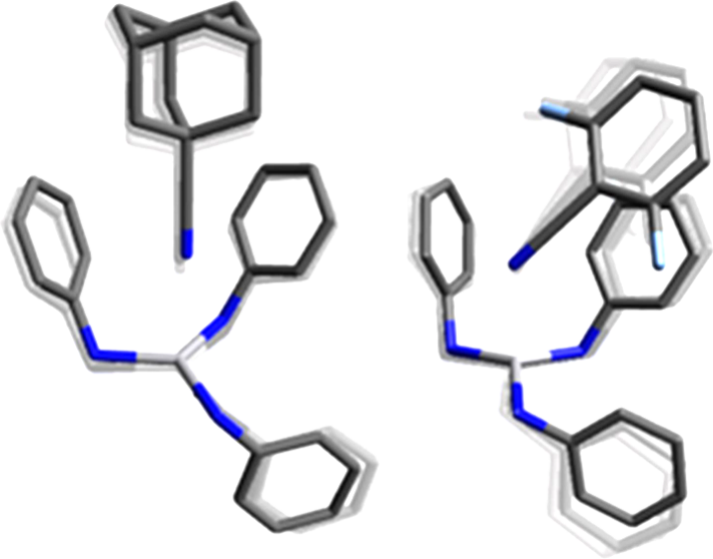

The synthesis and structures of nitrile complexes of V(N[*^t^*Bu]Ar)_3_, **2** (Ar = 3,5-Me_2_C_6_H_3_), are described. Thermochemical and kinetic data for their formation were determined by variable temperature Fourier transform infrared (FTIR), calorimetry, and stopped-flow techniques. The extent of back-bonding from metal to coordinated nitrile indicates that electron donation from the metal to the nitrile plays a less prominent role for **2** than for the related complex Mo(N[*^t^*Bu]Ar)_3_, **1**. Kinetic studies reveal similar rate constants for nitrile binding to **2**, but the activation parameters depend critically on the nature of R in RCN. Activation enthalpies range from 2.9 to 7.2 kcal·mol^–1^, and activation entropies from −9 to −28 cal·mol^–1^·K^–1^ in an opposing manner. Density functional theory (DFT) calculations provide a plausible explanation supporting the formation of a π-stacking interaction between a pendant arene of the metal anilide of **2** and the arene substituent on the incoming nitrile in favorable cases. Data for ligand binding to **1** do not exhibit this range of activation parameters and are clustered in a small area centered at Δ*H*^‡^ = 5.0 kcal·mol^–1^ and Δ*S*^‡^ = −26 cal·mol^–1^·K^–1^. Computational studies are in agreement with the experimental data and indicate a stronger dependence on electronic factors associated with the change in spin state upon ligand binding to **1**.

## Abstract 2

Kinetic and thermodynamic studies of the binding of nitriles to Mo(N[*^t^*Bu]Ar)_3_, **1**, and V(N[*^t^*Bu]Ar)_3_, **2**, are reported. Binding to **1** is dominated by spin crossover from the quartet to the doublet state. Binding to **2** occurs by either a conventional approach of the nitrile toward the metal center or by the establishment of a π interaction between one arene group of the incoming nitrile and a second arene receptor component of one of the anilide ligands of **2**

## Introduction

The study of kinetics and mechanism of ligand binding has a long history in physical inorganic chemistry.^1^ In addition to providing insight to optimize reaction conditions, the pattern of reactivity may provide insight to suggest new approaches in other areas by analogy. It is often found in systems that can be studied that a two-step mechanism occurs: first initial binding and then oxidative addition or other activation reaction. However, sometimes the ligand binding may not be observed due to the fact that unfavorable thermodynamics of binding does not allow the buildup of a detectable intermediate containing the desired ligand bound to the metal center. In other cases, the rate of trapping and conversion of the desired ligand, once it is bound to the metal, may be more rapid than the ligand binding event itself and preclude measurement of the ligand binding step.

There is a long history of comparison of the binding of R–C≡N ligands to N≡N, particularly with respect to end-on coordination of the lone pair of electrons on the sp hybridized N atom present on each.^2^ However, changes in the R group of the nitrile can alter the σ-donor and π-acceptor properties of the ligand and give insight into the nature of the binding site. The steric factors of the R group may also provide information in that regard. In addition, since the N≡N molecule has at its disposal the formation of a range of bridged structures between two or more metals,^3^ the terminal R group in RCN normally limits binding to one metal center in typically an end-on η^1^ or side-on η^2^ architecture.^4^

In a series of reports published by the Cummins group, the exploration of tris-anilide M(N[R]Ar)_3_ (M = Mo, V) complexes has been reported.^5,6^ The two metals Mo and V are the key non-Fe transition metal components of naturally occurring nitrogenase enzymes.^7^ Due to the complex nature of the enzymatic process, there does not at this time appear to be a full mechanistic understanding of how the binding of dinitrogen occurs.

Mo(N[*^t^*Bu]Ar)_3_, **1** (Ar = 3,5-Me_2_C_6_H_3_), reacts with dinitrogen at room temperature and below, leading to the formation of the N_2_-bridged dinuclear complex, {Mo(N[*^t^*Bu]Ar)_3_}_2_(μ-N_2_), which reacts further to form two equivalents of the metal nitride, N≡Mo(N[*^t^*Bu]Ar)_3_.^8^ Crystal structures have been determined for the isolable bridging dimer and the terminal nitride formed when the coordinated N_2_ ligand is split.^8c^ However, in spite of over 25 years of work, the mode of binding of N_2_ to complex **1** is not known.

**Scheme 1:**
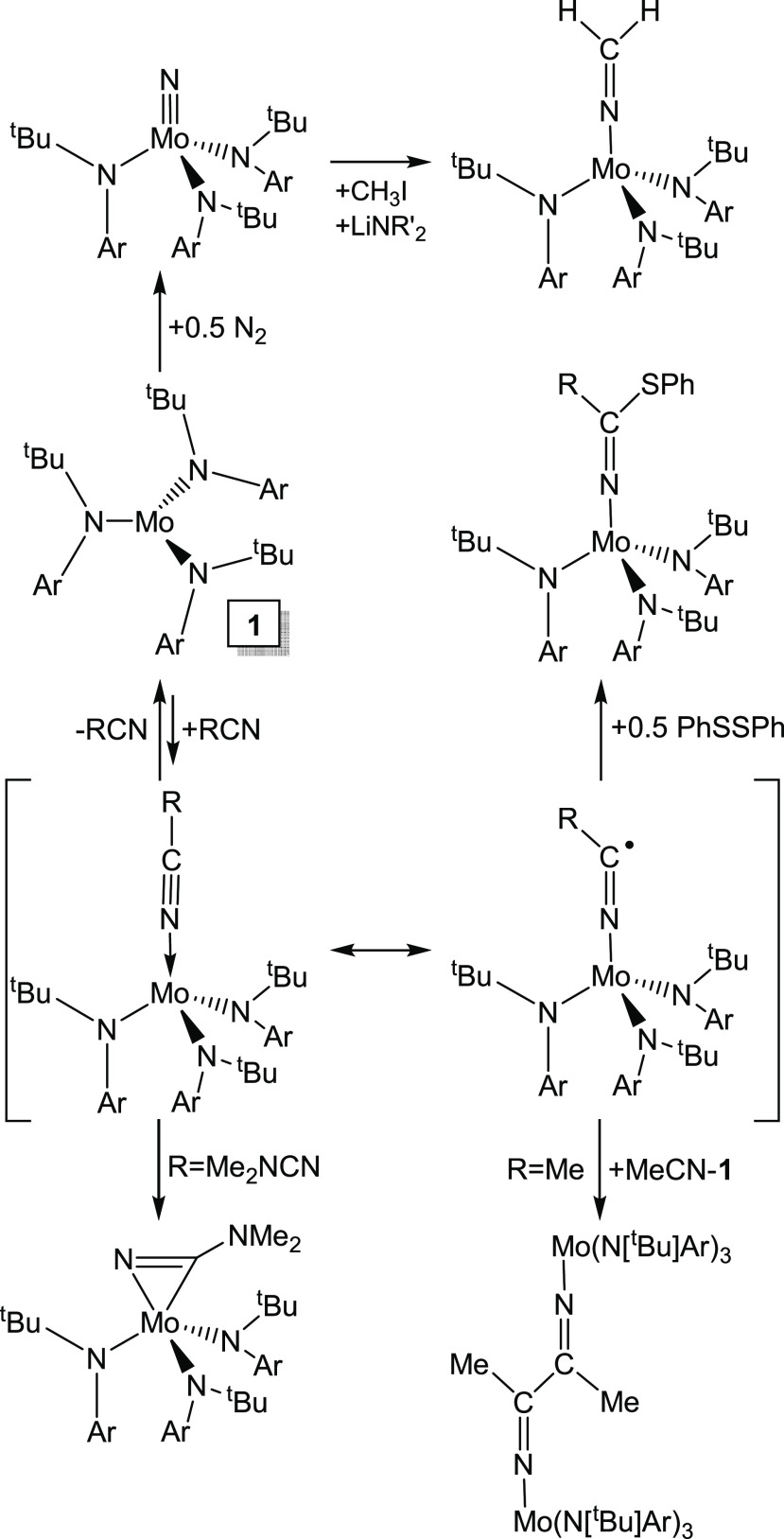
Summary of Reactions of Complex **1** with Dinitrogen and Nitriles Ar = 3,5-Me_2_C_6_H_3_; R′ = SiMe_3_. Adapted from Ref (11). Copyright 2006 American Chemical Society

Moreover, we have reported thermodynamics and kinetics of ligand binding to complex **1** using various nitriles that afford end-on (η^1^) or side-on (η^2^) adducts (see Scheme 1).^9^ For example, benzonitrile binds rapidly and reversibly to **1**; spectroscopic and computational data indicate an η^1^ coordination of benzonitrile and related aryl nitriles. In contrast, Me_2_NCN initially binds end-on but then isomerizes rapidly to an η^2^ side-on adduct that is stable and relatively unreactive.^2b,9^ At low temperatures (from −80 to −40 °C), the η^1^ intermediate Me_2_NCNMo(N[*^t^*Bu]Ar)_3_ complex can be trapped via radical coupling with PhSSPh, giving the ketimide complex Me_2_NC(SPh)NMo(N[*^t^*Bu]Ar)_3_. At higher temperatures, the η^1^- to η^2^-isomerization is faster than the reaction with the disulfide. The production of ketimide complexes starting from either N_2_ and **1** or from RCN and **1** was studied, as shown in Scheme 1. Further work showed that by a combination of reagent additions, the molybdenum nitride could be converted to a nitrile, closing the loop on a potential catalytic conversion of dinitrogen to nitriles via complex **1**.^10^

While the high propensity of η^1^-aryl nitrile adducts with Mo(III) anilides to undergo radical couplings at the nitrile carbon atom led to limited stability of these species and precluded their structural characterization,^9^ recently, Power and co-workers have reported the synthesis, isolation, and molecular structures of several V{N[SiMe_3_]_2_}_3_(RCN)_2_ and V{N[SiMe_3_]_2_}_3_(RNC)_2_ complexes by reaction of the related V(III) complex, V{N[SiMe_3_]_2_}_3_, and several nitriles or isonitriles.^12^

The binding of nitriles is not of interest only as a model for end-on η^1^ binding of N_2_, but there is also an emerging chemistry of nitrile ligands and their transformation.^4,13^ The same is true for isonitrile RN≡C ligands, whose simple binding chemistry is analogous to O≡C.^14^ Attempts to convert either catalytically or stoichiometrically metal nitrides to other derivatives have been recently reviewed.^15^ The catalytic chemistry of both nitrile and isonitrile complexes has also received a revival in interest.^14,16^

In this paper, we are currently interested in nitrile binding to V(N[*^t^*Bu]Ar)_3_, **2** (Ar = 3,5-Me_2_C_6_H_3_),^6^ for comparison to the related complex **1**. Therefore, we describe the synthesis and molecular structure of stable η^1^-nitrile complexes of **2** and the kinetic and thermodynamic parameters for their formation. A consistent picture evolves, and reasonable accord is achieved based on stopped-flow kinetic, synthetic, structural, variable temperature infrared, solution calorimetric, and density functional theory (DFT) computational studies. A range of nitriles as well as an isonitrile ligand were employed in this work in order to gain insight into the similarities and differences in the electronic and steric factors that govern ligand binding to the sterically shielded, coordinatively unsaturated vanadium(III) and molybdenum(III) complexes **1** and **2**.

## Results

### Preparation of η^1^-RCN-V(N[*^t^*Bu]Ar)_3_ Complexes

Addition of nitriles to dark green-brown solutions of **2** in diethyl ether or toluene results in an immediate color change to deep purple. The adduct with benzonitrile, PhCN-V(N[*^t^*Bu]Ar)_3_ (PhCN-**2**), can be isolated as purple crystals by recrystallization from *n*-hexane at −35 °C, but repeated attempts to obtain crystals suitable for X-ray diffraction studies were met without success. The reaction of **2** and 2,6-F_2_C_6_H_3_CN (DFBN) also resulted in the rapid formation of a purple color but, in this case, single crystals of DFBN**-2** could be grown from concentrated diethyl ether solutions. The reaction of **2** with Me_2_NCN in diethyl ether results in the formation of a deep blue/purple solution from which the product Me_2_NCN-**2** can be isolated as a cerulean blue solid by precipitation from *n*-pentane. Crystals of Me_2_NCN-**2** suitable for X-ray diffraction studies could be grown from concentrated solutions in a 1:1 mixture of toluene/diethyl ether at −35 °C. The ^1^H NMR spectra of isolated and purified nitrile complexes were collected in C_6_D_6_. All complexes gave rise to paramagnetically shifted and broadened resonances. No evidence was obtained for the formation of an isomeric η^2^-complex of Me_2_NCN-**2** even in low-temperature NMR studies at −80 °C in toluene-*d*_8_.

**Figure 1:**
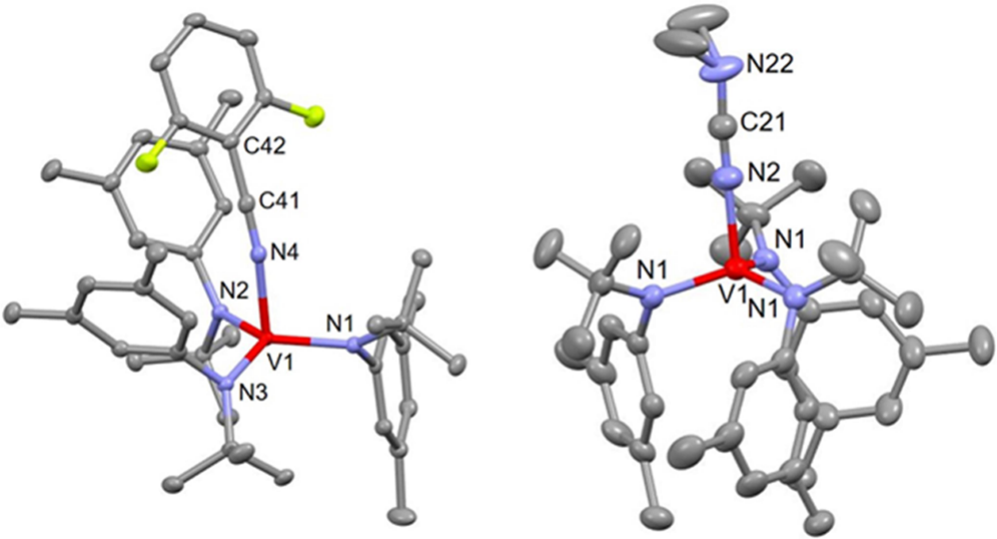
Solid-state structure of DFBN**-2** (left) and Me_2_NCN-**2** (right) with thermal ellipsoids at 50% probability. Hydrogen atoms have been omitted for clarity. Selected distances (Å) and angles (degree). For DFBN-**2**: V1–N1 = 1.9439(15), V1–N2 = 1.9412(16), V1–N3 = 1.9416(16), V1–N4 = 2.0417(16), N4–C41 = 1.151(2), N1–V1–N4 = 99.89(6), N2–V1–N4 = 89.68(6), N3–V1–N4 = 98.71(6), V1–N4–C41 = 161.79(15); for Me_2_NCN-**2**: V1–N1 = 1.9351(15), V1–N2 = 2.038(3), N2–C21 = 1.149(4), N1–V1–N1 = 116.49(3), N1–V1–N2 = 100.94(5), V1–N2–C21 = 180.0(4).

The solid-state structure of DFBN**-2** (Figure 1) contains a vanadium metal center in a distorted trigonal pyramidal coordination geometry ((N_anilide_–V–N_nitrile_)_avg_ = 96.1(1)°, (N_anilide_–V–N_anilide_)_avg_ = 118.9(1)°). The V–N_nitrile_ interatomic distance (2.042(2) Å) is longer than the V–N_anilide_ distances (1.942(2) Å avg). The anilide ligands adopt a one-down, two-up arrangement, where one of the three aryl rings points downward away from the nitrile and two point upward toward it. This conformation likely arises from steric effects between the coordinated nitrile and the *tert*-butyl groups of the amide ligands. Furthermore, there is a deviation from linearity in the angle V–N–C (161.8(2)°), which is attributed to a π-stacking interaction between the 2,6-F_2_C_6_H_3_ aryl group on the nitrile and the arene group of one of the anilide ligands bound to vanadium, a behavior also previously noticed by us in the complex Ph(H)CN-Mo(N[*^t^*Bu]Ar)_3_.^17^

The solid-state structure of Me_2_NCN-**2** determined by X-ray crystallography (Figure 1) reveals an η^1^ binding mode for the cyanamide ligand; in contrast, the adduct of Me_2_NCN with **1** has been shown in previous work to yield a stable η^2^-bound derivative.^9^ The anilide ligands adopt a crystallographically imposed three-fold symmetric arrangement, which is markedly different from that observed in the structure of DFBN**-2**. The V–N_nitrile_ interatomic distance of 2.038(3) Å is comparable with the V–N_nitrile_ distance of 2.042(2) Å observed in DFBN**-2**. The reported data are comparable to those obtained for related V(III) structures in the literature.^12,18^

### Oxidation of RCN-2 Complexes

**Figure 2:**
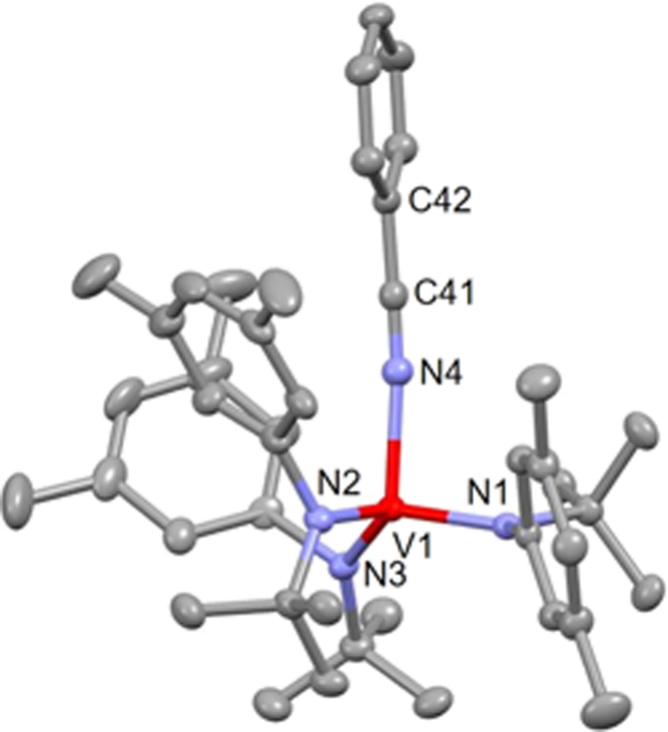
Solid-state structure of [PhCN-**2**][BAr_4_^F^] with thermal ellipsoids at 50% probability. Hydrogen atoms, [BAr_4_^F^]^−^, and interstitial diethyl ether are omitted for clarity. Selected bond lengths (Å) and angles (degree): V1–N4 = 2.0598(15), N4–C41 = 1.145(2), V1–N4–C41 = 172.84(15).

We have previously observed that the addition of *^t^*BuCN to **2** followed by the reaction with O_2_ results in the oxidation of the bound nitrile to form the acylimido species *^t^*BuC(=O)N–V(N[*^t^*Bu]Ar)_3_.^19^ However, the neutral RCN-**2** complexes prepared as described in a previous section were found to react with outer-sphere oxidants to give the corresponding cationic nitrile complexes [RCN-**2**]^+^. A solution of PhCN-**2** was added to a stirring suspension of [(C_5_H_5_)_2_Fe][B(3,5-(CF_3_)_2_C_6_H_3_)_4_] (FcBAr_4_^F^), both in diethyl ether, whereupon the color of the solution changed to dark green. The product [PhCN-**2**][BAr_4_^F^] could be crystallized from the reaction mixture by storing the solution at −35 °C for 1 day. The solid-state structure of the complex was determined using single-crystal X-ray diffraction methods and revealed a four-coordinate vanadium center with an η^1^-nitrile ligand (Figure 2).

The vanadium–nitrogen distance in [PhCN-**2**][BAr_4_^F^] is only slightly longer than that of either complex DFBN**-2** or Me_2_NCN-**2**. As in DFBN**-2**, two of the aryl moieties are positioned in proximity to the nitrile ligand. The arene of PhCN in the structure of the complex cation [PhCN-**2**]^+^ is positioned in a relatively symmetrical manner between the two anilide aryl groups with no real sign of arene–arene interaction compared to the structure of the DFBN**-2** complex in Figure 1. In addition, the C–N–V angle of 173° in [PhCN-**2**][BAr_4_^F^] is noticeably closer to linear than in DFBN**-2** (162°). The third anilide ligand is rotated ∼90° with respect to the other anilide ligands.^20^

### Qualitative Fourier Transform Infrared (FTIR) and Thermochemical Studies of Nitrile Binding to **2**

Aryl nitrile coordination to **2**, in addition to the color change mentioned above, could also be detected by FTIR. The intensity of the ν_CN_ band and its location were sensitive to the RCN substituents. Spectroscopic data for nitrile binding are summarized in Table S1 of the Supporting Information. Surprisingly, simple alkyl nitriles such as MeCN and AdCN did not produce a detectable IR peak identifiable as ν_CN_, even at low temperatures. The addition of solid **2** to a stock solution of AdCN resulted in a decrease in ν_CN_ assigned to the free nitrile ligand with no new band occurring in that region. This is attributed to the bonding being primarily sigma donation from N to V, resulting in a very small oscillating dipole in RCN-**2**. It is worth noting that others have reported greatly reduced extinction coefficients for some nitrile complexes.^21^

Equilibrium constants for reversible binding of DFBN to **2** were measured in toluene solution by variable temperature FTIR spectroscopy in the range *T* = 9–49 °C. Representative data are shown in Figure S1 of the Supporting Information. Due to the highly air-sensitive nature of these solutions, three determinations of *K*_eq_ as a function of *T* were made (see Table S2 of the Supporting Information). A van’t Hoff plot is shown in Figure S2 of the Supporting Information from which we derive Δ*H* = −10.4 ± 0.8 kcal·mol^–1^ and Δ*S* = −26 ± 4 cal·mol^–1^·K^–1^ for binding of DFBN to **2** in toluene solution.

Moreover, we have previously reported the enthalpy of binding of AdNC to **2** as −17.1 ± 0.7 kcal·mol^–1^.^22^ In a series of experiments, including Me_2_NCN, DFBN, C_6_F_5_CN, and 4-F_3_C-C_6_H_4_CN, signs of equilibrium between AdNC and the corresponding nitrile adducts were sought as shown in eq 1.
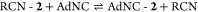
1In all cases, starting from either side of the proposed equilibrium, only AdNC-**2** and the corresponding nitrile were observed. The error limit for the detection of binding was estimated to be on the order of 1–3%, implying that the *K*_eq_ for binding of AdNC was at least 100 times larger than the corresponding *K*_eq_ for binding of the nitriles. This observation implies that Δ*G*° for binding the nitriles studied is at least 2 kcal·mol^–1^ less favorable than for binding AdNC.

A number of attempts to measure the enthalpies of binding of RCN to **2** by reaction calorimetry were made at 30 °C in toluene-*d*_8_ using a large excess of **2** and a limiting amount of RCN. However, neither IR nor NMR data allowed quantitative determination of the products since a large excess of nitrile was needed to ensure quantitative binding. Data for binding of AdCN, Me_2_NCN, and PhCN, in the broad range of Δ*H* = −14.5 ± 2 kcal·mol^–1^ were obtained as described in the Supporting Information. These data were in approximate agreement with enthalpies of replacement of formed nitrile complexes by AdNC.

### Stopped-Flow Kinetic Studies of Nitrile Binding to **2**

**Figure 3:**
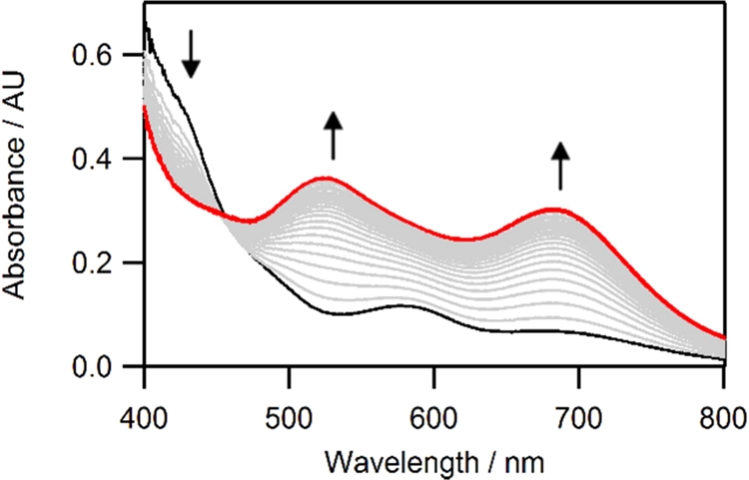
Time-resolved spectra of DFBN (1 mM) binding to **2** (0.3 mM) in toluene solution at −44 °C, acquired over 2 s. Selected traces are shown for clarity. The initially recorded spectrum is shown in black, and the final spectrum is shown in red.

The rapid binding kinetics of several nitriles (aromatic nitriles: DFBN, PhCN, and MesCN, and aliphatic nitriles: Me_2_NCN, AdCN, and MeCN) to **2** were investigated using stopped-flow methodology with spectrophotometric registration. The growth of visible absorption bands was observed in all reactions, and nitrile binding was very clean and well-behaved. For example, the time-resolved spectra for DFBN binding (Figure 3) reveals significant buildup at λ = 525 nm and λ = 687 nm. Similar spectral changes were observed for other nitriles (Figures S3–S7 of the Supporting Information).

**Figure 4:**
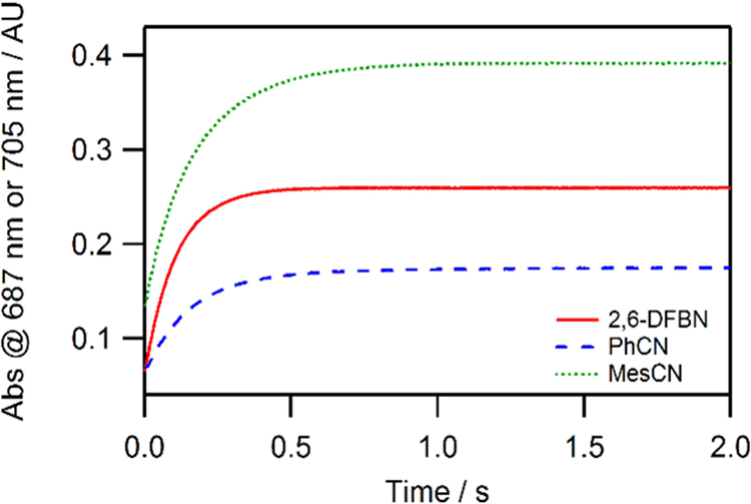
Single-wavelength kinetic traces of aromatic RCN (1 mM) binding to **2** (0.3 mM) in toluene solution at −44 °C (DFBN and PhCN at λ = 687 nm; MesCN at λ = 705 nm).

Single-wavelength measurements were necessary to quantify rapid nitrile binding to **2** at variable concentrations and temperatures (typically, −62 to −35 °C). Representative kinetic traces recorded for aromatic nitrile binding in single-wavelength mode are shown in Figure 4.

**Figure 5:**
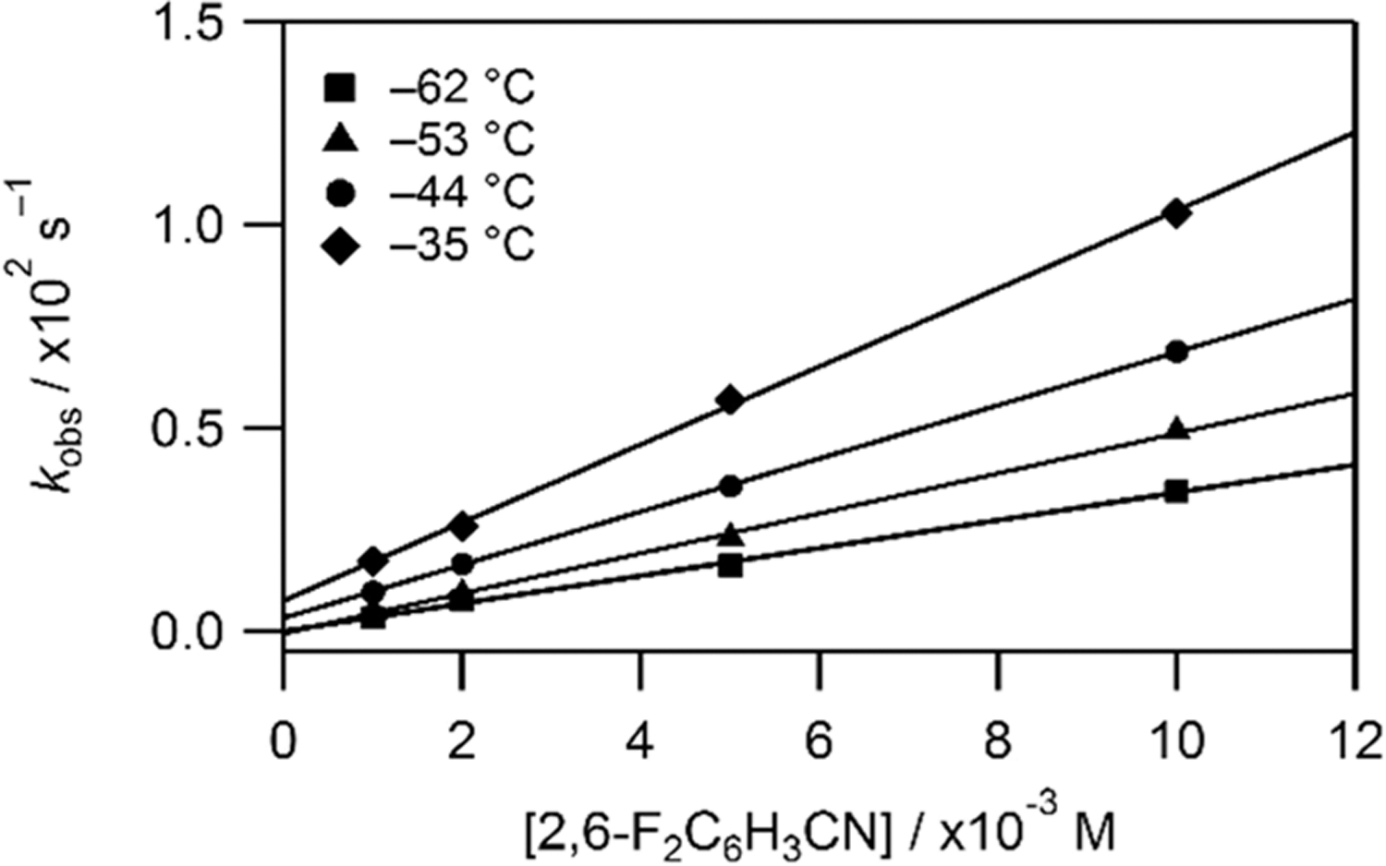
Second-order rate plot for DFBN binding to **2** at various concentrations (1–10 mM) over a temperature range of −62 to −35 °C with [**2**]_0_ = 0.3 mM.

Varying the concentration of the nitriles resulted in a linear increase in *k*_obs_ (Figure 5).

This observed behavior corresponds to the reaction of reversible nitrile binding to **2** as described by eq 2.
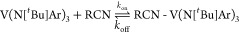
2The dependence of *k*_obs_ = *k*_on_[RCN] + *k*_off_ shown in Figure 5 reveals a linear relationship with the slope equal to *k*_on_ and an intercept corresponding to *k*_off_. At lower temperatures, essentially zero intercepts indicated that the equilibrium was completely shifted toward product formation. At higher temperatures, such as −35 °C for DFBN, as shown in Figure 5, the reverse reaction, nitrile dissociation, became noticeable, as indicated by an increasing intercept corresponding to *k*_off_. The equilibrium nature of nitrile binding at higher temperatures is in keeping with the FTIR studies described above. The accuracy in *k*_off_ values (and the derived values of *K*_eq_) is relatively low, as these values are determined from the intercepts of the dependencies of *k*_obs_ on the concentrations of nitrile as shown in Figure 5. Kinetic estimates of *K*_eq_ were not warranted. The slope of the graph indicative of *k*_on_ is used for kinetic analysis of nitrile binding. Rate constant data at *T* = −40 °C are summarized in Table 1 (additional data at other temperatures are available in Supporting Information Tables S3–S8). Activation parameters for nitrile binding were calculated from Eyring plots (Figures S10–S15 of the Supporting Information), and the determined values are also collected in Table 1.

**Table 1:** Bimolecular Rate Constants (*k*_on_) and Activation Parameters Measured for Coordination of Various Nitriles to **2**^a^

RCN	*k*_on_ (−40 °C)^b^ (M^–1^·s^–1^)	Δ*H*^‡^ (kcal·mol^–1^)	Δ*S*^‡^ (cal·mol^–1^·K^–1^)	Δ*G*^‡^ (−40 °C) (kcal·mol^–1^)
PhCN	(8.1 ± 0.2) × 10^3^[468 ± 22]	2.9 ± 0.6[5.2 ± 0.2]	–28 ± 3[−24 ± 1]	9.4[10.8]
DFBN	(7.5 ± 0.2) × 10^3^[316 ± 3]^c^	3.3 ± 0.2[4.7 ± 0.4]^c^	–26 ± 1[−26 ± 2]^c^	9.4[10.8]^c^
MesCN	(6.6 ± 0.3) × 10^3^[193 ± 14]	6.4 ± 0.3[5.0 ± 0.3]	–13 ± 2[−26 ± 1]	9.4[11.1]
Me_2_NCN	(15.5 ± 0.7) × 10^3^[708]^d^	5.6 ± 0.3[6.4 ± 0.4]^d^	–15 ± 1[−18 ± 2]^d^	9.0[10.6]^d^
MeCN	(10.1 ± 0.5) × 10^3^	7.2 ± 0.3	–9 ± 2	9.2
AdCN	(5.1 ± 0.5) × 10^3^[97]	6.7 ± 0.8[5 ± 1]	–12 ± 4[−28 ± 5]	9.6[11.5]
AdNC	(10.1 ± 0.9) × 10^3^[16 × 10^3^]	4.6 ± 0.3[5.5 ± 0.5]	–20 ± 1[−15 ± 4]	9.2[9.0]

a For comparison purposes, data reported^2b,23^ for complex **1** between brackets.

b Rate constants at −40 °C were extra- or interpolated from Eyring plots.

c Determined in this work.

d *k*_on_ and activation parameters represent the formation of the end-on (η^1^) adduct.

For direct comparison of electron-deficient aromatic nitrile binding to vanadium and molybdenum *tris*-anilide complexes, the kinetics of DFBN coordination to **1** was also studied (see the Supporting Information, Figures S9, S17, and Table S10) and revealed spectral changes and reaction rates very similar to the overall behavior of other aromatic nitriles (PhCN and MesCN) reported previously.^2b^ Derived kinetic parameters are also compiled in Table 1.

In order to compare nitrile binding and isonitrile binding to **2**, stopped-flow kinetic experiments were also performed with AdNC. Time-resolved spectral changes and overall kinetics were remarkably similar to those observed for aliphatic nitrile binding (see Supporting Information, Figures S8, S16, and Table S9), revealing a rapid second-order process, which is somewhat faster for AdNC than for AdCN. In contrast to nitrile binding, coordination of AdNC to **2** proved to be essentially irreversible over a broad temperature range. The values of the rate constant at *T* = −40 °C and the activation parameters are also summarized in Table 1. The reaction of **1** with AdNC was studied previously.^23^

Surprisingly, the values of Δ*G*^‡^ at *T* = −40 °C for binding to **2** are approximately constant at 9.3 ± 0.3 kcal·mol^–1^ as are those for **1** at 11.0 ± 0.5 kcal·mol^–1^ with the exception of binding of AdNC to **1**, which exhibits a lower value of 9.0 kcal·mol^–1^. This is the only ligand studied for which the rate of binding to **1** is faster than to **2**. This is primarily associated with a less unfavorable entropy of activation.

### DFT-Optimized Structures of **2**

**Figure 6:**
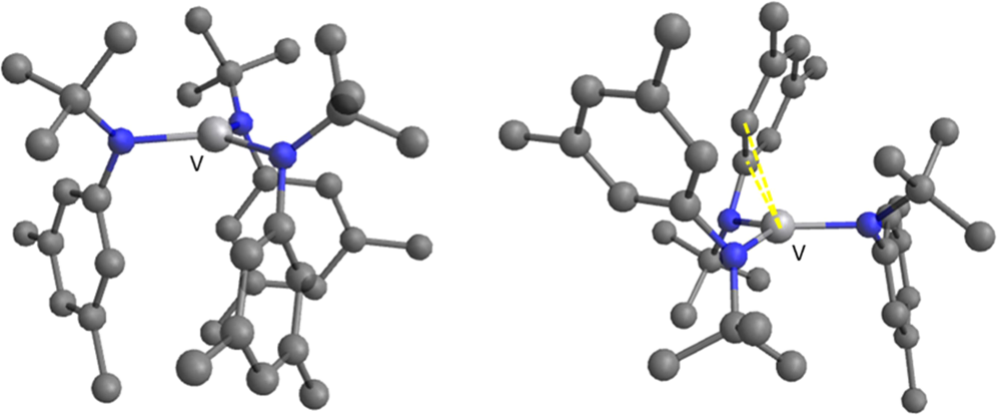
Optimized structures at the PBE0-D3(BJ)/Def2-SV(P) level of theory^24−26^ of the two most stable configurations (in terms of Δ*G* (25 °C)) of **2** with the anilide ligands adopting a three-down (configuration **A**, left) or a one-down, two-up arrangement (configuration **B**, right). Hydrogen atoms are omitted for clarity.

Since the structure of **2** is not known, DFT calculations were performed at the PBE0-D3(BJ)/Def2-TZVP, IEFPCM(toluene)//PBE0-D3(BJ)/Def2-SV(P) level of theory^24−27^ (see the Supporting Information for full computational details). The DFT-optimized structures of the two most thermodynamically stable conformations computed for **2** are shown in Figure 6.

Configuration **A** is a “3 anilides-down” configuration analogous to that adopted by **1**.^8^ Configuration **B** is a “2 anilides up-1 down” and contains a stabilizing interaction between V and an arene group pendant on one of the anilide ligands analogous to that previously noticed in the solid-state structure of the related compound V(N[Ad]Ar)_3_ (Ad = adamantyl, Ar = 3,5-Me_2_C_6_H_3_).^28^ We previously reported a computed difference of ≈−4 kcal·mol^–1^ in enthalpy between both structures favoring configuration **B**;^29^ however, that difference is lowered to −0.6 kcal·mol^–1^ when London dispersion interactions are taken into account in the calculations^30^ by the use of Grimme’s popular D3 correction.^31^ The difference in computed Δ*S* values is small but favors **A** over **B**, and, at 25 °C, the computed Gibbs energy differences are negligible. Moreover, the interconversion process between both conformations has been computed to have a small barrier on the order of 7 kcal·mol^–1^, as can be seen in Figure S23 in the Supporting Information. Thus, computational data predict the establishment of a fast equilibrium between both configurations in the gas phase at room temperature and below. Moreover, as shown in Figures S20–S22 in the Supporting Information, several additional optimized structures of **2** were computed, all within a range of 3 kcal·mol^–1^.

**Figure 7:**
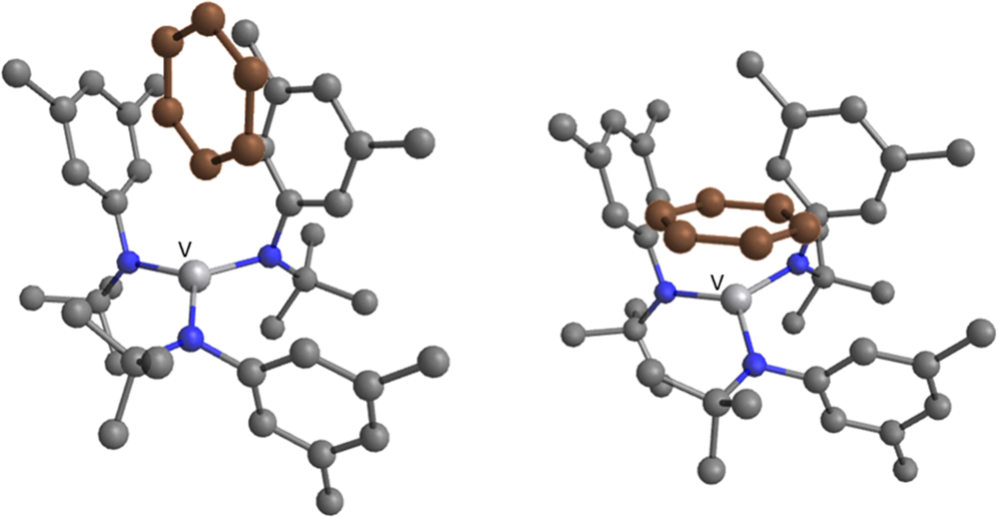
DFT-Optimized structures of **2** interacting with a benzene molecule (in orange-brown color) at the PBE0-D3(BJ)/Def2-SV(P) level of theory.^24−26^ Hydrogen atoms are omitted for clarity.

Furthermore, the crystal structure of complex DFBN-**2** in Figure 1 prompted computational investigation of a plausible stabilization of **2** by interaction with the solvent. Two different structures were optimized using the **B** configuration of **2**, including explicitly a benzene molecule to simulate the toluene solvent, and are shown in Figure 7.

The structure on the left contains a π-stacking stabilizing interaction analogous to that observed in the crystal structure of DFBN-**2** shown in Figure 1, and the alternative structure on the right exhibits two C–H···π interactions between benzene and the two arenes located in the same side of the plane defined by the three anilide nitrogens. In both cases, the interaction of the benzene molecule with the V center is not significant since the shorter distances between the two moieties are V···H = 2.92, V···C = 3.83 Å (Figure 7 left) and V···H = 3.45, V···C = 3.94 Å (Figure 7 right). These structures are stable with respect to the enthalpy of reaction by −1 kcal·mol^–1^ (Figure 7 left) and −2.2 kcal·mol^–1^ (Figure 7 right) related to the **B** configuration of **2**, shown in Figure 6. The entropy change in conversion of the intercalated structure on the left to the structure on the right was computed and found to be −0.6 cal·mol^–1^·K^–1^, indicating that the latter structure would be predicted to be slightly more stable with respect to Gibbs energy at 25 °C.

### DFT-Computed Structural and Spectroscopic Properties of Complexes of **1** and **2**

Structural and spectroscopic data were computed for complexes of **1** and **2**. Full data are available in Tables S18–S21 in the Supporting Information, and when the experimental data were available, generally good agreement with the computed parameters was observed. In spite of the larger ionic radius for Mo(III) compared to V(III), the computed M–L bond lengths were found to be shorter for the η^1^-complexes of **1** when compared to the analogous compounds of **2**. In addition, the N≡C bonds in the coordinated nitriles and isonitriles were also computed to be longer for **1** than for **2**, suggesting more effective back-bonding for Mo compared to V. This is also confirmed in computed and experimental infrared spectroscopic data (see Tables S1 and S21 in the Supporting Information). The N–C–R moieties in V–nitrile compounds are essentially linear, and M–N–C fragments bend in the case of the arene nitriles to maximize the π-stacking interactions analogous to that previously described for the solid-state structure of DFBN-**2** shown in Figure 1. For Mo, there is a stronger deviation from linearity in the N–C–R angle of nitriles, and in particular, the C–N–R linkage in isonitriles is notably bent for Mo but nearly linear for V-bound complexes. This structural feature is consistent with the reported radical reactivity for the Mo nitrile complexes, which undergo bond-forming reactions involving the nitrile carbon atoms leading to the formation of ketimide linkages.^9^

### DFT-Computed Thermochemical Data for Ligand Binding to **1** and **2**

**Scheme 2:**
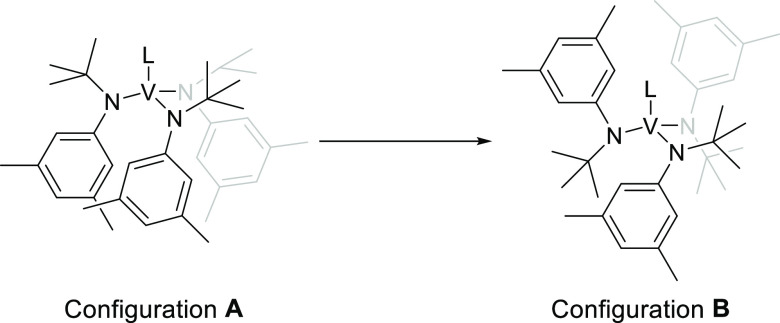
Interconversion between the **A** and **B** Configurations of Adducts of **2**

Since the incoming ligand can bind to **2** in configurations **A** or **B** (see Figure 6), both configurations were computed for selected adducts, and thermochemical data for their interconversion (Scheme 2) was derived.

The Gibbs energy changes for the **A** → **B** conversion are in the range from −2.2 to −3.4 kcal·mol^–1^ at 25 °C for the nitriles and isonitriles studied (L = MeCN, Me_2_NCN, PhCN, DFBN, and AdNC), as reported in Scheme S1 of the Supporting Information, and, consequently, the **B** configuration is favored upon ligand binding to **2**.

**Scheme 3:**
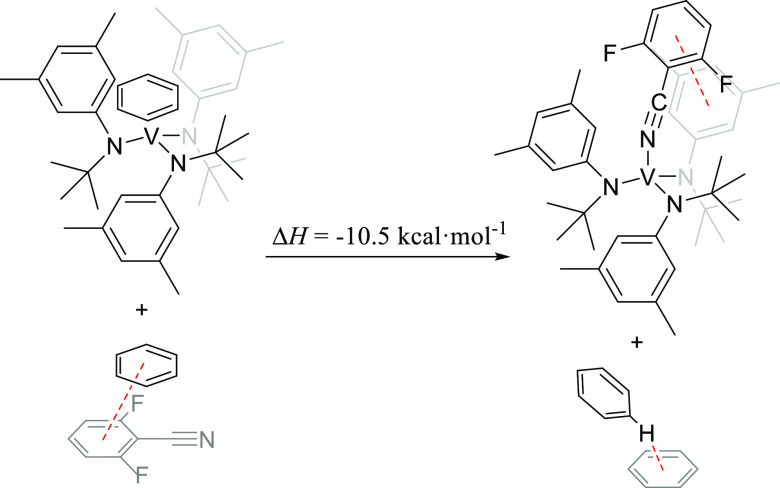
Thermochemical Values for the Reaction of the Most Stable Structure from Figure 7 and DFBN Containing a π-Stacking Interaction with a Benzene Molecule to Yield Complex DFBN-**2** and the Most Stable Tilted T-Shape Benzene Dimer^33^

A summary of computed thermochemical data for ligand binding to **1** and **2** is shown in Table S22 of the Supporting Information. A reasonable correlation is seen between calculated and experimental enthalpies of nitrile or isonitrile binding to **1** with differences lower than 3.5 kcal·mol^–1^. In the case of complexes of **2**, the computed data for Δ*H* reported with respect to the **A** configuration are more exothermic by ≈7 kcal·mol^–1^, as shown in Table S23 of the Supporting Information. However, as pointed out in a previous section, there is a stabilization of **2** by interaction with the solvent, and since it is known that fluorinated arenes exhibit enhanced intermolecular π-stacking interactions,^32^ thermochemical values for the reaction in Scheme 3 were also derived from DFT calculations to simulate better what is occurring in toluene solution in the binding of DFBN to **2**. As can be seen in Scheme 3, the derived value for the enthalpy of binding is −10.5 kcal·mol^–1^, which is in perfect agreement with that obtained experimentally (−10.4 ± 0.8 kcal·mol^–1^).

Likewise, a similar procedure has been employed to derive the enthalpies of binding for the rest of the ligands, and the values obtained are also in good agreement with the experimental data.

The computed enthalpy of binding of the nitriles studied to **2** spans a range of only 4 kcal·mol^–1^ as shown in Table S22 of the Supporting Information; however, the aryl nitrile binding to **1** is more sensitive to the substituent effect of the incoming ligand, and that range is expanded to 12 kcal·mol^–1^. In keeping with the larger range in enthalpies of nitrile binding to **1** compared to **2**, greater discrimination in qualitative binding studies is observed for **1** than for **2**.^2b,9,17,22,23^

### DFT-Computed Transition States for RCN Binding

**Figure 8:**
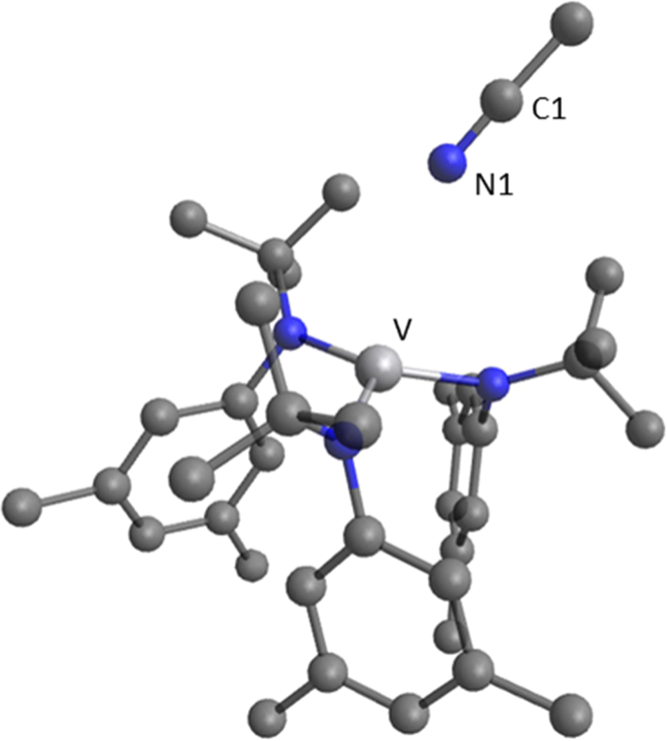
Optimized structure of the transition state for binding MeCN to **2** in the **A** configuration. Hydrogen atoms are omitted for clarity. Selected distances (Å) and angles (degrees): V–N1 = 3.358; N1–C1 = 1.156 Å; V–N1–C1 = 146.8°.

The kinetics of nitrile binding to **2** were also studied computationally using the simplest nitrile (MeCN) as a model. The transition state for the approach of MeCN to complex **2** in the **A** configuration was found, and its optimized structure is shown in Figure 8. The activation parameters derived (Δ*H*^‡^ = 2.5 kcal·mol^–1^ and Δ*S*^‡^ = −40.7 cal·mol^–1^·K^–1^) were in significant disagreement with the experimental data from Table 1 (Δ*H*^‡^ = 7.2 ± 0.3 kcal·mol^–1^ and Δ*S*^‡^ = −9 ± 2 cal·mol^–1^·K^–1^).

**Figure 9:**
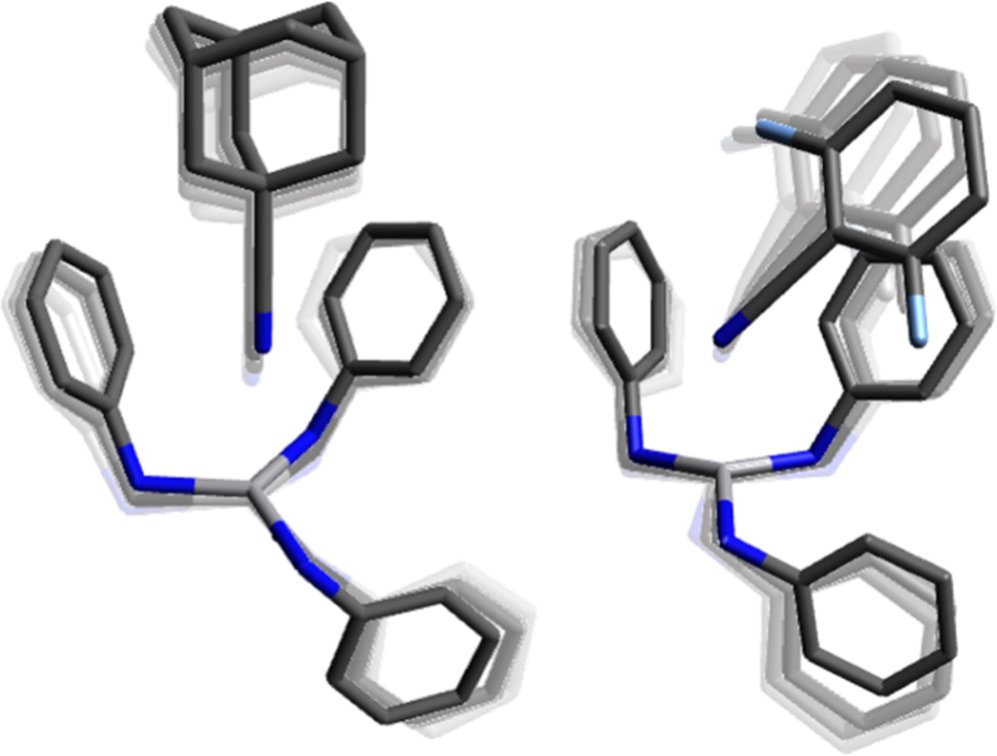
Overlay of optimized configurations at fixed V···N_nitrile_ distances (from 2 to 3 Å) for nitrile dissociation from AdCN-**2** (left) or DFBN-**2** (right) showing a linear dissociation of AdCN and a more angular trajectory for DFBN. *^t^*Bu groups, Me substituents in the aryl groups, and hydrogen atoms are omitted for clarity.

Attempts to locate the transition state for nitrile binding to **2** in the **B** configuration were unsuccessful. Search for the transition state structure for binding to **B** was made by use of the principle of microscopic reversibility. Starting with the established structure shown in Figure 1 for DFBN-**2** in the **B** configuration, a relaxed scan was performed along the V–N distance. The same procedure was repeated for AdCN, MeCN, and MesCN ligands and full data are available in Tables S24 and S25 in the Supporting Information. An overlay of selected optimized structures at fixed V···N distances is shown in Figure 9 for the release of AdCN and DFBN from AdCN-**2** and DFBN-**2**, respectively.

**Figure 10:**
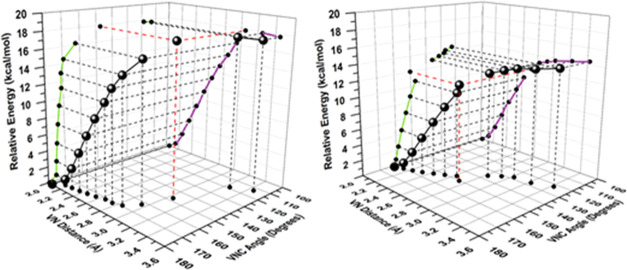
Energy (*z*-axis, kcal·mol^–1^), V–N_nitrile_ distance (*x*-axis, Å), and V–N–C angle (*y*-axis, degrees) for dissociation of AdCN (left) and DFBN (right).

A plot of energy (*z*-axis) vs V–N distance (*x-*axis) and V–N–C angle (*y-*axis) gives insight into the different pathways computed for AdCN and DFBN, as shown in Figure 10.

**Figure 11:**
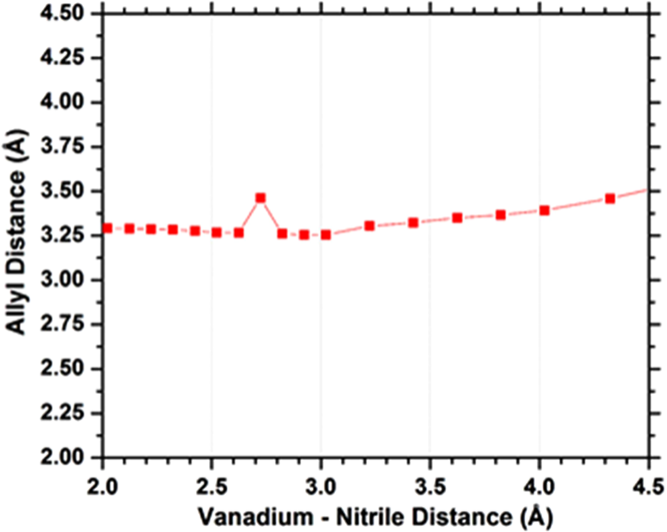
Plot of the center point distance between the top three C atoms of the arene of the anilide ligand and the bottom three C atoms of the arene of DFBN as a function of V···N distance.

There is a clear difference in the computed minimum energy pathways for nitrile dissociation shown in Figure 10 for AdCN-**2** and DFBN-**2**. In the case of AdCN, the V–N bond lengthens and the V–N–C angle remains almost linear along the reaction coordinate. In contrast, the computed pathway for DFBN shows that the V–N–C angle, which is somewhat angular to begin with, becomes increasingly bent during the dissociation process due to the establishment of an enthalpically favorable but entropically unfavorable π-stacking interaction between the DFBN and one of the Ar groups pendent to one of the anilide ligands. The arene–arene distance is computed to remain nearly constant as the DFBN ligand dissociates from DFBN-**2**, as shown in Figure 11. As the V–nitrile length increases by 2.5 Å, the arene–arene distance is computed to increase by 0.25 Å, approximately one-tenth the change.

### DFT-Computational Studies of MeCN and MeNC Binding to **1**′

**Figure 12:**
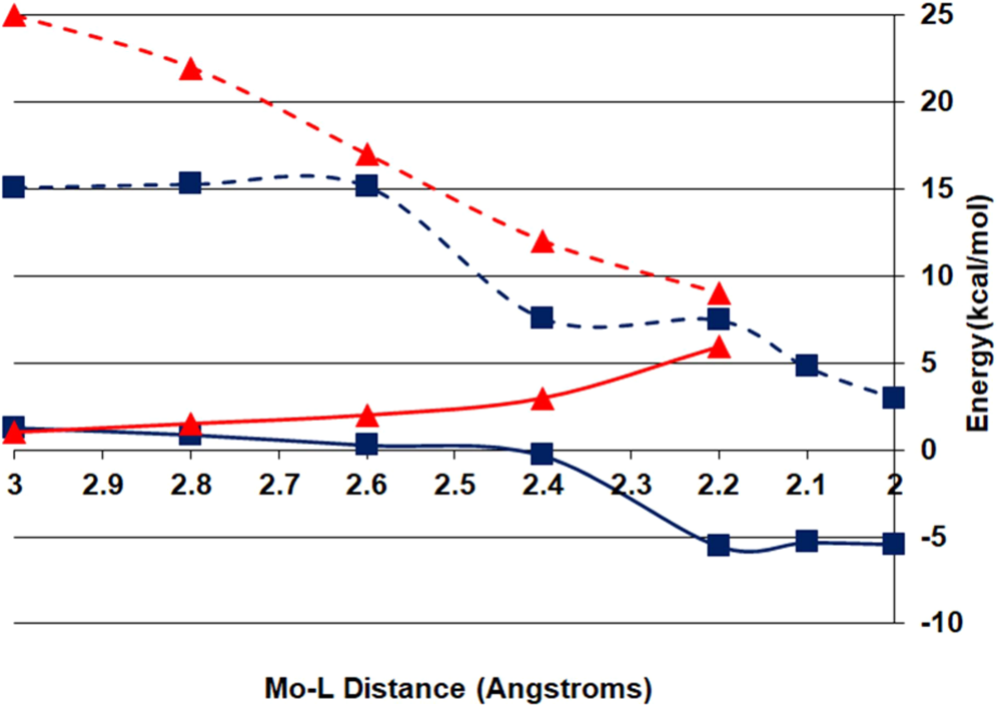
Computed energies as a function of distance for binding of MeNC (blue squares) and MeCN (red triangles) to **1**′ in the quartet (solid lines) and doublet (dashed lines) states.

As stated previously, Δ*G*^‡^(−40 °C) values for nitrile binding to **1** are approximately constant, whereas binding of AdNC to **1** exhibits a value of about 2 kcal·mol^–1^ lower. Furthermore, at −40 °C, the rate of binding of AdNC to **1** (*k* = 1.6 × 10^4^ M^–1^·s^–1^) is faster than to **2** (*k* = 1.0 × 10^4^ M^–1^·s^–1^), in clear contrast to the behavior observed for the rest of the ligands studied (see Table 1). Therefore, DFT calculations were performed in this work to explore the reasons for this surprising observation. For computational simplicity, relaxed scans were performed for the simplified complex **1**′ in which a phenyl group was used rather than the actual 3,5-Me_2_C_6_H_3_ aryl group in **1**. A model nitrile (MeCN) and isonitrile (MeNC) were studied as a function of the Mo···L distance. At each fixed Mo···L distance, the structure was optimized in the quartet state, and a single point energy calculation was also performed later in the doublet state on the frozen structure previously optimized (in the quartet state). The energy gap with no ligand present at all between the quartet and doublet state in the **A** configuration for **1** is computed to be 40 kcal·mol^–1^. This is due primarily to the “pairing energy” in going from essentially {d_*xz*_^1^d_*yz*_^1^d_*z*^2^_^1^} to {d_*xz*_^2^d_*yz*_^1^d_*z*^2^_^0^} to allow the formation of the Mo–L bond. The potential energy curves in both quartet and doublet states along the Mo···N_nitrile_ or Mo···C_isonitrile_ coordinate are shown in Figure 12.

Figure 12 shows how the quartet–doublet gap is reduced as MeCN approaches the **1**′ complex (red lines). However, while the quartet state curve rises continuously in energy as MeCN approaches the metal complex (solid red line), the quartet state in the case of MeNC binding decreases and at Mo–L distances below 2.4 Å becomes mildly exothermic (solid blue line). A transition state and a stable minimum for binding of MeNC to **1**′ in the high spin quartet state were found, and both structures are shown in Figures S24–S26 in the Supporting Information. Selected structural parameters are collected in Tables 2 and S26 of the Supporting Information. The formation of the intermediate in the quartet state from the reaction of MeNC and complex **1**′ is computed to be slightly exothermic but endergonic (see Table 2) and occurs with a low barrier of Δ*H*^‡^ = 1.8 kcal·mol^–1^. This value obtained for a truncated complex and ligand can be compared with that determined experimentally for the reaction of AdNC and complex **1** of Δ*H*^‡^ = 5.5 ± 0.5 kcal·mol^–1^ (see Table 1).

**Table 2:** Computed Mo···N or Mo···C Distances (Å), Mo–N–C or Mo–C–N Angles, Energies and Enthalpies (between Brackets) for the Transition State, Intermediate, and MECPs between the Quartet and Doublet Potential Energy Surfaces for Binding of MeCN or MeNC to **1**′

ligand	species	Mo–N/Mo–C (Å)	Mo–N–C/Mo–C–N (degree)	energy^c^ (kcal·mol^–1^)
MeCN				
	MECP^a^	2.276	154.1	6.0
	MECP^b^	2.312	154.2	3.2
MeNC				
	TS^a^	3.166	128.5	1.1[1.8]
	intermediate^a^	2.099	168.2	–6.0[−4.6]
	MECP^a^	2.069	167.6	–6.8

a Corresponding to an **A** configuration of **1**′.

b Corresponding to a **B** configuration of **1**′.

c Related to the two isolated fragments **1**′ (**A** configuration) + MeNC/MeCN.

The minimum energy crossing points (MECP)^34^ between the quartet and doublet potential energy surfaces for both MeCN and MeNC binding were also located, and their structures are shown in Figure S27 in the Supporting Information. Selected structural and energetic parameters for these species are also collected in Table 2. In all cases, optimizations in the doublet state using these structures as starting points lead to the final MeCN-**1**′ or MeNC-**1**′adducts. However, optimizations in the quartet state lead to the dissociation of the nitrile or formation of the high spin minimum described above for the isonitrile (see Figure S25).

## Discussion

The kinetic data reported for the binding of nitriles to **1** and **2** do not differ that greatly, but the processes, absolute energies, and computed mechanisms do. Experimental and computational errors and uncertainties in intimate solvation energies of the complexes studied caution against over-interpretation of the data in this work. In spite of these difficulties, a consistent picture emerges for the ligand binding mechanisms for **1** and **2**.

### Solution Phase Configurations of **1** and **2**

The metal–ligand bond formation between a metal complex and a two-electron σ-donor ligand requires the presence of a vacant binding site at the metal center. Completely vacant sites are usually occupied by a weak ligand that must dissociate prior to binding or by unpaired electrons located in semi-occupied molecular orbitals (SOMOs) that must pair and, consequently, the molecule must undergo an electronic change of state. Therefore, a key point needed for a complete understanding of the binding process to complexes **1** and **2** is the knowledge of their structures in the solution phase.

The exact configuration of **2** in toluene solution is not known with certainty. DFT calculations predict a close-lying equilibrium between configurations **A** and **B** (see Figure 6), with a low barrier for interconversion between them, as shown in Figure S23 in the Supporting Information. Moreover, thermodynamically favorable interactions between the aromatic solvent and the **B** configuration of complex **2** (see Figure 7) were computed by DFT calculations, and the enthalpy calculated for DFBN binding is in excellent agreement with that which was experimentally determined when the interaction with the solvent for both metal complex and free nitrile are considered in the calculations as depicted in Scheme 3. Thus, it is most likely that solvated structures of **A** and **B** are the dominant species in solution. Either solvent displacement (see Figure 7) or removal of the V–allyl interaction present in the **B** structure (see Figure 6) is needed to add a ligand. Both processes are endothermic but entropically favored steps. This accounts for the lower entropies of activation generally observed for nitrile addition to **2** compared to **1**, as evidenced by the values collected in Table 1. Furthermore, the measured entropy of DFBN binding to **2** in toluene solution (Δ*S* = −26 ± 4 cal·mol^–1^·K^–1^) is a less negative value than expected for this bimolecular reaction, again supporting the requirement of either solvent displacement or opening of **B** by cleavage of the V–aryl interaction in the binding process. For comparison purposes, the entropy of binding of PhCN and MesCN to **1** were measured previously as −40 ± 5 and −52 ± 5 cal·mol^–1^·K^–1^, respectively.^2b^ The authors do not consider that the **A** configuration unblocked by the solvent is present in the solution. If it were, Me_2_NCN would bind with little or no enthalpic barrier to form Me_2_NCN-**2** in the **A** configuration, the structure of which is shown in Figure 1. In line with this presumption are the activation parameters computed for MeCN binding to **2** in its **A** configuration with Δ*H*^‡^ = 2.5 kcal·mol^–1^ and Δ*S*^‡^ = −40.7 cal·mol^–1^·K^–1^ in significant disagreement with the experimental data of Δ*H*^‡^ = 7.2 ± 0.3 kcal·mol^–1^ and Δ*S*^‡^ = −9 ± 2 cal·mol^–1^·K^–1^ (see Table 1). Furthermore, the lack of agreement between the experimental and thermodynamic values computed for ligand binding to **2** assuming an unblocked **A** configuration with lower enthalpies and much more negative entropies computed (Table S23 in the Supporting Information) also seems to indicate the existence of some sort of association of complex **2** in toluene solution.

In contrast, in complex **1**, the three SOMO electrons in the d*_xz_*, d*_yz_*, and d_*z*^2^_ orbitals effectively block the approach of both ligands and the solvent to the metal center. The major event needed to occur to bind in this complex is to remove one of the electrons either by a spin state change at the metal or by electron transfer to the incoming ligand. This is a major energetic event on the order of 40 kcal·mol^–1^, as discussed earlier. The nature of the incoming ligand is intimately involved in lowering this high barrier, as shown in Figure 12. The binding of ^•^NO occurs at a rate too rapid to measure with conventional stopped-flow techniques and is trapped in the **A** configuration.^35^ In this case, there is no need to generate a vacant orbital due to spin annihilation.

### Nitrile and Isonitrile Binding to **2**

**Figure 13:**
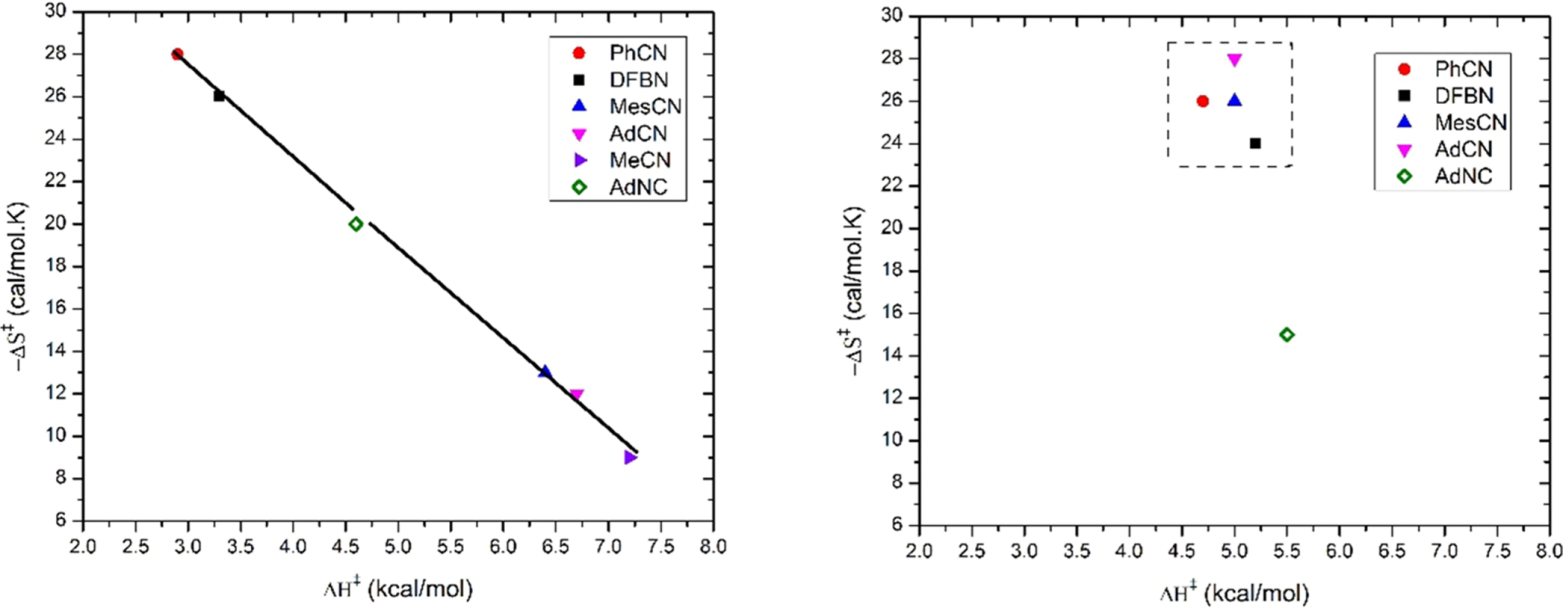
Plot of −Δ*S*^‡^ (cal·mol^–1^·K^–1^) vs Δ*H*^‡^ (kcal·mol^–1^) for binding of nitriles to **2** (left) and **1** (right). All data are for nitriles, except for AdNC (green diamond).

Kinetic data reveal a ca. 100-fold faster nitrile coordination to **2** compared to **1**. The reaction rates observed for nitrile binding to **2** are remarkable because second-order rate constants on the order of 10^3^ M^–1^·s^–1^ were observed at low temperatures (from −62 to −35 °C), and reactions were often complete in less than a second. The kinetic data for ligand binding to **2** at −40 °C collected in Table 1 show that there is a nearly constant Δ*G*^‡^(−40 °C) = 9.3 ± 0.3 kcal·mol^–1^ for all ligands studied. This simple observation masks a more complex behavior, and it does not carry over to its Δ*H*^‡^ and Δ*S*^‡^ components, as shown in Figure 13.

The data for binding to **2**, including the isonitrile AdNC, all fall on a line, often referred to as isokinetic behavior. If the point for the isonitrile AdNC is removed in Figure 13 (it is sufficiently different from a nitrile that this is warranted), the remaining “points” are clustered at two extremes with a large gap in the middle. These may be separated into two limiting categories: a first class (PhCN and DFBN) with low enthalpic barriers but very unfavorable entropies of activation (Δ*H*^‡^ ≈ 3 kcal·mol^–1^ and Δ*S*^‡^ ≈ −27 cal·mol^–1^·K^–1^) and a second class (MesCN, AdCN, and MeCN) with higher Δ*H*^‡^ values but less unfavorable entropies of activation (Δ*H*^‡^ ≈ 6.8 kcal·mol^–1^ and Δ*S*^‡^ ≈ −11 cal·mol^–1^·K^–1^).

A mechanistic dichotomy of this type is common where an associative and a competing dissociative pathway are present for ligand substitution.^36^ The ligands which have a lower value for Δ*H*^‡^ and more negative entropy of activation would be consistent with an associative mechanism, while the ligands with a more positive Δ*H*^‡^ and a less negative Δ*S*^‡^ would be consistent with a mechanism with higher dissociative character.

The steric and electronic profiles of both classes of ligands showed no clear characteristic to differentiate them as stronger or weaker donors or as more or less sterically encumbered. However, the common feature of the first class of ligands, PhCN and DFBN, is their better ability to form a π-stacking interaction like that observed in the structure of DFBN-**2** shown in Figure 1. Nevertheless, this kind of interaction is not restricted to the structure of the final adduct but can be established along the entire nitrile addition path. Consequently, the presence of this kind of stabilizing interaction at the transition state for the ligands capable of establishing this interaction would serve to reduce its energy when compared to those ligands for which this stabilization is weaker or not possible. Figure 11 provides a convincing picture supporting the retention of the π-stacking interaction in the nitrile dissociation from DFBN-**2**, with the arene–arene distance computed to remain nearly constant as DFBN dissociates.

The differing scenarios for ligand binding to **2** can be now readily explained in a simple proposed mechanism. In both classes of ligands, an approach to the transition state is proposed to involve solvent displacement to clear the path for the nitrile approach. For PhCN and DFBN ligands, as discussed previously, a π-stacking interaction is established during the binding process that leads to a reduction of the enthalpy of activation while disfavoring the process entropically, in keeping with the position of DFBN and PhCN in Figure 13 (lower activation enthalpies and more negative activation entropies). The rest of the ligands in Figure 13 (MesCN, AdCN, and MeCN) establish weaker dispersion contacts than the π-stacking interactions exhibited in the binding of PhCN and DFBN to **2**. Accordingly, the transition state is located at higher energy values but with a less unfavorable entropy of activation, in agreement with the kinetic values determined for this class of ligands and their position in Figure 13.

There is an advantage at low temperatures for the class of ligands able to establish a stabilizing π-stacking interaction during the binding event. They have more unfavorable entropies of activation, and the slope of the Eyring plots (see Supporting Information Figure S18) shows that since there is nearly an intersection of all of the points at −40 °C at lower temperatures, they bind at faster rates than the other ligands do.

Finally, the binding of AdNC to **2** occurs in a slightly different position compared to AdCN, but it is not greatly displaced and does not bind significantly faster than the nitriles. There are large electronic differences between nitrile and isonitrile ligands, and speculation regarding this question is not warranted.

### Binding of Nitriles and Isonitriles to **1**

As in the case of ligand binding to **2**, the Gibbs energy barrier for nitrile coordination to **1** at −40 °C is almost constant (Δ*G*^‡^(−40 °C) = 11.0 ± 0.5 kcal·mol^–1^, see Table 1), but the activation parameters for ligand binding to **1** shown in Figure 13, all fit in a tight box near Δ*H*^‡^ = 5.0 kcal·mol^–1^ and Δ*S*^‡^ = −26 cal·mol^–1^·K^–1^ with the exception of the isonitrile AdNC. The doublet state of complex **1** is ≈40 kcal·mol^–1^ higher in energy than the quartet state, and this large gap must be lowered to achieve the intersystem crossing needed, which serves to clear the d_*z*^2^_ orbital for binding the nitrile (see Figure 12). The fact that all of the nitrile ligands have similar enthalpies and entropies of activation and appear in a tight box in Figure 13 indicates that, in terms of altering the energy gap between the quartet and doublet states, the R group of the R–C≡N does not change things much for the nitriles studied here. The probable reason for this is that there is an essentially common barrier to intersystem crossing for nitrile ligands. There is overall good agreement between the experimental and results obtained by DFT calculations. The computed barriers to achieving the structures of the MECP between the quartet and doublet potential energy surfaces for MeCN binding to **1**′ (6.0 or 3.2 kcal·mol^–1^, respectively, for the **A** and **B** configurations of **1**′, Table 2) are in excellent agreement with the enthalpy of activation previously measured for other nitriles (Δ*H*^‡^ = 4.7 – 6.4 kcal·mol^–1^, see Table 1 and Figure 13). The kinetics of binding MeCN cannot be studied experimentally since the addition of MeCN to **1** results in rapid reductive nitrile coupling, yielding the corresponding diiminato species [μ-NC(Me)C(Me)N][Mo(N[*^t^*Bu]Ar)_3_]_2_ (Scheme 1).^9^

The rate of addition of AdNC to **1** is faster than that of nitriles, and in fact, it is the only ligand studied in this report that was found to bind faster to **1** than to **2**. The isonitrile AdNC binds to complex **1** with Δ*G*^‡^(−40 °C) = 9.0 kcal·mol^–1^, which is about 2 kcal·mol^–1^ lower than the binding of nitriles to the same complex.

A surprising picture emerged from computational DFT studies showing that the energy of the quartet state does not increase but decreases as an isonitrile approaches the Mo center. This is attributed to the more favorable electron transfer from the metal center to the “forward-leaning” π* orbital lobe on the C atom of the incoming isonitrile ligand, altering the energetics of its approach and accounting for the quartet state being attractive rather than repulsive. This proposal is supported by the nature of the SOMOs of the minimum found in the quartet state, as shown in Figure S28 of the Supporting Information, with a substantial overlap between the d orbitals of the metal and the π* orbitals of the coordinated isonitrile. The isomeric nitriles have a “rearward leaning” π* orbital lobe on the C, which trails the N atom of the nitrile as it approaches the metal center. The MECP between the quartet and doublet potential energy surfaces for MeNC binding to **1**′ was also located, and its energy is −6.8 kcal·mol^–1^ related to the two separate fragments. However, a transition state occurring in the quartet state prior to the MECP at a long Mo···CNR distance (3.166 Å, Table 2) was also found, and the kinetic barrier for isonitrile binding is proposed to be associated with the requirement to overcome this transition state. This proposal is consistent with the less negative entropy of activation measured experimentally for AdNC binding to **1** (Δ*S*^‡^ = −15 ± 4 cal·mol^–1^·K^–1^) as compared to the corresponding value determined for AdCN (Δ*S*^‡^ = −28 ± 5 cal·mol^–1^·K^–1^) as collected in Table 1.

## Conclusions

Greater stability of the nitrile adducts with a d^2^-metal with respect to radical couplings at the carbon atom of the C≡N bond allowed for isolation and structural characterization of the end-on (η^1^) complexes of **2** and for unambiguous measurements of the energetics and kinetics of the end-on coordination of small molecules with an element–nitrogen triple bond (RCN and RNC).

Nitrile adducts of **2** are dominated by σ-donor interactions; in contrast, π back-donation plays an important role in nitrile binding to **1**. Nevertheless, very fast and exothermic binding of nitriles to **2** was observed. The more pronounced electrophilic character of a d^2^ ion, V(III), accounts for the tendency of vanadium to undergo very rapid ligand exchange reactions with reaction rates higher for nitrile binding to **2** than to **1**. Similar reaction rates and activation parameters were found for a series of aromatic and aliphatic nitriles with Δ*G*^‡^(−40 °C) values clustered at 9.3 ± 0.3 or 11.0 ± 0.5 kcal·mol^–1^ for binding to **2** or to **1**, respectively. However, the rather similar rate and activation parameters mask a complex set of factors that must be considered in the reaction mechanism of nitrile binding to these V(III) or Mo(III) complexes. In the case of nitrile binding to **1**, this process is affected by the requirement to achieve the structure of the MECP between the quartet and doublet potential energy surfaces, and this is not very sensitive to the R substituent in the RCN nitrile as supported by the almost constant reaction rates and activation parameters measured. However, the behavior is clearly different in nitrile binding to **2**, where marked differences have been observed for aromatic nitriles able to establish a π-stacking, stabilizing interaction between the arene substituent in the incoming nitrile and the pendant arene of an anilide ligand present in **2**. This interaction is not restricted to the final adduct, as observed in the crystal structure of DFBN-**2** shown in Figure 1, but occurs in the entire binding event, as evidenced in Figures 9 and 11. Consequently, low enthalpies of activation and unfavorable entropies of activation (Δ*H*^‡^ ≈ 3 kcal·mol^–1^ and Δ*S*^‡^ ≈ −27 cal·mol^–1^·K^–1^) were measured for binding of the nitriles able to establish this interaction (PhCN and DFBN), while higher enthalpic barriers but a less unfavorable activation entropies (Δ*H*^‡^ ≈ 6.8 kcal·mol^–1^ and Δ*S*^‡^ ≈ −11 cal·mol^–1^·K^–1^) were experimentally determined for the rest of nitriles (MesCN, AdCN, and MeCN). The important conclusion is that at very low temperatures, the associative binding through the initial establishment of a π-stacking interaction or other stabilizing dispersion interaction may aid in both the thermodynamic and kinetic aspects of nitrile binding for suitable nitriles.

While adamantyl isonitrile binding to **2** is also very fast (in fact, somewhat faster than the reaction of the corresponding nitrile, AdCN), AdNC binding to **1** is even faster, reversing the typical V > Mo trend for relative rates of ligand binding to these two metal centers. This surprising behavior is due to the different character of the quartet potential energy surface for ligand binding being mildly attractive for the isonitrile approach to **1** while mildly repulsive for nitrile binding, as shown in Figure 12. The attractive character of the former is in line with the more favorable electron transfer from the metal center to the “forward-leaning” π* orbital lobe on the C atom of the isonitrile. Consequently, in the isonitrile binding, the transition state is located in the quartet state and with the ligand far away from the metal center in agreement with the less unfavorable entropy of activation measured.

This work highlights the different nature of the binding site at the Mo(III) and V(III) metal centers in complexes **1** and **2** and also highlights the emerging importance of weak interactions, including the arene–arene interaction present in aryl nitrile binding to **2** playing a role in the mechanism. The differing activation parameters for binding of nitriles to **2** typically point to an associative process in which the incoming ligand partially binds to the metal center as it prompts displacement of the outgoing ligand. This case is proposed to be different. The associative nature is attributed to the establishment of an arene–arene binding interaction as the basis for the different reaction pathways. This is achieved at a site relatively remote from the metal center. This type of effect is easy to overlook in strongly bound ligands, where the binding energies are dominant. It is precisely for weakly bound ligands such as nitriles where such interaction may be observed to alter the binding trajectory. Additional studies aimed at more fully elucidating the role dispersion interactions can play in the kinetics of binding and activation of other weak ligands are in progress.

## Supporting Information Available

Experimental details data for the synthesis of PhCN-**2**, DFBN-**2**, Me_2_NCN-**2**, and [PhCN-**2**][BAr_4_^F^]; FTIR study of binding of DFBN to **2**; stopped-flow kinetic measurements; X-ray crystallography; and computational details (PDF)
All computed molecule Cartesian coordinates (XYZ)

## Supplementary Material

ic3c00595_si_001.pdf

ic3c00595_si_002.xyz
