# Supplementary material for: Binding of Nitriles and Isonitriles to V(III) and Mo(III) Complexes: Ligand vs Metal Controlled Mechanism
Source: Inorg Chem. 2023 Jun 28;62(27):10559–71. doi: 10.1021/acs.inorgchem.3c00595 (PMC10336974; doi:10.1021/acs.inorgchem.3c00595)
Supplement: Supplementary file 1 — ic3c00595_si_001.pdf [file ic3c00595_si_001.pdf]

Supporting Information of the article:

Binding of nitriles and isonitriles to V(III) and Mo(III) complexes: Ligand vs metal controlled mechanism

Taryn D. Palluccio,[a] Meaghan E. Germain,[a] Marco Marazzi,[b,c] Manuel Temprado,[b,c] Jared S. Silvia,[d] Peter Mueller,[d] Christopher C. Cummins,[d]* Jack V. Davis,[e] Leonardo F. Serafim,[e] Burjor Captain,[e] Carl D. Hoff,[e]* Elena V. Rybak-Akimova[a]*†*

[a] Department of Chemistry, Tufts University, Medford, Massachusetts 02155, United States.

[b] Universidad de Alcalá, Departamento de Química Analítica, Química Física e Ingeniería Química, Grupo de Reactividad y Estructura Molecular (RESMOL), Alcalá de Henares, Madrid, Spain.

[c] Universidad de Alcalá, Instituto de Investigación Química ''Andrés M. del Río'' (IQAR), Alcalá de Henares, Madrid, Spain.

[d] Department of Chemistry, Massachusetts Institute of Technology, Cambridge, Massachusetts, 02139, United States.

[e] Department of Chemistry, University of Miami, Coral Gables, Florida 33146, United States.

† Deceased March 11, 2018

Corresponding Authors

* Manuel Temprado - manuel.temprado@uah.es

* Christopher C. Cummins - ccummins@mit.edu

* Carl D. hoff - c.hoff@miami.edu

* Elena V. Rybak-Akimova - elena.rybak-akimova@tufts.edu

Contents

General considerations……….....……………………………………………………………S3

Synthesis of PhCN-V(N[^{t}Bu]Ar)$_3$ (PhCN-**2**)……….....…………………………………………..S3

Synthesis of 2,6-$F_2C_6H_3$CN-V(N[^{t}Bu]Ar)$_3$ (DFBN-**2**)……….....………………………………..S4

Synthesis of Me_2NCN-V(N[^{t}Bu]Ar)$_3$ (Me_2NCN-**2**)……….....…………………………………..S5

Synthesis of [PhCN-V(N[^{t}Bu]Ar)$_3$][B(3,5-(CF_3)$_2C_6H_3$)$_4$] ([PhCN-**2**][BAr$^F_4$])…..………………S5

FTIR study of Binding of DFBN to **2** in Toluene Solution………………………………………S6

Calorimetric Measurement of Reaction of **2** with AdCN……………………………………..…S9

Stopped Flow Kinetic Measurements...……….....…………………………………..…………S9

X-ray Crystallography..……….....…………………………………………………………S23

Computational Details……….....…………………………………………………………..…S40

References……….....……………………………………………………………………….S55

General Considerations. Unless stated otherwise, all manipulations were carried out either in a Vacuum Atmospheres model MO-40M glovebox under an atmosphere of N_2 or using standard Schlenk techniques. Diethyl ether, *n*-pentane and *n*-hexane were degassed and dried using a solvent-purification system provided by Glass Contour. After purification, the solvents were stored under an atmosphere of N_2 over 4 Å molecular sieves. Deuterated benzene (Cambridge Isotope Labs) was dried by stirring over CaH_2 for 24 h and was subsequently vacuum-transferred onto 4 Å molecular sieves. $V(N[^tBu]Ar)_3$ ($Ar=3,5\text{-}Me_2C_6H_3$), **2**, was prepared according to literature methods.[1] $[(C_5H_5)_2Fe][B(3,5\text{-}(CF_3)_2C_6H_3)_4]$ ($FcBAr^F_4$) was prepared according to literature procedures.[2] All other reagents were used as supplied by the vendor without further purification. Celite 435 (EMD Chemicals), alumina (Aldrich) and 4 Å molecular sieves (Aldrich) were dried prior to use by heating at 200 °C for 48 h under dynamic vacuum. All glassware was oven dried at 220 °C prior to use. NMR spectra were obtained on either a Bruker 400-AVANCE spectrometer equipped with a Magnex Scientific superconducting magnet, or a Varian Mercury 300 spectrometer equipped with an Oxford Instruments Ltd. superconducting magnet. Proton NMR spectra were referenced to residual C_6D_5H (7.16 ppm).[3] IR spectra were collected on a Perkin-Elmer 2000 FT-IR spectrophotometer. Elemental analysis was performed by Midwest Microlab, Indianapolis, IN.

Synthesis of PhCN-V(N[^{t}Bu]Ar)$_3$ (PhCN-2). A solution of **2** (0.692 g, 1.19 mmol) in diethyl ether (~10 mL) was prepared. A solution of PhCN in diethyl ether (~2 mL) was prepared. The solution of PhCN (0.123 g, 1.19 mmol) was added to the stirring solution of **2** in a drop-wise manner. The color of solution changed from dark green-brown to a dark purple color. The reaction mixture was dried under reduced pressure. The resulting dark solids were extracted into *n*-hexane and the resulting solution filtered through a sintered glass frit. The solution was concentrated under

reduced pressure (~5 mL) and the solution was stored at −35 °C. After standing for 2 d, crystals had formed on the bottom of the vial. Decanting the mother liquid away from the crystals allowed for their isolation. These were washed with *n*-hexane (~1 mL) and dried under reduced pressure. Yield: 0.642 g of dark purple crystals (682.91 g/mol, 0.940 mmol, 78%). ^{1}H NMR (C_6D_6, 300 MHz, 20 °C) 24.7 (br), 13.9 (br), 3.5 (br), 1.01 (br), –1.47 (br), –31.1 ppm (br). IR: ν_{CN}(solid)= 2218 cm^{-1}; UV-vis(toluene): λ_{max}(toluene) = 490, 687 nm. Elemental Analysis: Calculated for $C_{43}H_{59}N_4V$: %C 75.63, %H 8.71, %N 8.20. Found: %C 75.82, %H 8.70, %N 8.04.

Synthesis of 2,6-$F_2C_6H_3$CN-V(N[^{t}Bu]Ar)$_3$ (DFBN-2). A solution of **2** (0.913 g, 1.57 mmol) in diethyl ether (~8 mL) was prepared. A solution of the 2,6-$F_2C_6H_3$CN (0.219 g, 1.57 mmol) in diethyl ether (~2 mL) was prepared. The solution of the nitrile was added to the stirring solution of **2** at 23 °C. The reaction mixture became deep purple in color. The reaction mixture was stirred for 1 h before the volatile components of the mixture were removed under reduced pressure. The purple residue was extracted into a 5:1 mixture of diethyl ether:*n*-pentane (~10 mL), filtered through a plug of Celite on a glass fiber filter, and then stored at −35 °C. After standing for 12 h, a small amount of crystalline material had precipitated from solution. The mother liquid was decanted away from the crystals that were then washed with *n*-pentane (1 mL). The crystals were dried under reduced pressure. The mother liquid was concentrated under reduced pressure (~5 mL) to effect the precipitation of the product. The mixture was then layered with *n*-pentane and stored at −35 °C. After standing for 48 h, the product was isolated as a purple powder on a sintered glass frit via vacuum filtration. The solids were dried under reduced pressure. Yield: 0.700 g (718.89 g/mol, 0.974 mmol, 62%). ^{1}H NMR (C_6D_6, 400 MHz, 20 °C) 31.0 (br), 14.2 (br), 1.12 (br), 0.77 ppm (br); IR: ν_{CN}(solid)= 2220 cm^{-1} (weak); UV-vis(toluene): λ_{max}(toluene) = 525, 687

nm. Elemental Analysis Calculated for $C_{43}H_{57}F_2N_4V$: %C 71.84, %H 7.99, %N 7.79. Found: %C 71.51, %H 7.86, %N 7.72.

Synthesis of Me_2NCN-V(N[^{t}Bu]Ar)$_3$ (Me_2NCN-2). A solution of Me_2NCN (0.079 g, 1.13 mmol) in diethyl ether (2 mL) was prepared. A solution of **1** (0.653 g, 1.13 mmol) in diethyl ether (10 mL) was prepared. The solution of cyanamide was added to the stirring solution of **2** at 23 °C using a pipet. The color of solution immediately changed from dark green to dark purple. After stirring for 30 min, the reaction mixture was concentrated to a volume of ~3 mL. Solids that had precipitated upon concentrating were redissolved by adding ~4 mL of a 5:1 diethyl ether:toluene solution to the reaction vessel. The reaction mixture was filtered through a plug of Celite on a glass fiber filter. The purple solution was allowed to stand at −35 °C. After standing for 12 hours, a dark, microcrystalline material had deposited on the bottom of the vial. The mother liquid was decanted away from the solids, and these were washed with 1 mL of *n*-pentane. The solids were dried under reduced pressure. Yield: 0.088 g. The mother liquid was dried under reduced pressure. The resulting blue solids were triturated with *n*-pentane (3 mL) to give a blue suspension. A bright blue powder was collected on a sintered glass frit and washed with *n*-pentane (2 x 1 mL). The solids were dried under reduced pressure. Yield: 0.518 g (649.88 g/mol, 0.93 mmol, 83% from both crops). ^{1}H NMR (C_6D_6, 400 MHz, 20 °C) 28.39 (br), 12.74 (br), 1.36 (br), 0.42 ppm (br); IR: ν_{CN}(solid) = 2263 cm^{-1} (intense); UV-vis: λ_{max}(toluene) = 560, 705 nm; Elemental Analysis of microcrystalline material: Calculated for $C_{39}H_{60}N_5V$: %C 72.08; %H 9.31; %N 10.78. Found: %C 71.90; %H 9.16; %N 10.40.

Synthesis of [PhCN-V(N[^{t}Bu]Ar)$_3$][B(3,5-(CF_3)$_2C_6H_3$)$_4$] ([PhCN-2][BAr$^F_4$]). A solution of PhCN (0.030 g, 0.29 mmol), **2** (0.169 g, 0.29 mmol) in diethyl ether (3 mL) was prepared and cooled to –35 °C. A suspension of FcBAr$^F_4$ (1 equiv.) in diethyl ether (3 mL) was

prepared and cooled to –35 °C. The solution of PhCN and **2** were subsequently added to the stirring suspension of $FcBAr^F_4$. The reaction mixture was allowed to warm to room temperature while stirring. The color of the solution changed from purple to dark green as the reaction progressed. After stirring at room temperature for 2 h, the reaction mixture was filtered through a plug of Celite on a glass fiber filter. The filter cake was washed with diethyl ether (1 mL). The filtrate solution was concentrated to a total volume of 2 mL and stored at –35 °C for 24 h. Dark green crystals could be isolated by decanting the mother liquor away, washing with *n*-pentane, and then drying under reduced pressure. Yield: 0.179 g (1546.14 g/mol, 0.11 mmol, 40%). ^{1}H NMR (C_6D_6, 400 MHz, 20 °C) 1.0 ppm (br); IR: ν_{CN}(nujol) = 2249 cm^{-1} (s); UV-vis: λ_{max}(toluene) = 495, 562 (sh), 687 nm; Elemental Analysis of microcrystalline material: Calculated for $C_{75}H_{71}BF_{24}N_4V$: %C 58.26; %H 4.63; %N 3.62. Found: %C 58.58; %H 4.83; %N 3.57.

FTIR study of Binding of DFBN to 2 in Toluene Solution. All studies were performed using standard inert atmosphere techniques. Toluene was dried by distillation from sodium benzophenone ketyl into flame dried glassware and stored under Argon. Solutions were prepared in a Vacuum-Atmospheres glove box, filtered, and loaded into a gas tight Hamilton syringe fitted with a syringe valve. Infrared Spectra were obtained on a Perkin Elmer series 2000 FTIR spectrometer with an MCT detector. The temperature of the FTIR cell was controlled by a water bath which was circulated through the jacketed cell, but also through copper tubing surrounding the cell which was housed in a small chamber fitted with CaF_2 windows. The temperature of the cell was measured by a precision thermistor obtained from Omega Engineering and cemented to the body of the FTIR cell. In addition, the temperature of the Argon atmosphere surrounding the cell in the small boxed area was measured with a Pt RTD. Spectroscopic data for nitrile binding are summarized in Table S1.

Table S1. Comparison of IR ν_{CN} band (in cm^{-1}) for RCN, RCN-**2** and RCN-**1**. All data in toluene or, in some cases, benzene solution. Between brackets ν_{CN}(bound)-ν_{CN}(free). For comparative purposes, data for the isonitrile AdNC are also included.

R	RCN/RNC	RCN-**2**/RNC-**2**	RCN-**1**[a]/RNC-**1**
Me_2N	2218	2256 [38]	---[b]
4-$Me_2NC_6H_5$	2216	2224 [8]	2019 [−197]
Ph	2230	2218 [−12]	2035 [−195][4]
4-$F_3CC_6H_4$	2234	2218 [−16]	2014 [−220]
C_6F_5	2224	2207 [−17]	2000 [−224]
DFBN	2242	2218 [−24]	1962 [−280]
AdNC	2129	2151 [22][5]	1762 [−367][6]

[a] Some data for RCN-**1** have been previously reported;[7] [b] Binding of Me_2NCN to **1** yields a side-on adduct.[8]

Representative FTIR data from one series of experiments is shown in Figure S1 and shows the increase in Absorbance of the band at 2216 cm^{-1} assigned to DFBN-**2** with decreasing temperature.

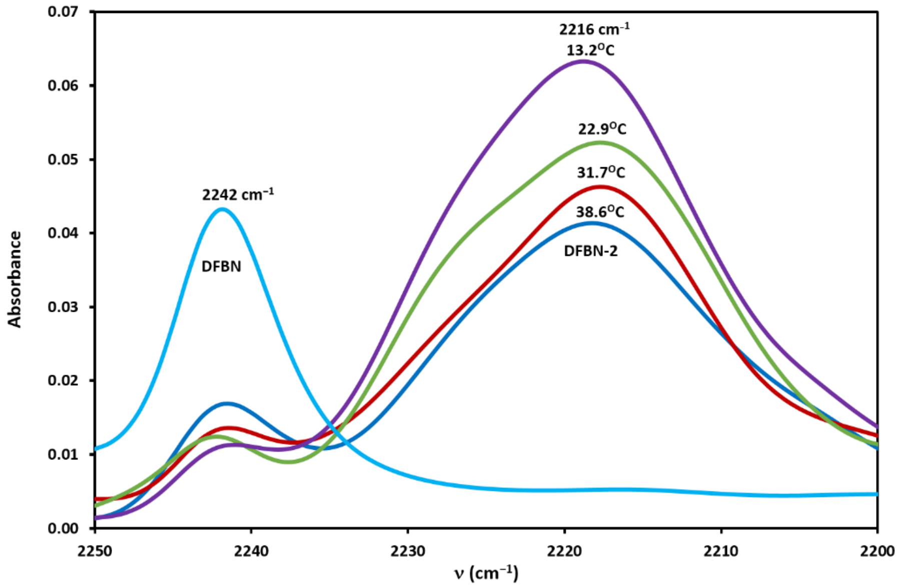

Figure S1. Variable temperature FTIR data for binding of DFBN to **2** in toluene. The broad nature of the band as well as an apparent shoulder may be due to different conformational isomers which may also be temperature dependent.

Due to broadening of the nitrile band with increasing temperature, equilibrium constants were computed based on peak areas rather than heights. FTIR studies were made in the overall temperature range of 9 to 49 °C. Plots of ln(K_{eq}) versus 1/T are shown in Figure S2.

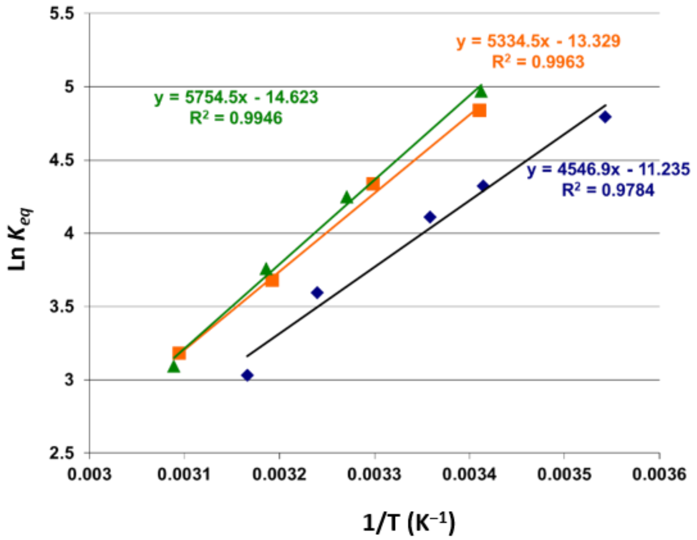

Figure S2. Plots of ln(K_{eq}) versus 1/T (K^{-1}) for binding of 2,6-$F_2C_6H_3CN$ to **2** in toluene. The data are for three separate runs with different stock solutions.

The derived enthalpies and entropies of binding for the three individual runs are collected in Table S1 and the average value was adopted.

Table S2. Thermochemical data for binding of DFBN to **2** derived from van't Hoff plots in Figure S2.

Run	1	2	3	Average
ΔH (kcal·mol^{-1})	−11.4	−10.6	−9.0	−10.4 ± 0.8
ΔS (cal·mol^{-1}·K^{-1})	−29.0	−26.5	−22.3	−26 ± 4

Calorimetric Measurement of Reaction of 2 with RCN. In the glovebox, a solution of 0.3424 g **2** (0.5906 mmol) was dissolved in 5 mL of distilled toluene and loaded via a syringe fitted with a PTFE syringe filter into the Calvet calorimeter cell. Solid adamantyl nitrile, AdCN, (0.0203 g, 0.126 mmol) was used as the limiting reagent and was analytically pure as verified by NMR spectroscopy. The calorimeter cell was sealed, taken from the glovebox, and loaded into the

Setaram C-80 calorimeter. Following temperature equilibration, the reaction was initiated and the calorimeter rotated to achieve mixing. Following return to baseline, the calorimeter cell was taken into the glovebox, opened, and 1mL of the solution loaded into an NMR tube. NMR spectra of both the stock solution and the calorimetry solution were then acquired and the reaction was confirmed as quantitative. The enthalpy of three measurements done in this way led to ΔH=−10.2 ± 0.3 kcal·mol^{-1} based on the reaction: **2** (tol. sol.) + AdCN (solid) → AdCN-**2** (tol. sol.). Using the enthalpy of solution of AdCN in toluene of +2.4 ± 0.2 kcal·mol^{-1} gives a value of −12.6 ± 0.5 kcal·mol^{-1} for the enthalpy of reaction with all species in toluene solution. Other calorimetric data was obtained using analogous techniques giving values of −14.4 ± 1.5 kcal·mol^{-1} and −16.4 ± 0.8 kcal·mol^{-1} for PhCN and Me_2NCN binding to **2** in toluene solution respectively.

Stopped Flow Kinetic Measurements. Anhydrous toluene (HPLC grade, ≥ 99.9%) was purchased from Sigma Aldrich and dried on an Innovative Technologies PureSolv 400 solvent purification system prior to use. Toluene solutions of **1** or **2** and substrates were prepared in an MBraun glove box filled with ultra-high purity argon (Airgas) and loaded in Hamilton gastight syringes. Time-resolved UV/visible spectra (λ = 400–800 nm) were acquired over a range of temperatures (–62 to –35 °C) using a Hi-Tech Scientific SF-61DX2 Multi-mixing CryoStopped-Flow system (TgK Scientific Ltd.) equipped with a Hi-Tech Scientific LHT50 tungsten light source, a J&M TIDAS diode array detector, and a Brandenburg 4479 Series PMT monochromator. The instrument was equipped with stainless steel plumbing lined with PEEK tubing and a 1.00 cm^3 quartz mixing cell submerged in an ethanol cooling bath. The temperature in the mixing cell was maintained to ± 0.1 °C. Data acquisition was performed using TIDAS-DAQ and/or Kinetic Studio software programs and mixing times were 2–3 ms. All flow lines were washed extensively with argon-saturated anhydrous toluene prior to charging the drive syringes with reactant solutions

and the driving syringe compartment was continuously flushed with argon during the experiments to preserve anaerobicity. Experiments were performed in a single-mixing mode of the instrument with a 1:1 (v/v) mixing ratio. Reactions were studied under pseudo-first order conditions using excess substrates. Data analysis was performed using Kinetic Studio (TgK Scientific) and IGOR Pro 5.0 (Wavemetrics, Inc.) software programs. All observed rate constants reported represent an average of at least three measurements which gave an acceptable standard deviation (within 10 %) and all remaining quantities derived from the kinetic data are reported with their standard deviations. All concentrations are reported after mixing in the stopped-flow cell.

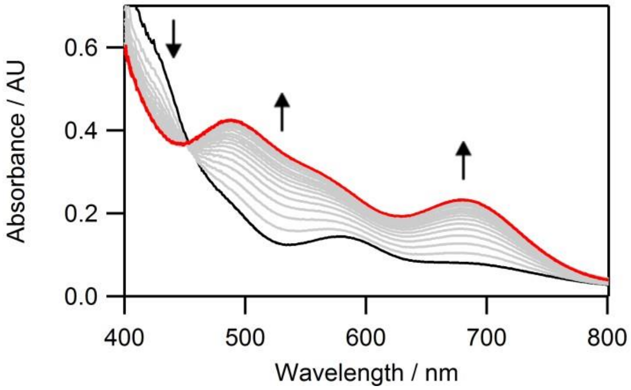

Figure S3. Time-resolved spectra of PhCN (1 mM) binding to **2** (0.3 mM) at −44 °C, acquired over 2 s. Selected traces shown for clarity. The initially recorded spectrum is shown in black and the final spectrum in red.

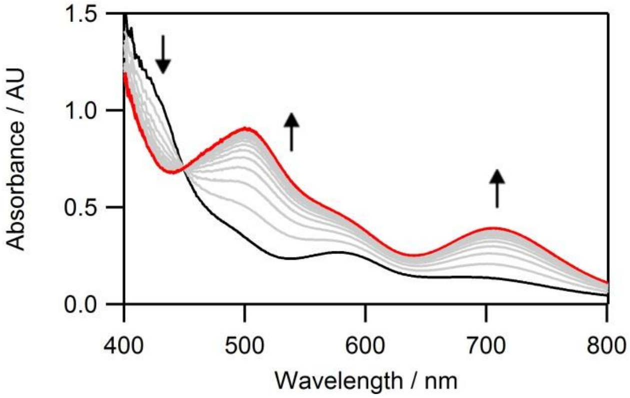

Figure S4. Time-resolved spectra of MesCN (1 mM) binding to **2** (0.3 mM) at −44 °C, acquired over 4 s. Selected traces shown for clarity. The initially recorded spectrum is shown in black and the final spectrum in red.

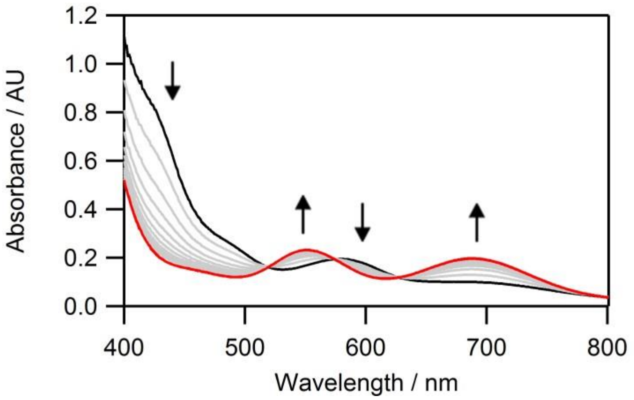

Figure S5. Time-resolved spectral changes accompanying the reaction between **2** (0.3 mM) and MeCN (1 mM) at −53 °C, acquired over 9 s. Selected traces shown for clarity. Initial spectrum is shown in black and final spectrum in red.

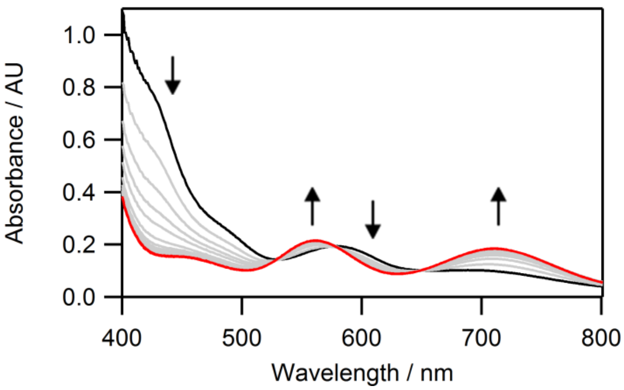

Figure S6. Time-resolved spectral changes accompanying the reaction between **2** (0.3 mM) and Me_2NCN (2 mM) at −62 °C, acquired over 12 s. Selected traces shown for clarity. Initial spectrum is shown in black and final spectrum in red.

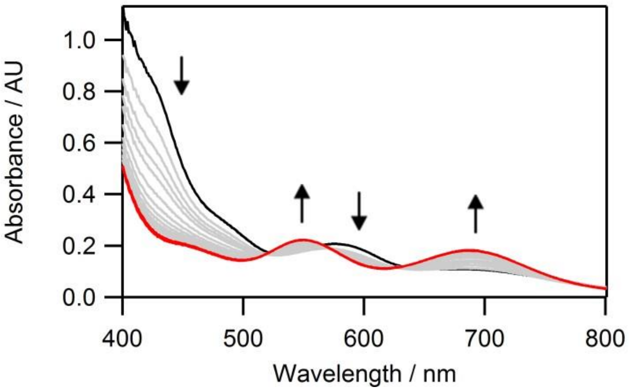

Figure S7. Time-resolved spectral changes accompanying the reaction between **2** (0.3 mM) and AdCN (1 mM) at −62 °C, acquired over 9 s. Selected traces shown for clarity. Initial spectrum is shown in black and final spectrum in red.

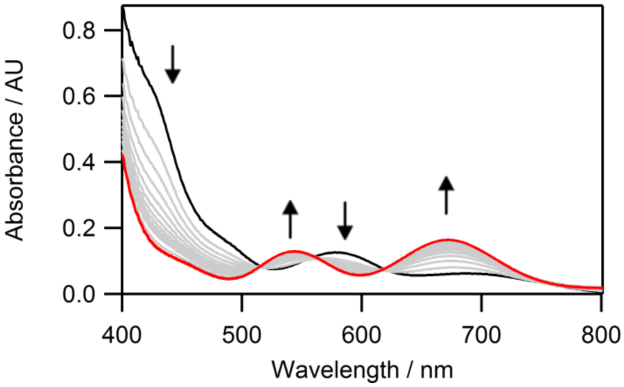

Figure S8. Time-resolved spectral changes accompanying the reaction between **2** (0.3 mM) and AdNC (1 mM) at −62 °C, acquired over 9 s. Selected traces shown for clarity. Initial spectrum is shown in black and final spectrum in red.

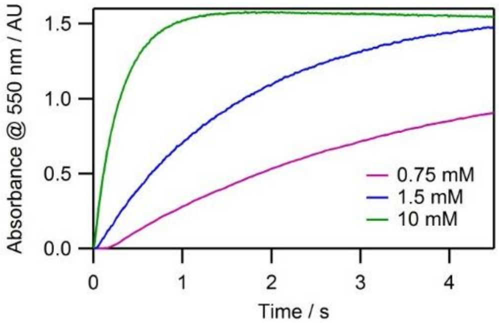

Figure S9. Overlay of selected kinetic traces at λ = 550 nm highlighting formation of the DFBN-**1** adduct at −40 °C as a function of [DFBN]. Reactions acquired in single wavelength mode with $[\mathbf{1}]_0$ = 0.15 mM.

Table S3. Temperature dependent pseudo-first order (k_{obs}) and second order (k_1) rate constants for adduct formation measured at λ = 687 nm with 0.3 mM **2** and varying [DFBN]. Kinetic traces at the two lowest temperatures (−62 and −53 °C) were fit using a biexponential function; only k_{1obs} was analyzed as k_{2obs} showed no dependence on [DFBN] and could arise from decomposition of the highly air- and moisture-sensitive complex. All remaining kinetic traces were fit using a single exponential function.

−62 °C	[DFBN] (M)	k_{obs} (s^{-1})	k_1 ($\times 10^3$ M^{-1} s^{-1})
	0.001	3.21	
	0.002	7.54	3.41 ± 0.12
	0.005	16.2	
	0.010	34.4	
−53 °C	[DFBN] (M)	k_{obs} (s^{-1})	k_1 ($\times 10^3$ M^{-1} s^{-1})
	0.001	5.05	
	0.002	9.17	4.93 ± 0.14
	0.005	23.0	
	0.010	49.2	
−44 °C	[DFBN] (M)	k_{obs} (s^{-1})	k_1 ($\times 10^3$ M^{-1} s^{-1})
	0.001	9.65	
	0.002	16.6	6.55 ± 0.05
	0.005	35.6	
	0.010	68.8	
−35 °C	[DFBN] (M)	k_{obs} (s^{-1})	k_1 ($\times 10^3$ M^{-1} s^{-1})
	0.001	17.3	
	0.002	25.8	9.59 ± 0.18
	0.005	57.0	
	0.010	103	

Table S4. Temperature dependent pseudo-first order (k_{obs}) and second order (k_1) rate constants for product formation at λ = 687 nm with 0.3 mM **2** and varying [PhCN]. Kinetic traces at −62 °C) were fit using a biexponential function; only k_{1obs} was analyzed as k_{2obs} showed no dependence on [PhCN] and could arise from decomposition of the highly air- and moisture-sensitive complex. All remaining kinetic traces were fit using a single exponential function.

−62 °C	[PhCN] (M)	k_{obs} (s^{-1})	k_1 (×10^3 M^{-1} s^{-1})
	0.001	2.09	3.85 ± 0.06
	0.002	5.96	
	0.005	16.8	
	0.010	36.9	
**−53 °C**	**[PhCN] (M)**	**k_{obs} (s^{-1})**	**k_1 (×10^3 M^{-1} s^{-1})**
	0.001	4.9	5.16 ± 0.20
	0.002	9.04	
	0.005	22.3	
	0.012	61.2	
**−44 °C**	**[PhCN] (M)**	**k_{obs} (s^{-1})**	**k_1 (×10^3 M^{-1} s^{-1})**
	0.001	6.4	6.14 ± 0.07
	0.002	12.2	
	0.005	30.1	
	0.010	61.6	
**−35 °C**	**[PhCN] (M)**	**k_{obs} (s^{-1})**	**k_1 (×10^3 M^{-1} s^{-1})**
	0.001	12.4	10.1 ± 0.2
	0.002	22.9	
	0.005	50.6	
	0.010	104	

Table S5. Temperature dependent pseudo-first order (k_{obs}) and second order (k_1) rate constants for adduct formation measured at λ = 705 nm (formation) with 0.3 mM **2** and varying [MesCN]. Kinetic traces were fit using either a single or biexponential function; when biexponential fits were employed, only k_{1obs} was analyzed as k_{2obs} showed no dependence on [MesCN] and was not reproducible.

−62 °C	[MesCN] (M)	k_{obs} (s^{-1})	k_1 ($\times10^3$ M^{-1} s^{-1})
	0.001	1.93	
	0.002	3.76	1.39 ± 0.03
	0.005	7.78	
	0.010	14.6	
−53 °C	[MesCN] (M)	k_{obs} (s^{-1})	k_1 ($\times10^3$ M^{-1} s^{-1})
	0.001	3.90	
	0.002	5.73	3.08 ± 0.10
	0.005	16.2	
	0.010	31.1	
−44 °C	[MesCN] (M)	k_{obs} (s^{-1})	k_1 ($\times10^3$ M^{-1} s^{-1})
	0.001	5.54	
	0.002	10.6	5.92 ± 0.08
	0.005	28.2	
	0.010	58.5	
−35 °C	[MesCN] (M)	k_{obs} (s^{-1})	k_1 ($\times10^3$ M^{-1} s^{-1})
	0.001	11.3	
	0.002	21.5	9.02 ± 0.21
	0.005	50.1	
	0.010	92.8	
−26 °C	[MesCN] (M)	k_{obs} (s^{-1})	k_1 ($\times10^3$ M^{-1} s^{-1})
	0.001	18.8	
	0.002	34.6	15.5 ± 0.2
	0.005	79.4	
	0.010	159	

Table S6. Temperature dependent pseudo-first order (k_{obs}) and second order (k_1) rate constants for product formation measured at λ = 687 nm with 0.3 mM **2** and varying [MeCN]. Formation traces were fit to a biexponential equation at −62 °C with only k_{1obs} showing a dependence on [MeCN]. Formation traces at all remaining temperatures fit well to a single exponential equation.

−62 °C	[MeCN] (M)	k_{obs} (s^{-1})	k_1 ($\times 10^3$ M^{-1} s^{-1})
	0.001	2.08	
	0.002	4.28	1.79 ± 0.06
	0.005	8.86	
	0.010	18.4	
−53 °C	[MeCN] (M)	k_{obs} (s^{-1})	k_1 ($\times 10^3$ M^{-1} s^{-1})
	0.001	4.37	
	0.002	9.00	3.58 ± 0.08
	0.005	19.4	
	0.010	37.0	
−44 °C	[MeCN] (M)	k_{obs} (s^{-1})	k_1 ($\times 10^3$ M^{-1} s^{-1})
	0.001	7.47	
	0.002	19.9	8.05 ± 0.46
	0.005	45.4	
	0.010	81.4	
−35 °C	[MeCN] (M)	k_{obs} (s^{-1})	k_1 ($\times 10^3$ M^{-1} s^{-1})
	0.001	13.9	
	0.002	36.9	13.7 ± 0.7
	0.005	74.8	
	0.010	141	

Table S7. Temperature dependent pseudo-first order (k_{obs}) and second order (k_1) rate constants for product formation measured at λ = 705 nm with 0.3 mM **2** and varying [Me_2NCN]. Formation traces were fit to either a single or biexponential equation.

−62 °C	[Me_2NCN] (M)	k_{obs} (s^{-1})	k_1 ($\times 10^3$ M^{-1} s^{-1})
	0.001	3.24	
	0.002	5.04	3.72 ± 0.24
	0.005	15.1	
	0.010	36.1	
−54 °C	[Me_2NCN] (M)	k_{obs} (s^{-1})	k_1 ($\times 10^3$ M^{-1} s^{-1})
	0.001	5.21	
	0.002	8.26	6.76 ± 0.56
	0.005	25.1	
	0.010	65	
−49 °C	[Me_2NCN] (M)	k_{obs} (s^{-1})	k_1 ($\times 10^3$ M^{-1} s^{-1})
	0.001	7.72	
	0.002	13.3	8.71 ± 0.42
	0.005	45	
	0.010	84	
−44 °C	[Me_2NCN] (M)	k_{obs} (s^{-1})	k_1 ($\times 10^3$ M^{-1} s^{-1})
	0.001	11.5	
	0.002	22.2	11.7 ± 0.7
	0.005	66	
	0.010	116	

Table S8. Temperature dependent pseudo-first order (k_{obs}) and second order (k_1) rate constants for product formation measured at λ = 687 nm with 0.3 mM **2** and varying [AdCN]. Formation traces were fit to a single exponential equation in all cases.

−62 °C	[AdCN] (M)	k_{obs} (s^{-1})	k_1 ($\times 10^3$ M^{-1} s^{-1})
	0.001	1.57	
	0.002	2.52	0.919 ± 0.015
	0.005	5.42	
	0.010	9.84	
−53 °C	[AdCN] (M)	k_{obs} (s^{-1})	k_1 ($\times 10^3$ M^{-1} s^{-1})
	0.001	2.68	
	0.002	4.92	2.52 ± 0.06
	0.005	12.0	
	0.010	25.3	
−44 °C	[AdCN] (M)	k_{obs} (s^{-1})	k_1 ($\times 10^3$ M^{-1} s^{-1})
	0.001	5.17	
	0.002	9.59	3.86 ± 0.11
	0.005	22.0	
	0.010	40.0	
−35 °C	[AdCN] (M)	k_{obs} (s^{-1})	k_1 ($\times 10^3$ M^{-1} s^{-1})
	0.001	8.09	
	0.002	14.9	6.71 ± 0.02
	0.005	35.2	
	0.010	68.5	

Table S9. Temperature dependent pseudo-first order (k_{obs}) and second order (k_1) rate constants for product formation measured at λ = 673 nm with 0.3 mM **2** and varying [AdNC]. Formation traces were fit to a biexponential equation at −62 °C with only k_{1obs} showing a dependence on [AdNC]. Formation traces at all remaining temperatures fit well to a single exponential equation.

−62 °C	[AdNC] (M)	k_{obs} (s^{-1})	k_1 ($\times 10^3$ M^{-1} s^{-1})
	0.001	3.60	3.08 ± 0.09
	0.002	5.93	
	0.005	14.8	
	0.010	31.1	
−53 °C	[AdNC] (M)	k_{obs} (s^{-1})	k_1 ($\times 10^3$ M^{-1} s^{-1})
	0.001	5.38	5.12 ± 0.05
	0.002	9.97	
	0.005	25.2	
	0.010	51.3	
−44 °C	[AdNC] (M)	k_{obs} (s^{-1})	k_1 ($\times 10^3$ M^{-1} s^{-1})
	0.001	9.61	8.58 ± 0.19
	0.002	18.8	
	0.005	46.3	
	0.010	86.9	
−35 °C	[AdNC] (M)	k_{obs} (s^{-1})	k_1 ($\times 10^3$ M^{-1} s^{-1})
	0.001	16.7	11.8 ± 0.7
	0.002	33.2	
	0.005	72.4	
	0.010	124	

Table S10. Temperature dependent pseudo-first order (k_{obs}) and second order (k_1) rate constants for adduct formation measured at λ = 550 nm with 0.15 mM **1** and varying [DFBN].

−40 °C	[DFBN] (M)	k_{obs} (s^{-1})	k_1 ($\times10^3$ M^{-1} s^{-1})
	0.00075	0.30	
	0.0015	0.58	
	0.006	1.99	0.316 ± 0.003
	0.01	3.30	
	0.015	4.80	
−20 °C	[DFBN] (M)	k_{obs} (s^{-1})	k_1 ($\times10^3$ M^{-1} s^{-1})
	0.00075	0.66	
	0.0015	1.22	
	0.006	4.54	0.750 ± 0.007
	0.01	7.45	
	0.015	11.4	
0 °C	[DFBN] (M)	k_{obs} (s^{-1})	k_1 ($\times10^3$ M^{-1} s^{-1})
	0.00075	1.48	
	0.0015	2.84	
	0.006	9.29	1.93 ± 0.13
	0.01	20.6	
	0.015	28.2	
+20 °C	[DFBN] (M)	k_{obs} (s^{-1})	k_1 ($\times10^3$ M^{-1} s^{-1})
	0.00075	3.32	
	0.0015	5.49	2.95 ± 0.24
	0.006	16.2	
	0.01	31.3	

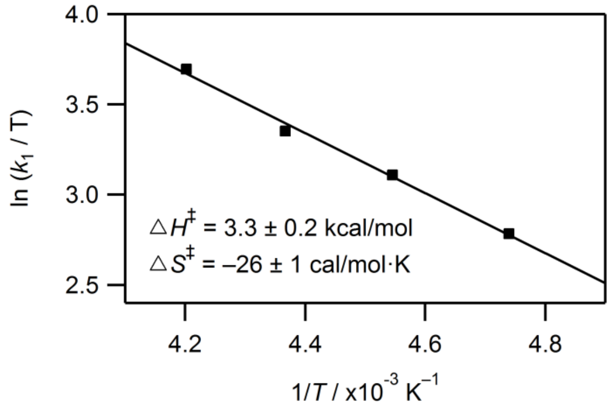

Figure S10. Eyring plot for DFBN binding to **2** (0.3 mM) over a temperature range of −62 °C to −35 °C with derived activation parameters.

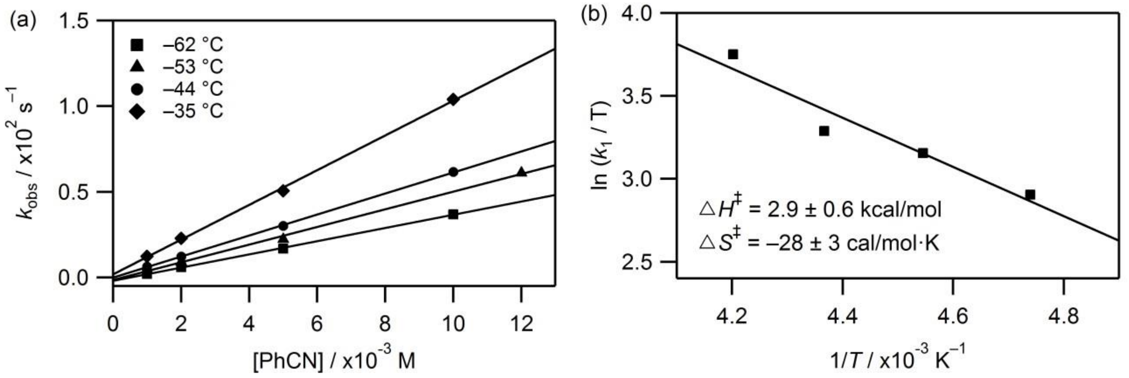

Figure S11. (a) Temperature dependent plots of k_{obs} versus [PhCN] for **2**. (b) Eyring plot for PhCN binding to **2** (0.3 mM) over a temperature range of −62 °C to −35 °C with derived activation parameters.

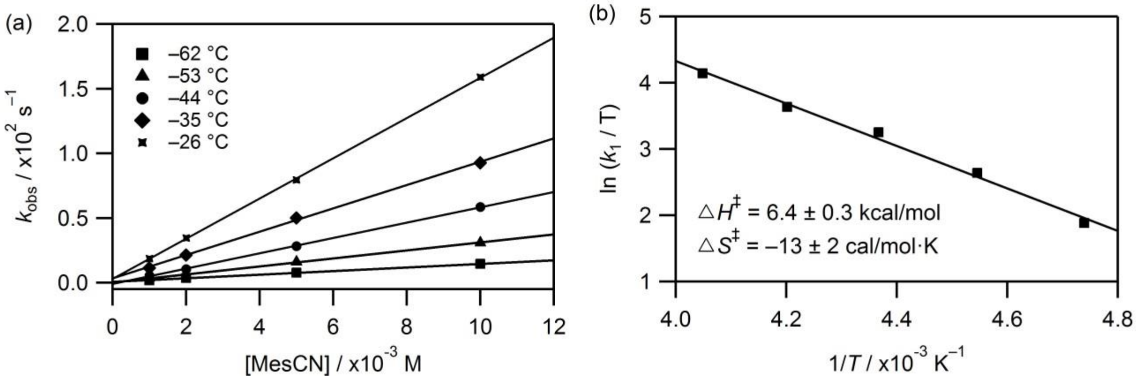

Figure S12. (a) Temperature dependent plots of k_{obs} versus [MesCN] for **2**. (b) Eyring plot for MesCN binding to **2** (0.3 mM) over a temperature range of −62 °C to −26 °C with derived activation parameters.

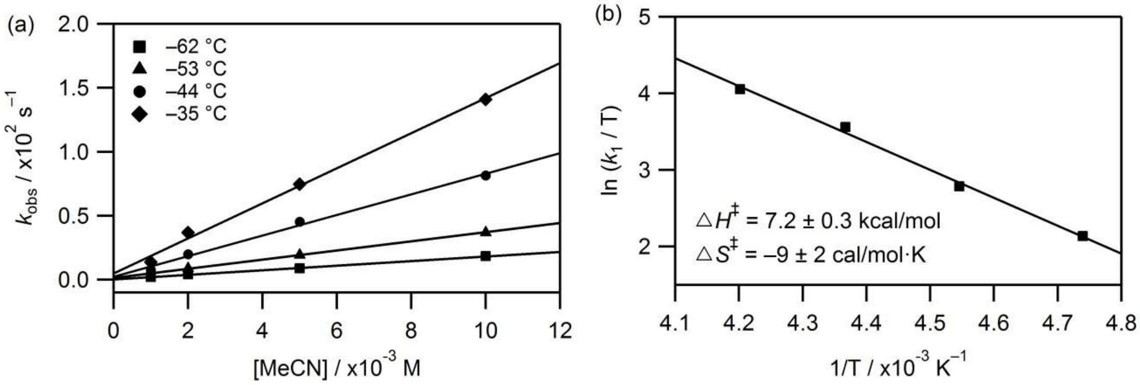

Figure S13. (a) Temperature dependent plots of k_{obs} versus [MeCN] for **2**. (b) Eyring plot for MeCN binding to **2** (0.3 mM) over a temperature range of −62 °C to −35 °C with derived activation parameters.

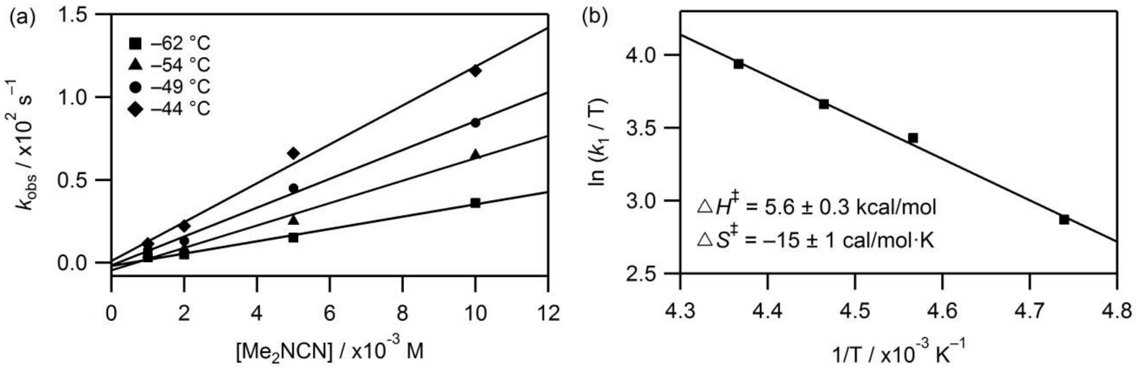

Figure S14. (a) Temperature dependent plots of k_{obs} versus [Me_2NCN] for **2**. (b) Eyring plot for Me_2NCN binding to **2** (0.3 mM) over a temperature range of −62 °C to −44 °C with derived activation parameters.

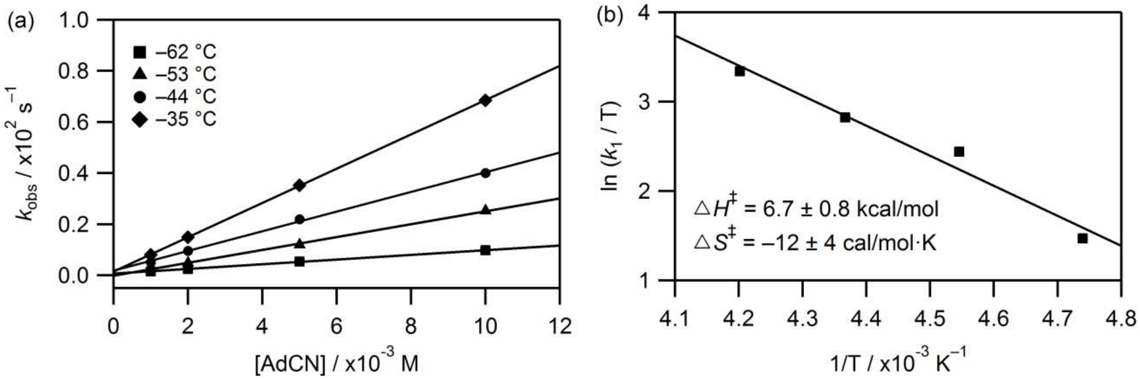

Figure S15. (a) Temperature dependent plots of k_{obs} versus [AdCN] for **2**. (b) Eyring plot for AdCN binding to **2** (0.3 mM) over a temperature range of −62 °C to −35 °C with derived activation parameters.

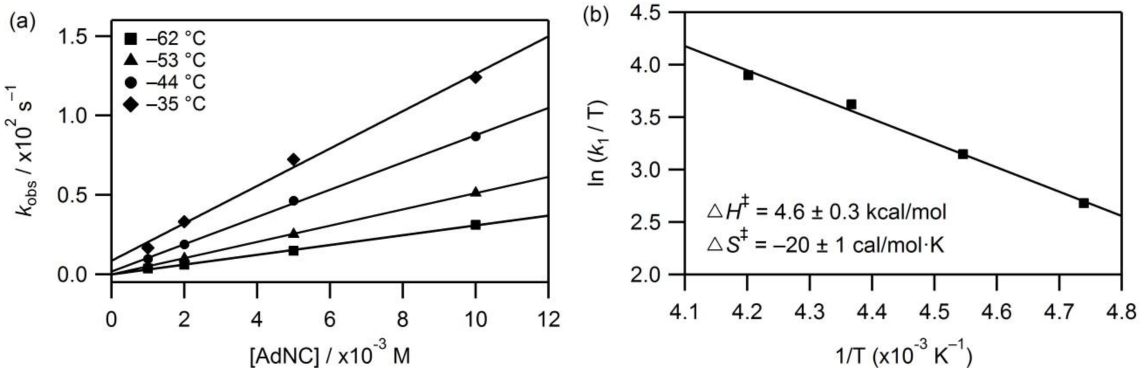

Figure S16. (a) Temperature dependent plots of k_{obs} versus [AdNC] for **2**. (b) Eyring plot for AdNC binding to **2** (0.3 mM) over a temperature range of −62 °C to −35 °C with derived activation parameters.

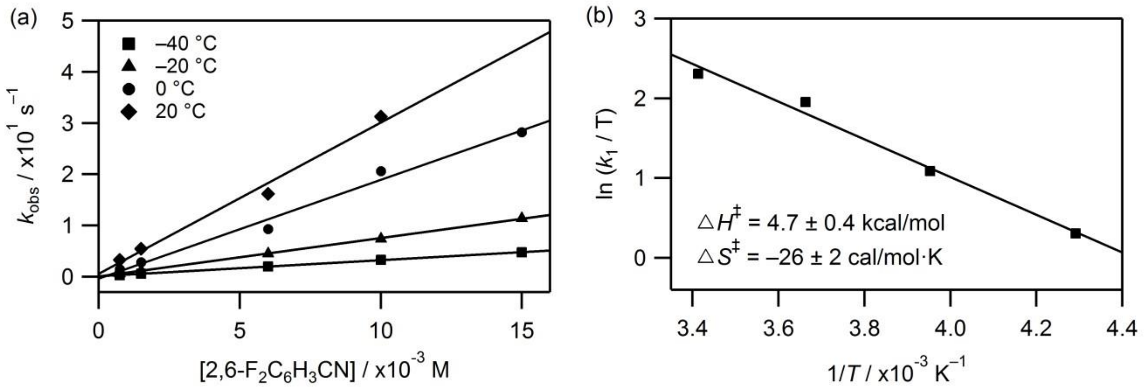

Figure S17. (a) Temperature dependent plots of k_{obs} versus [DFBN] for **1**; (b) Eyring plot for DFBN binding to **1** (0.15 mM) over a temperature range of −40 °C to +20 °C with derived activation parameters.

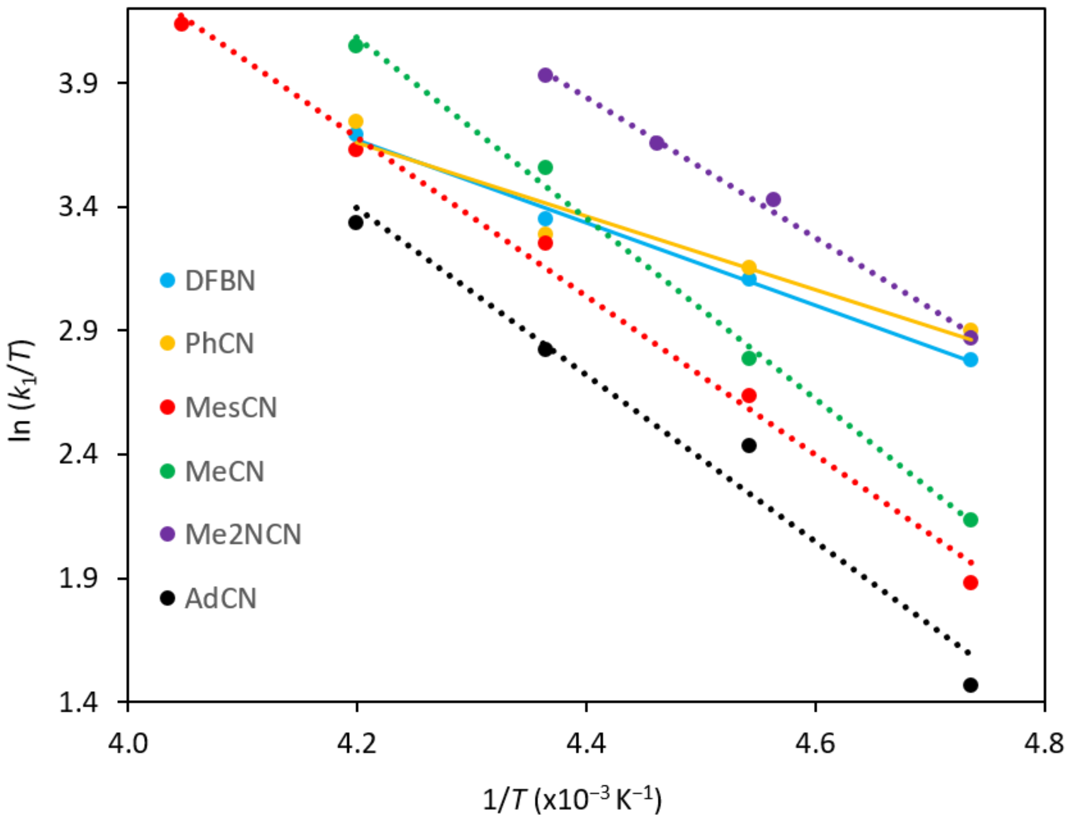

Figure S18. Eyring plots for binding of RCN to **2** displaying markedly different slopes for binding of PhCN and DFBN (solid lines) compared to other nitrile ligands (dotted lines). This clear difference is outside anticipated experimental errors.

X-ray Crystallography. Low-temperature diffraction data were collected on a Siemens Platform three-circle diffractometer equipped with a Bruker APEX CCD, using graphite

monochromated Mo K_α radiation (λ = 0.71073 Å) from a water-cooled sealed tube. The structures were solved by dual-space methods using SHELXT[9] and refined against F^2 on all data by full-matrix least squares with SHELXL-2017[10] following established refinement strategies.[11] All non-hydrogen atoms were refined anisotropically. All hydrogen atoms were included into the model at geometrically calculated positions and refined using a riding model. The isotropic displacement parameters of all hydrogen atoms were fixed to 1.2 times the *U*-value of the atoms they are linked to (1.5 times for methyl groups).

Compound DFBN-**2** crystallizes in the triclinic centrosymmetric space group *P*-1 with one molecule of DFBN-**2** per asymmetric unit. Structure determination was straightforward and no restraints were applied.

Compound Me_2NCN-**2** crystallizes in the cubic centrosymmetric space group *Pa*-3 with one third molecule of Me_2NCN-**2** per asymmetric unit. The triple-bonded NMe_2 moiety was refined as six-fold disordered in a fashion where two disorder components are crystallographically independent, the other four are generated by the same crystallographic threefold axis that completes the full molecule from the third contained in the asymmetric unit (see Figure S19). The ratio of the two independent disorder components was refined freely and converged at 0.50(4). Thus, each disorder component is occupied to approximately one sixth. This makes discussing the geometry of the NMe_2 moiety difficult; especially the C—N—C angles cannot be determined with confidence. The disorder was refined with the help of similarity restraints on 1-2 and 1-3 distances and displacement parameters as well as rigid bond restraints for anisotropic displacement parameters.

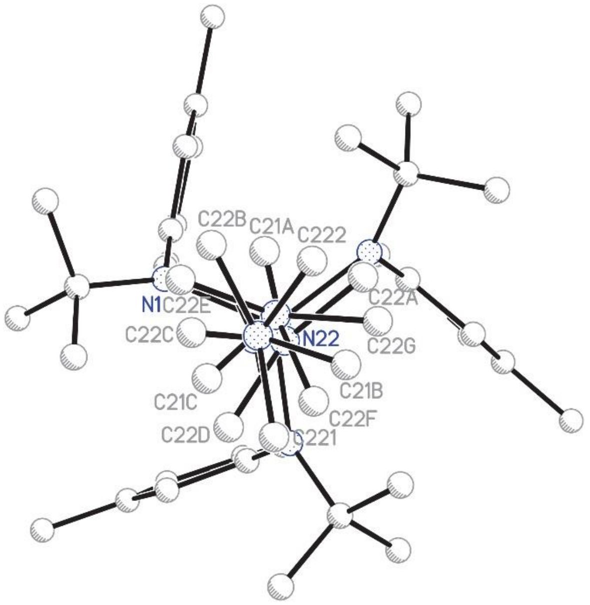

Figure S19. Plot showing the six-fold disorder present for the triple-bonded NMe_2 group in Me_2NCN-**2**.

Compound [PhCN-**2**][BAr^F_4] crystallizes in the triclinic centrosymmetric space group *P*-1 with one molecule of [PhCN-**2**][BAr^F_4], one BAr^F counter ion, and one diethyl ether molecule in the asymmetric unit. Several of the eight CF_3 groups of the BAr^F ion were found to be disordered. This could be modelled for four of them. Additionally, one of the three nitrogen bound dimethyl-phenyl rings was modelled as disordered over two positions. The disorders were refined with the help of similarity restraints on 1-2 and 1-3 distances and displacement parameters as well as rigid bond restraints for anisotropic displacement parameters. All disorder ratios were refined freely; for the dimethyl-phenyl ring it converged at 0.622(7).

Details of the data quality and a summary of the residual values of the refinement of DFBN-**2**, Me_2NCN-**2** and [PhCN-**2**][BAr^F_4] are listed in Tables S11-S13. Tables S14-S16 give all bond lengths and angles for the structures.

Table S11. Crystal data and structure refinement for DFBN-**2**.

Identification code	08265	
Empirical formula	$C_{43} H_{57} F_2 N_4 V$	
Formula weight	718.86	
Temperature	100(2) K	
Wavelength	0.71073 Å	
Crystal system	Triclinic	
Space group	*P*-1	
Unit cell dimensions	*a* = 10.3639(10) Å	α= 76.4565(16)°.
	b = 12.8153(12) Å	β= 81.7351(17)°.
	c = 16.2440(15) Å	γ = 69.8015(17)°.
Volume	1963.8(3) $Å^3$	
Z	2	
Density (calculated)	1.216 Mg/m^3	
Absorption coefficient	0.297 mm^{-1}	
F(000)	768	
Crystal size	0.300 x 0.090 x 0.090 mm^3	
Theta range for data collection	1.728 to 30.076°.	
Index ranges	-14<=*h*<=14, -18<=*k*<=17, -22<=*l*<=22	
Reflections collected	45404	
Independent reflections	11477 [R_{int} = 0.0735]	
Completeness to theta = 25.242°	99.9 %	
Absorption correction	Semi-empirical from equivalents	
Refinement method	Full-matrix least-squares on F^2	
Data / restraints / parameters	11477 / 0 / 466	
Goodness-of-fit on F^2	1.011	
Final *R* indices [$I>2\sigma(I)$]	*R*1 = 0.0505, *wR*2 = 0.1150	
R indices (all data)	*R*1 = 0.0882, *wR*2 = 0.1347	
Largest diff. peak and hole	0.563 and -0.534 e.$Å^{-3}$	

Table S12. Crystal data and structure refinement for Me_2NCN-**2**.

Identification code	10214	
Empirical formula	$C_{39} H_{60} N_5 V$	
Formula weight	649.86	
Temperature	100(2) K	
Wavelength	0.71073 Å	
Crystal system	Cubic	
Space group	*Pa*-3	
Unit cell dimensions	*a* = 19.6998(12) Å	α = 90°.
	b = 19.6998(12) Å	β = 90°.
	c = 19.6998(12) Å	γ = 90°.
Volume	7645.1(13) $Å^3$	
Z	8	
Density (calculated)	1.129 Mg/m^3	
Absorption coefficient	0.291 mm^{-1}	
F(000)	2816	
Crystal size	0.150 x 0.150 x 0.150 mm^3	
Theta range for data collection	1.790 to 29.562°.	
Index ranges	-27<=*h*<=27, -27<=*k*<=27, -27<=*l*<=27	
Reflections collected	170070	
Independent reflections	3582 [R_{int} = 0.0811]	
Completeness to theta = 25.242°	100.0 %	
Absorption correction	Semi-empirical from equivalents	
Max. and min. transmission	0.7460 and 0.6522	
Refinement method	Full-matrix least-squares on F^2	
Data / restraints / parameters	3582 / 135 / 181	
Goodness-of-fit on F^2	1.022	
Final *R* indices [I>2σ(I)]	*R*1 = 0.0529, *wR*2 = 0.1375	
R indices (all data)	*R*1 = 0.0760, *wR*2 = 0.1593	
Largest diff. peak and hole	0.711 and -0.398 e.$Å^{-3}$	

Table S13. Crystal data and structure refinement for [PhCN-**2**][$BAr^F{}_4$].

Identification code	10301	
Empirical formula	$C_{79} H_{81} B F_{24} N_4 O V$	
Formula weight	1620.22	
Temperature	100(2) K	
Wavelength	0.71073 Å	
Crystal system	Triclinic	
Space group	*P*-1	
Unit cell dimensions	*a* = 12.5771(6) Å	α = 89.8410(8)°.
	b = 17.8763(9) Å	β = 70.6514(8)°.
	c = 18.8513(9) Å	γ = 84.2776(8)°.
Volume	3977.0(3) $Å^3$	
Z	2	
Density (calculated)	1.353 Mg/m^3	
Absorption coefficient	0.227 mm^{-1}	
F(000)	1670	
Crystal size	0.300 x 0.250 x 0.100 mm^3	
Theta range for data collection	1.726 to 29.575°.	
Index ranges	-17<=*h*<=17, -24<=*k*<=24, -26<=*l*<=26	
Reflections collected	90072	
Independent reflections	22161 [R_{int} = 0.0542]	
Completeness to theta = 25.242°	100.0 %	
Absorption correction	Semi-empirical from equivalents	
Max. and min. transmission	0.7459 and 0.6521	
Refinement method	Full-matrix least-squares on F^2	
Data / restraints / parameters	22161 / 3687 / 1145	
Goodness-of-fit on F^2	1.042	
Final *R* indices [I>2σ(I)]	*R*1 = 0.0507, *wR*2 = 0.1092	
R indices (all data)	*R*1 = 0.0874, *wR*2 = 0.1281	
Largest diff. peak and hole	0.485 and -0.544 e.$Å^{-3}$	

Table S14. Bond lengths [Å] and angles [°] for DFBN-**2**.

V(1)-N(2)	1.9412(15)	C(231)-H(23A)	0.9800
V(1)-N(3)	1.9416(16)	C(231)-H(23B)	0.9800
V(1)-N(1)	1.9439(15)	C(231)-H(23C)	0.9800
V(1)-N(4)	2.0417(16)	C(251)-H(25A)	0.9800
N(1)-C(11)	1.435(2)	C(251)-H(25B)	0.9800
N(1)-C(17)	1.495(2)	C(251)-H(25C)	0.9800
N(2)-C(21)	1.436(2)	C(271)-H(27A)	0.9800
N(2)-C(27)	1.491(2)	C(271)-H(27B)	0.9800
N(3)-C(31)	1.433(2)	C(271)-H(27C)	0.9800
N(3)-C(37)	1.493(2)	C(272)-H(27D)	0.9800
N(4)-C(41)	1.151(2)	C(272)-H(27E)	0.9800
C(11)-C(12)	1.398(3)	C(272)-H(27F)	0.9800
C(11)-C(16)	1.402(3)	C(273)-H(27G)	0.9800
C(12)-C(13)	1.398(3)	C(273)-H(27H)	0.9800
C(12)-H(12)	0.9500	C(273)-H(27I)	0.9800
C(13)-C(14)	1.390(3)	C(31)-C(32)	1.395(3)
C(13)-C(131)	1.511(3)	C(31)-C(36)	1.401(3)
C(14)-C(15)	1.398(3)	C(32)-C(33)	1.399(3)
C(14)-H(14)	0.9500	C(32)-H(32)	0.9500
C(15)-C(16)	1.389(3)	C(33)-C(34)	1.389(3)
C(15)-C(151)	1.512(3)	C(33)-C(331)	1.508(3)
C(16)-H(16)	0.9500	C(34)-C(35)	1.391(3)
C(17)-C(171)	1.533(3)	C(34)-H(34)	0.9500
C(17)-C(172)	1.536(3)	C(35)-C(36)	1.396(3)
C(17)-C(173)	1.539(3)	C(35)-C(351)	1.514(3)
C(131)-H(13A)	0.9800	C(36)-H(36)	0.9500
C(131)-H(13B)	0.9800	C(37)-C(372)	1.526(3)
C(131)-H(13C)	0.9800	C(37)-C(373)	1.537(3)
C(151)-H(15A)	0.9800	C(37)-C(371)	1.539(3)
C(151)-H(15B)	0.9800	C(331)-H(33A)	0.9800
C(151)-H(15C)	0.9800	C(331)-H(33B)	0.9800
C(171)-H(17A)	0.9800	C(331)-H(33C)	0.9800
C(171)-H(17B)	0.9800	C(351)-H(35A)	0.9800
C(171)-H(17C)	0.9800	C(351)-H(35B)	0.9800
C(172)-H(17D)	0.9800	C(351)-H(35C)	0.9800
C(172)-H(17E)	0.9800	C(371)-H(37A)	0.9800
C(172)-H(17F)	0.9800	C(371)-H(37B)	0.9800
C(173)-H(17G)	0.9800	C(371)-H(37C)	0.9800
C(173)-H(17H)	0.9800	C(372)-H(37D)	0.9800
C(173)-H(17I)	0.9800	C(372)-H(37E)	0.9800
C(21)-C(26)	1.399(3)	C(372)-H(37F)	0.9800
C(21)-C(22)	1.404(3)	C(373)-H(37G)	0.9800
C(22)-C(23)	1.399(3)	C(373)-H(37H)	0.9800
C(22)-H(22)	0.9500	C(373)-H(37I)	0.9800
C(23)-C(24)	1.396(3)	C(41)-C(42)	1.423(3)
C(23)-C(231)	1.514(3)	C(42)-C(43)	1.397(3)
C(24)-C(25)	1.391(3)	C(42)-C(47)	1.398(3)
C(24)-H(24)	0.9500	C(43)-F(431)	1.345(2)
C(25)-C(26)	1.396(3)	C(43)-C(44)	1.373(3)
C(25)-C(251)	1.514(3)	C(44)-C(45)	1.386(3)
C(26)-H(26)	0.9500	C(44)-H(44)	0.9500
C(27)-C(273)	1.526(3)	C(45)-C(46)	1.389(3)
C(27)-C(271)	1.530(3)	C(45)-H(45)	0.9500
C(27)-C(272)	1.536(3)	C(46)-C(47)	1.371(3)

C(46)-H(46)	0.9500
C(47)-F(471)	1.344(2)
N(2)-V(1)-N(3)	118.09(7)
N(2)-V(1)-N(1)	121.00(6)
N(3)-V(1)-N(1)	117.58(7)
N(2)-V(1)-N(4)	89.68(6)
N(3)-V(1)-N(4)	98.71(6)
N(1)-V(1)-N(4)	99.89(6)
C(11)-N(1)-C(17)	114.67(14)
C(11)-N(1)-V(1)	113.58(11)
C(17)-N(1)-V(1)	130.96(11)
C(21)-N(2)-C(27)	113.53(14)
C(21)-N(2)-V(1)	124.86(12)
C(27)-N(2)-V(1)	121.36(12)
C(31)-N(3)-C(37)	113.93(14)
C(31)-N(3)-V(1)	114.29(12)
C(37)-N(3)-V(1)	131.51(12)
C(41)-N(4)-V(1)	161.79(15)
C(12)-C(11)-C(16)	117.85(17)
C(12)-C(11)-N(1)	120.27(16)
C(16)-C(11)-N(1)	121.78(16)
C(13)-C(12)-C(11)	121.62(18)
C(13)-C(12)-H(12)	119.2
C(11)-C(12)-H(12)	119.2
C(14)-C(13)-C(12)	118.70(18)
C(14)-C(13)-C(131)	121.29(18)
C(12)-C(13)-C(131)	119.97(18)
C(13)-C(14)-C(15)	121.26(17)
C(13)-C(14)-H(14)	119.4
C(15)-C(14)-H(14)	119.4
C(16)-C(15)-C(14)	118.74(17)
C(16)-C(15)-C(151)	120.75(17)
C(14)-C(15)-C(151)	120.49(17)
C(15)-C(16)-C(11)	121.74(17)
C(15)-C(16)-H(16)	119.1
C(11)-C(16)-H(16)	119.1
N(1)-C(17)-C(171)	108.14(15)
N(1)-C(17)-C(172)	111.39(15)
C(171)-C(17)-C(172)	107.42(15)
N(1)-C(17)-C(173)	111.19(15)
C(171)-C(17)-C(173)	109.92(15)
C(172)-C(17)-C(173)	108.70(16)
C(13)-C(131)-H(13A)	109.5
C(13)-C(131)-H(13B)	109.5
H(13A)-C(131)-H(13B)	109.5
C(13)-C(131)-H(13C)	109.5
H(13A)-C(131)-H(13C)	109.5
H(13B)-C(131)-H(13C)	109.5
C(15)-C(151)-H(15A)	109.5
C(15)-C(151)-H(15B)	109.5
H(15A)-C(151)-H(15B)	109.5
C(15)-C(151)-H(15C)	109.5
H(15A)-C(151)-H(15C)	109.5
H(15B)-C(151)-H(15C)	109.5
C(17)-C(171)-H(17A)	109.5

C(17)-C(171)-H(17B)	109.5
H(17A)-C(171)-H(17B)	109.5
C(17)-C(171)-H(17C)	109.5
H(17A)-C(171)-H(17C)	109.5
H(17B)-C(171)-H(17C)	109.5
C(17)-C(172)-H(17D)	109.5
C(17)-C(172)-H(17E)	109.5
H(17D)-C(172)-H(17E)	109.5
C(17)-C(172)-H(17F)	109.5
H(17D)-C(172)-H(17F)	109.5
H(17E)-C(172)-H(17F)	109.5
C(17)-C(173)-H(17G)	109.5
C(17)-C(173)-H(17H)	109.5
H(17G)-C(173)-H(17H)	109.5
C(17)-C(173)-H(17I)	109.5
H(17G)-C(173)-H(17I)	109.5
H(17H)-C(173)-H(17I)	109.5
C(26)-C(21)-C(22)	117.72(17)
C(26)-C(21)-N(2)	120.45(16)
C(22)-C(21)-N(2)	121.76(16)
C(23)-C(22)-C(21)	121.20(18)
C(23)-C(22)-H(22)	119.4
C(21)-C(22)-H(22)	119.4
C(24)-C(23)-C(22)	119.10(18)
C(24)-C(23)-C(231)	119.67(17)
C(22)-C(23)-C(231)	121.23(18)
C(25)-C(24)-C(23)	121.28(18)
C(25)-C(24)-H(24)	119.4
C(23)-C(24)-H(24)	119.4
C(24)-C(25)-C(26)	118.40(18)
C(24)-C(25)-C(251)	121.45(18)
C(26)-C(25)-C(251)	120.13(18)
C(25)-C(26)-C(21)	122.29(18)
C(25)-C(26)-H(26)	118.9
C(21)-C(26)-H(26)	118.9
N(2)-C(27)-C(273)	112.38(15)
N(2)-C(27)-C(271)	107.19(15)
C(273)-C(27)-C(271)	107.79(16)
N(2)-C(27)-C(272)	111.13(15)
C(273)-C(27)-C(272)	109.12(15)
C(271)-C(27)-C(272)	109.11(16)
C(23)-C(231)-H(23A)	109.5
C(23)-C(231)-H(23B)	109.5
H(23A)-C(231)-H(23B)	109.5
C(23)-C(231)-H(23C)	109.5
H(23A)-C(231)-H(23C)	109.5
H(23B)-C(231)-H(23C)	109.5
C(25)-C(251)-H(25A)	109.5
C(25)-C(251)-H(25B)	109.5
H(25A)-C(251)-H(25B)	109.5
C(25)-C(251)-H(25C)	109.5
H(25A)-C(251)-H(25C)	109.5
H(25B)-C(251)-H(25C)	109.5
C(27)-C(271)-H(27A)	109.5
C(27)-C(271)-H(27B)	109.5
H(27A)-C(271)-H(27B)	109.5

C(27)-C(271)-H(27C)	109.5
H(27A)-C(271)-H(27C)	109.5
H(27B)-C(271)-H(27C)	109.5
C(27)-C(272)-H(27D)	109.5
C(27)-C(272)-H(27E)	109.5
H(27D)-C(272)-H(27E)	109.5
C(27)-C(272)-H(27F)	109.5
H(27D)-C(272)-H(27F)	109.5
H(27E)-C(272)-H(27F)	109.5
C(27)-C(273)-H(27G)	109.5
C(27)-C(273)-H(27H)	109.5
H(27G)-C(273)-H(27H)	109.5
C(27)-C(273)-H(27I)	109.5
H(27G)-C(273)-H(27I)	109.5
H(27H)-C(273)-H(27I)	109.5
C(32)-C(31)-C(36)	117.66(17)
C(32)-C(31)-N(3)	120.82(17)
C(36)-C(31)-N(3)	121.49(17)
C(31)-C(32)-C(33)	122.13(18)
C(31)-C(32)-H(32)	118.9
C(33)-C(32)-H(32)	118.9
C(34)-C(33)-C(32)	118.23(18)
C(34)-C(33)-C(331)	121.49(18)
C(32)-C(33)-C(331)	120.28(18)
C(33)-C(34)-C(35)	121.68(18)
C(33)-C(34)-H(34)	119.2
C(35)-C(34)-H(34)	119.2
C(34)-C(35)-C(36)	118.63(18)
C(34)-C(35)-C(351)	120.68(17)
C(36)-C(35)-C(351)	120.68(18)
C(35)-C(36)-C(31)	121.67(18)
C(35)-C(36)-H(36)	119.2
C(31)-C(36)-H(36)	119.2
N(3)-C(37)-C(372)	108.82(15)
N(3)-C(37)-C(373)	111.90(16)
C(372)-C(37)-C(373)	109.31(16)
N(3)-C(37)-C(371)	110.53(16)
C(372)-C(37)-C(371)	108.21(16)
C(373)-C(37)-C(371)	107.99(17)
C(33)-C(331)-H(33A)	109.5
C(33)-C(331)-H(33B)	109.5
H(33A)-C(331)-H(33B)	109.5
C(33)-C(331)-H(33C)	109.5
H(33A)-C(331)-H(33C)	109.5
H(33B)-C(331)-H(33C)	109.5
C(35)-C(351)-H(35A)	109.5
C(35)-C(351)-H(35B)	109.5
H(35A)-C(351)-H(35B)	109.5
C(35)-C(351)-H(35C)	109.5
H(35A)-C(351)-H(35C)	109.5
H(35B)-C(351)-H(35C)	109.5
C(37)-C(371)-H(37A)	109.5
C(37)-C(371)-H(37B)	109.5
H(37A)-C(371)-H(37B)	109.5
C(37)-C(371)-H(37C)	109.5
H(37A)-C(371)-H(37C)	109.5
H(37B)-C(371)-H(37C)	109.5
C(37)-C(372)-H(37D)	109.5
C(37)-C(372)-H(37E)	109.5
H(37D)-C(372)-H(37E)	109.5
C(37)-C(372)-H(37F)	109.5
H(37D)-C(372)-H(37F)	109.5
H(37E)-C(372)-H(37F)	109.5
C(37)-C(373)-H(37G)	109.5
C(37)-C(373)-H(37H)	109.5
H(37G)-C(373)-H(37H)	109.5
C(37)-C(373)-H(37I)	109.5
H(37G)-C(373)-H(37I)	109.5
H(37H)-C(373)-H(37I)	109.5
N(4)-C(41)-C(42)	175.0(2)
C(43)-C(42)-C(47)	116.53(17)
C(43)-C(42)-C(41)	121.06(17)
C(47)-C(42)-C(41)	122.29(17)
F(431)-C(43)-C(44)	120.01(17)
F(431)-C(43)-C(42)	117.52(17)
C(44)-C(43)-C(42)	122.47(18)
C(43)-C(44)-C(45)	118.57(19)
C(43)-C(44)-H(44)	120.7
C(45)-C(44)-H(44)	120.7
C(44)-C(45)-C(46)	121.38(18)
C(44)-C(45)-H(45)	119.3
C(46)-C(45)-H(45)	119.3
C(47)-C(46)-C(45)	118.26(18)
C(47)-C(46)-H(46)	120.9
C(45)-C(46)-H(46)	120.9
F(471)-C(47)-C(46)	120.22(17)
F(471)-C(47)-C(42)	117.01(16)
C(46)-C(47)-C(42)	122.77(18)

Table S15. Bond lengths [Å] and angles [°] for Me_2NCN-**2**.

V(1)-N(1)#1	1.9351(15)		
V(1)-N(1)#2	1.9351(15)	N(1)#1-V(1)-N(1)#2	116.49(3)
V(1)-N(1)	1.9352(15)	N(1)#1-V(1)-N(1)	116.49(3)
V(1)-N(2)	2.038(3)	N(1)#2-V(1)-N(1)	116.49(3)
N(1)-C(11)	1.437(2)	N(1)#1-V(1)-N(2)	100.93(5)
N(1)-C(17)	1.490(2)	N(1)#2-V(1)-N(2)	100.93(5)
C(11)-C(16)	1.400(3)	N(1)-V(1)-N(2)	100.94(5)
C(11)-C(12)	1.401(2)	C(11)-N(1)-C(17)	115.62(15)
C(12)-C(13)	1.397(3)	C(11)-N(1)-V(1)	110.28(11)
C(12)-H(12)	0.9500	C(17)-N(1)-V(1)	134.06(13)
C(13)-C(14)	1.397(3)	C(16)-C(11)-C(12)	118.00(18)
C(13)-C(131)	1.508(3)	C(16)-C(11)-N(1)	121.21(16)
C(14)-C(15)	1.393(3)	C(12)-C(11)-N(1)	120.72(16)
C(14)-H(14)	0.9500	C(13)-C(12)-C(11)	121.47(18)
C(15)-C(16)	1.394(3)	C(13)-C(12)-H(12)	119.3
C(15)-C(151)	1.518(3)	C(11)-C(12)-H(12)	119.3
C(16)-H(16)	0.9500	C(12)-C(13)-C(14)	118.82(18)
C(17)-C(172)	1.520(3)	C(12)-C(13)-C(131)	120.82(18)
C(17)-C(171)	1.526(3)	C(14)-C(13)-C(131)	120.36(19)
C(17)-C(173)	1.550(3)	C(15)-C(14)-C(13)	121.1(2)
C(131)-H(13A)	0.9800	C(15)-C(14)-H(14)	119.5
C(131)-H(13B)	0.9800	C(13)-C(14)-H(14)	119.5
C(131)-H(13C)	0.9800	C(14)-C(15)-C(16)	118.87(19)
C(151)-H(15A)	0.9800	C(14)-C(15)-C(151)	120.0(2)
C(151)-H(15B)	0.9800	C(16)-C(15)-C(151)	121.1(2)
C(151)-H(15C)	0.9800	C(15)-C(16)-C(11)	121.69(18)
C(171)-H(17A)	0.9800	C(15)-C(16)-H(16)	119.2
C(171)-H(17B)	0.9800	C(11)-C(16)-H(16)	119.2
C(171)-H(17C)	0.9800	N(1)-C(17)-C(172)	110.95(19)
C(172)-H(17D)	0.9800	N(1)-C(17)-C(171)	110.20(17)
C(172)-H(17E)	0.9800	C(172)-C(17)-C(171)	108.27(19)
C(172)-H(17F)	0.9800	N(1)-C(17)-C(173)	110.46(17)
C(173)-H(17G)	0.9800	C(172)-C(17)-C(173)	111.1(2)
C(173)-H(17H)	0.9800	C(171)-C(17)-C(173)	105.7(2)
C(173)-H(17I)	0.9800	C(13)-C(131)-H(13A)	109.5
N(2)-C(21)	1.149(4)	C(13)-C(131)-H(13B)	109.5
C(21)-N(22)	1.329(10)	H(13A)-C(131)-H(13B)	109.5
C(21)-N(22B)	1.332(11)	C(13)-C(131)-H(13C)	109.5
N(22)-C(221)	1.517(16)	H(13A)-C(131)-H(13C)	109.5
N(22)-C(222)	1.520(14)	H(13B)-C(131)-H(13C)	109.5
C(221)-H(22A)	0.9800	C(15)-C(151)-H(15A)	109.5
C(221)-H(22B)	0.9800	C(15)-C(151)-H(15B)	109.5
C(221)-H(22C)	0.9800	H(15A)-C(151)-H(15B)	109.5
C(222)-H(22D)	0.9800	C(15)-C(151)-H(15C)	109.5
C(222)-H(22E)	0.9800	H(15A)-C(151)-H(15C)	109.5
C(222)-H(22F)	0.9800	H(15B)-C(151)-H(15C)	109.5
N(22B)-C(21B)	1.520(14)	C(17)-C(171)-H(17A)	109.5
N(22B)-C(22B)	1.524(15)	C(17)-C(171)-H(17B)	109.5
C(21B)-H(21A)	0.9800	H(17A)-C(171)-H(17B)	109.5
C(21B)-H(21B)	0.9800	C(17)-C(171)-H(17C)	109.5
C(21B)-H(21C)	0.9800	H(17A)-C(171)-H(17C)	109.5
C(22B)-H(22G)	0.9800	H(17B)-C(171)-H(17C)	109.5
C(22B)-H(22H)	0.9800	C(17)-C(172)-H(17D)	109.5
C(22B)-H(22I)	0.9800	C(17)-C(172)-H(17E)	109.5

H(17D)-C(172)-H(17E)	109.5
C(17)-C(172)-H(17F)	109.5
H(17D)-C(172)-H(17F)	109.5
H(17E)-C(172)-H(17F)	109.5
C(17)-C(173)-H(17G)	109.5
C(17)-C(173)-H(17H)	109.5
H(17G)-C(173)-H(17H)	109.5
C(17)-C(173)-H(17I)	109.5
H(17G)-C(173)-H(17I)	109.5
H(17H)-C(173)-H(17I)	109.5
C(21)-N(2)-V(1)	180.0(4)
N(2)-C(21)-N(22)	170.6(12)
N(2)-C(21)-N(22B)	168.0(11)
C(21)-N(22)-C(221)	116.7(16)
C(21)-N(22)-C(222)	116.4(14)
C(221)-N(22)-C(222)	106.5(11)
N(22)-C(221)-H(22A)	109.5
N(22)-C(221)-H(22B)	109.5
H(22A)-C(221)-H(22B)	109.5
N(22)-C(221)-H(22C)	109.5
H(22A)-C(221)-H(22C)	109.5
H(22B)-C(221)-H(22C)	109.5
N(22)-C(222)-H(22D)	109.5
N(22)-C(222)-H(22E)	109.5
H(22D)-C(222)-H(22E)	109.5
N(22)-C(222)-H(22F)	109.5
H(22D)-C(222)-H(22F)	109.5
H(22E)-C(222)-H(22F)	109.5
C(21)-N(22B)-C(21B)	113.0(15)
C(21)-N(22B)-C(22B)	112.3(14)
C(21B)-N(22B)-C(22B)	106.3(11)
N(22B)-C(21B)-H(21A)	109.5
N(22B)-C(21B)-H(21B)	109.5
H(21A)-C(21B)-H(21B)	109.5
N(22B)-C(21B)-H(21C)	109.5
H(21A)-C(21B)-H(21C)	109.5
H(21B)-C(21B)-H(21C)	109.5
N(22B)-C(22B)-H(22G)	109.5
N(22B)-C(22B)-H(22H)	109.5
H(22G)-C(22B)-H(22H)	109.5
N(22B)-C(22B)-H(22I)	109.5
H(22G)-C(22B)-H(22I)	109.5
H(22H)-C(22B)-H(22I)	109.5

Symmetry transformations used to generate equivalent atoms:
#1 -y+3/2,-z+1,x-1/2 #2 z+1/2,-x+3/2,-y+1

Table S16. Bond lengths [Å] and angles [°] for [PhCN-**2**][BAr$^F{}_4$].

V(1)-N(1)	1.8572(14)	C(27)-C(272)	1.525(2)
V(1)-N(2)	1.8758(14)	C(27)-C(273)	1.529(3)
V(1)-N(3)	1.8915(15)	C(27)-C(271)	1.537(3)
V(1)-N(4)	2.0598(15)	C(271)-H(27A)	0.9800
N(1)-C(11)	1.444(2)	C(271)-H(27B)	0.9800
N(1)-C(17)	1.518(2)	C(271)-H(27C)	0.9800
C(11)-C(16)	1.392(2)	C(272)-H(27D)	0.9800
C(11)-C(12)	1.395(2)	C(272)-H(27E)	0.9800
C(12)-C(13)	1.392(2)	C(272)-H(27F)	0.9800
C(12)-H(12)	0.9500	C(273)-H(27G)	0.9800
C(13)-C(14)	1.395(3)	C(273)-H(27H)	0.9800
C(13)-C(131)	1.509(3)	C(273)-H(27I)	0.9800
C(131)-H(13A)	0.9800	N(3)-C(31)	1.453(4)
C(131)-H(13B)	0.9800	N(3)-C(31A)	1.453(5)
C(131)-H(13C)	0.9800	N(3)-C(37)	1.502(2)
C(14)-C(15)	1.392(3)	C(31)-C(32)	1.396(4)
C(14)-H(14)	0.9500	C(31)-C(36)	1.399(4)
C(15)-C(16)	1.398(2)	C(32)-C(33)	1.402(4)
C(15)-C(151)	1.507(3)	C(32)-H(32)	0.9500
C(151)-H(15A)	0.9800	C(33)-C(34)	1.393(5)
C(151)-H(15B)	0.9800	C(33)-C(331)	1.517(4)
C(151)-H(15C)	0.9800	C(331)-H(33A)	0.9800
C(16)-H(16)	0.9500	C(331)-H(33B)	0.9800
C(17)-C(172)	1.527(2)	C(331)-H(33C)	0.9800
C(17)-C(173)	1.531(2)	C(34)-C(35)	1.384(5)
C(17)-C(171)	1.531(2)	C(34)-H(34)	0.9500
C(171)-H(17A)	0.9800	C(35)-C(36)	1.396(4)
C(171)-H(17B)	0.9800	C(35)-C(351)	1.515(5)
C(171)-H(17C)	0.9800	C(351)-H(35A)	0.9800
C(172)-H(17D)	0.9800	C(351)-H(35B)	0.9800
C(172)-H(17E)	0.9800	C(351)-H(35C)	0.9800
C(172)-H(17F)	0.9800	C(36)-H(36)	0.9500
C(173)-H(17G)	0.9800	C(37)-C(372)	1.523(3)
C(173)-H(17H)	0.9800	C(37)-C(371)	1.532(3)
C(173)-H(17I)	0.9800	C(37)-C(373)	1.534(3)
N(2)-C(21)	1.444(2)	C(371)-H(37A)	0.9800
N(2)-C(27)	1.510(2)	C(371)-H(37B)	0.9800
C(21)-C(26)	1.390(3)	C(371)-H(37C)	0.9800
C(21)-C(22)	1.401(3)	C(372)-H(37D)	0.9800
C(22)-C(23)	1.395(3)	C(372)-H(37E)	0.9800
C(22)-H(22)	0.9500	C(372)-H(37F)	0.9800
C(23)-C(24)	1.386(3)	C(373)-H(37G)	0.9800
C(23)-C(231)	1.512(3)	C(373)-H(37H)	0.9800
C(231)-H(23A)	0.9800	C(373)-H(37I)	0.9800
C(231)-H(23B)	0.9800	C(31A)-C(36A)	1.395(6)
C(231)-H(23C)	0.9800	C(31A)-C(32A)	1.396(6)
C(24)-C(25)	1.388(3)	C(32A)-C(33A)	1.407(6)
C(24)-H(24)	0.9500	C(32A)-H(32A)	0.9500
C(25)-C(26)	1.396(3)	C(33A)-C(34A)	1.385(7)
C(25)-C(251)	1.515(3)	C(33A)-C(31C)	1.511(7)
C(251)-H(25A)	0.9800	C(31C)-H(31A)	0.9800
C(251)-H(25B)	0.9800	C(31C)-H(31B)	0.9800
C(251)-H(25C)	0.9800	C(31C)-H(31C)	0.9800
C(26)-H(26)	0.9500	C(34A)-C(35A)	1.384(7)

C(34A)-H(34A)	0.9500	C(67)-F(8)	1.339(2)
C(35A)-C(36A)	1.398(6)	C(67)-F(7)	1.345(3)
C(35A)-C(51C)	1.517(7)	C(68)-F(10A)	1.245(6)
C(51C)-H(51A)	0.9800	C(68)-F(11A)	1.277(8)
C(51C)-H(51B)	0.9800	C(68)-F(11)	1.298(5)
C(51C)-H(51C)	0.9800	C(68)-F(10)	1.325(5)
C(36A)-H(36A)	0.9500	C(68)-F(12A)	1.359(7)
N(4)-C(41)	1.145(2)	C(68)-F(12)	1.374(4)
C(41)-C(42)	1.432(2)	C(71)-C(76)	1.399(2)
C(42)-C(43)	1.396(2)	C(71)-C(72)	1.407(2)
C(42)-C(47)	1.402(3)	C(72)-C(73)	1.387(2)
C(43)-C(44)	1.384(3)	C(72)-H(72)	0.9500
C(43)-H(43)	0.9500	C(73)-C(74)	1.389(2)
C(44)-C(45)	1.385(3)	C(73)-C(77)	1.498(2)
C(44)-H(44)	0.9500	C(74)-C(75)	1.384(2)
C(45)-C(46)	1.386(3)	C(74)-H(74)	0.9500
C(45)-H(45)	0.9500	C(75)-C(76)	1.395(2)
C(46)-C(47)	1.380(3)	C(75)-C(78)	1.494(3)
C(46)-H(46)	0.9500	C(76)-H(76)	0.9500
C(47)-H(47)	0.9500	C(77)-F(14)	1.332(2)
B(1)-C(61)	1.634(2)	C(77)-F(15)	1.335(2)
B(1)-C(51)	1.636(2)	C(77)-F(13)	1.339(2)
B(1)-C(71)	1.638(2)	C(78)-F(17A)	1.297(7)
B(1)-C(81)	1.638(2)	C(78)-F(16)	1.306(7)
C(51)-C(52)	1.398(2)	C(78)-F(17)	1.323(6)
C(51)-C(56)	1.401(2)	C(78)-F(16A)	1.326(8)
C(52)-C(53)	1.390(2)	C(78)-F(18)	1.326(7)
C(52)-H(52)	0.9500	C(78)-F(18A)	1.330(7)
C(53)-C(54)	1.385(2)	C(81)-C(82)	1.400(2)
C(53)-C(57)	1.493(2)	C(81)-C(86)	1.404(2)
C(54)-C(55)	1.386(2)	C(82)-C(83)	1.393(2)
C(54)-H(54)	0.9500	C(82)-H(82)	0.9500
C(55)-C(56)	1.394(2)	C(83)-C(84)	1.382(3)
C(55)-C(58)	1.494(2)	C(83)-C(87)	1.501(3)
C(56)-H(56)	0.9500	C(84)-C(85)	1.388(3)
C(57)-F(1)	1.332(2)	C(84)-H(84)	0.9500
C(57)-F(3)	1.336(2)	C(85)-C(86)	1.382(2)
C(57)-F(2)	1.346(2)	C(85)-C(88)	1.492(3)
C(58)-F(4A)	1.302(10)	C(86)-H(86)	0.9500
C(58)-F(5A)	1.304(9)	C(87)-F(21)	1.332(2)
C(58)-F(5)	1.328(3)	C(87)-F(20)	1.335(2)
C(58)-F(4)	1.328(3)	C(87)-F(19)	1.339(2)
C(58)-F(6A)	1.339(10)	C(88)-F(23)	1.224(4)
C(58)-F(6)	1.342(3)	C(88)-F(22A)	1.273(4)
C(61)-C(66)	1.397(2)	C(88)-F(23A)	1.284(8)
C(61)-C(62)	1.404(2)	C(88)-F(22)	1.305(7)
C(62)-C(63)	1.387(2)	C(88)-F(24)	1.454(4)
C(62)-H(62)	0.9500	C(88)-F(24A)	1.477(5)
C(63)-C(64)	1.388(3)	C(2S)-C(1S)	1.500(3)
C(63)-C(67)	1.489(3)	C(2S)-H(2S1)	0.9800
C(64)-C(65)	1.386(3)	C(2S)-H(2S2)	0.9800
C(64)-H(64)	0.9500	C(2S)-H(2S3)	0.9800
C(65)-C(66)	1.392(2)	C(1S)-O(1S)	1.422(3)
C(65)-C(68)	1.495(3)	C(1S)-H(1S1)	0.9900
C(66)-H(66)	0.9500	C(1S)-H(1S2)	0.9900
C(67)-F(9)	1.337(2)	O(1S)-C(3S)	1.424(3)

C(3S)-C(4S)	1.489(4)
C(3S)-H(3S1)	0.9900
C(3S)-H(3S2)	0.9900
C(4S)-H(4S1)	0.9800
C(4S)-H(4S2)	0.9800
C(4S)-H(4S3)	0.9800
N(1)-V(1)-N(2)	105.36(6)
N(1)-V(1)-N(3)	123.12(6)
N(2)-V(1)-N(3)	113.67(6)
N(1)-V(1)-N(4)	96.33(6)
N(2)-V(1)-N(4)	98.44(6)
N(3)-V(1)-N(4)	116.05(6)
C(11)-N(1)-C(17)	113.32(13)
C(11)-N(1)-V(1)	121.84(11)
C(17)-N(1)-V(1)	123.14(10)
C(16)-C(11)-C(12)	119.82(15)
C(16)-C(11)-N(1)	120.60(15)
C(12)-C(11)-N(1)	119.57(15)
C(13)-C(12)-C(11)	120.77(16)
C(13)-C(12)-H(12)	119.6
C(11)-C(12)-H(12)	119.6
C(12)-C(13)-C(14)	118.46(16)
C(12)-C(13)-C(131)	120.45(17)
C(14)-C(13)-C(131)	121.08(17)
C(13)-C(131)-H(13A)	109.5
C(13)-C(131)-H(13B)	109.5
H(13A)-C(131)-H(13B)	109.5
C(13)-C(131)-H(13C)	109.5
H(13A)-C(131)-H(13C)	109.5
H(13B)-C(131)-H(13C)	109.5
C(15)-C(14)-C(13)	121.89(17)
C(15)-C(14)-H(14)	119.1
C(13)-C(14)-H(14)	119.1
C(14)-C(15)-C(16)	118.65(16)
C(14)-C(15)-C(151)	121.13(17)
C(16)-C(15)-C(151)	120.22(17)
C(15)-C(151)-H(15A)	109.5
C(15)-C(151)-H(15B)	109.5
H(15A)-C(151)-H(15B)	109.5
C(15)-C(151)-H(15C)	109.5
H(15A)-C(151)-H(15C)	109.5
H(15B)-C(151)-H(15C)	109.5
C(11)-C(16)-C(15)	120.41(16)
C(11)-C(16)-H(16)	119.8
C(15)-C(16)-H(16)	119.8
N(1)-C(17)-C(172)	110.54(14)
N(1)-C(17)-C(173)	109.26(14)
C(172)-C(17)-C(173)	109.58(15)
N(1)-C(17)-C(171)	109.56(14)
C(172)-C(17)-C(171)	110.16(15)
C(173)-C(17)-C(171)	107.69(14)
C(17)-C(171)-H(17A)	109.5
C(17)-C(171)-H(17B)	109.5
H(17A)-C(171)-H(17B)	109.5
C(17)-C(171)-H(17C)	109.5
H(17A)-C(171)-H(17C)	109.5
H(17B)-C(171)-H(17C)	109.5
C(17)-C(172)-H(17D)	109.5
C(17)-C(172)-H(17E)	109.5
H(17D)-C(172)-H(17E)	109.5
C(17)-C(172)-H(17F)	109.5
H(17D)-C(172)-H(17F)	109.5
H(17E)-C(172)-H(17F)	109.5
C(17)-C(173)-H(17G)	109.5
C(17)-C(173)-H(17H)	109.5
H(17G)-C(173)-H(17H)	109.5
C(17)-C(173)-H(17I)	109.5
H(17G)-C(173)-H(17I)	109.5
H(17H)-C(173)-H(17I)	109.5
C(21)-N(2)-C(27)	114.32(13)
C(21)-N(2)-V(1)	117.66(11)
C(27)-N(2)-V(1)	127.54(11)
C(26)-C(21)-C(22)	118.69(17)
C(26)-C(21)-N(2)	120.66(16)
C(22)-C(21)-N(2)	120.64(17)
C(23)-C(22)-C(21)	120.7(2)
C(23)-C(22)-H(22)	119.7
C(21)-C(22)-H(22)	119.7
C(24)-C(23)-C(22)	119.00(19)
C(24)-C(23)-C(231)	121.21(19)
C(22)-C(23)-C(231)	119.8(2)
C(23)-C(231)-H(23A)	109.5
C(23)-C(231)-H(23B)	109.5
H(23A)-C(231)-H(23B)	109.5
C(23)-C(231)-H(23C)	109.5
H(23A)-C(231)-H(23C)	109.5
H(23B)-C(231)-H(23C)	109.5
C(23)-C(24)-C(25)	121.56(18)
C(23)-C(24)-H(24)	119.2
C(25)-C(24)-H(24)	119.2
C(24)-C(25)-C(26)	118.5(2)
C(24)-C(25)-C(251)	121.52(19)
C(26)-C(25)-C(251)	120.0(2)
C(25)-C(251)-H(25A)	109.5
C(25)-C(251)-H(25B)	109.5
H(25A)-C(251)-H(25B)	109.5
C(25)-C(251)-H(25C)	109.5
H(25A)-C(251)-H(25C)	109.5
H(25B)-C(251)-H(25C)	109.5
C(21)-C(26)-C(25)	121.42(19)
C(21)-C(26)-H(26)	119.3
C(25)-C(26)-H(26)	119.3
N(2)-C(27)-C(272)	107.77(14)
N(2)-C(27)-C(273)	110.05(15)
C(272)-C(27)-C(273)	109.46(15)
N(2)-C(27)-C(271)	111.44(14)
C(272)-C(27)-C(271)	108.48(16)
C(273)-C(27)-C(271)	109.60(15)
C(27)-C(271)-H(27A)	109.5
C(27)-C(271)-H(27B)	109.5
H(27A)-C(271)-H(27B)	109.5

C(27)-C(271)-H(27C)	109.5
H(27A)-C(271)-H(27C)	109.5
H(27B)-C(271)-H(27C)	109.5
C(27)-C(272)-H(27D)	109.5
C(27)-C(272)-H(27E)	109.5
H(27D)-C(272)-H(27E)	109.5
C(27)-C(272)-H(27F)	109.5
H(27D)-C(272)-H(27F)	109.5
H(27E)-C(272)-H(27F)	109.5
C(27)-C(273)-H(27G)	109.5
C(27)-C(273)-H(27H)	109.5
H(27G)-C(273)-H(27H)	109.5
C(27)-C(273)-H(27I)	109.5
H(27G)-C(273)-H(27I)	109.5
H(27H)-C(273)-H(27I)	109.5
C(31)-N(3)-C(37)	116.0(4)
C(31A)-N(3)-C(37)	114.5(6)
C(31)-N(3)-V(1)	102.1(4)
C(31A)-N(3)-V(1)	103.4(6)
C(37)-N(3)-V(1)	141.81(12)
C(32)-C(31)-C(36)	119.1(3)
C(32)-C(31)-N(3)	120.8(3)
C(36)-C(31)-N(3)	120.2(4)
C(31)-C(32)-C(33)	121.0(3)
C(31)-C(32)-H(32)	119.5
C(33)-C(32)-H(32)	119.5
C(34)-C(33)-C(32)	118.2(3)
C(34)-C(33)-C(331)	121.2(3)
C(32)-C(33)-C(331)	120.6(3)
C(33)-C(331)-H(33A)	109.5
C(33)-C(331)-H(33B)	109.5
H(33A)-C(331)-H(33B)	109.5
C(33)-C(331)-H(33C)	109.5
H(33A)-C(331)-H(33C)	109.5
H(33B)-C(331)-H(33C)	109.5
C(35)-C(34)-C(33)	122.1(3)
C(35)-C(34)-H(34)	118.9
C(33)-C(34)-H(34)	118.9
C(34)-C(35)-C(36)	118.8(3)
C(34)-C(35)-C(351)	121.9(4)
C(36)-C(35)-C(351)	119.3(4)
C(35)-C(351)-H(35A)	109.5
C(35)-C(351)-H(35B)	109.5
H(35A)-C(351)-H(35B)	109.5
C(35)-C(351)-H(35C)	109.5
H(35A)-C(351)-H(35C)	109.5
H(35B)-C(351)-H(35C)	109.5
C(35)-C(36)-C(31)	120.8(4)
C(35)-C(36)-H(36)	119.6
C(31)-C(36)-H(36)	119.6
N(3)-C(37)-C(372)	108.98(14)
N(3)-C(37)-C(371)	108.92(14)
C(372)-C(37)-C(371)	109.19(16)
N(3)-C(37)-C(373)	112.38(15)
C(372)-C(37)-C(373)	108.85(16)
C(371)-C(37)-C(373)	108.47(16)
C(37)-C(371)-H(37A)	109.5
C(37)-C(371)-H(37B)	109.5
H(37A)-C(371)-H(37B)	109.5
C(37)-C(371)-H(37C)	109.5
H(37A)-C(371)-H(37C)	109.5
H(37B)-C(371)-H(37C)	109.5
C(37)-C(372)-H(37D)	109.5
C(37)-C(372)-H(37E)	109.5
H(37D)-C(372)-H(37E)	109.5
C(37)-C(372)-H(37F)	109.5
H(37D)-C(372)-H(37F)	109.5
H(37E)-C(372)-H(37F)	109.5
C(37)-C(373)-H(37G)	109.5
C(37)-C(373)-H(37H)	109.5
H(37G)-C(373)-H(37H)	109.5
C(37)-C(373)-H(37I)	109.5
H(37G)-C(373)-H(37I)	109.5
H(37H)-C(373)-H(37I)	109.5
C(36A)-C(31A)-C(32A)	120.0(6)
C(36A)-C(31A)-N(3)	123.2(6)
C(32A)-C(31A)-N(3)	116.2(6)
C(31A)-C(32A)-C(33A)	121.0(6)
C(31A)-C(32A)-H(32A)	119.5
C(33A)-C(32A)-H(32A)	119.5
C(34A)-C(33A)-C(32A)	117.2(6)
C(34A)-C(33A)-C(31C)	123.8(6)
C(32A)-C(33A)-C(31C)	119.0(6)
C(33A)-C(31C)-H(31A)	109.5
C(33A)-C(31C)-H(31B)	109.5
H(31A)-C(31C)-H(31B)	109.5
C(33A)-C(31C)-H(31C)	109.5
H(31A)-C(31C)-H(31C)	109.5
H(31B)-C(31C)-H(31C)	109.5
C(35A)-C(34A)-C(33A)	123.0(6)
C(35A)-C(34A)-H(34A)	118.5
C(33A)-C(34A)-H(34A)	118.5
C(34A)-C(35A)-C(36A)	119.1(6)
C(34A)-C(35A)-C(51C)	121.8(6)
C(36A)-C(35A)-C(51C)	118.9(6)
C(35A)-C(51C)-H(51A)	109.5
C(35A)-C(51C)-H(51B)	109.5
H(51A)-C(51C)-H(51B)	109.5
C(35A)-C(51C)-H(51C)	109.5
H(51A)-C(51C)-H(51C)	109.5
H(51B)-C(51C)-H(51C)	109.5
C(31A)-C(36A)-C(35A)	119.5(6)
C(31A)-C(36A)-H(36A)	120.2
C(35A)-C(36A)-H(36A)	120.2
C(41)-N(4)-V(1)	172.84(15)
N(4)-C(41)-C(42)	178.67(19)
C(43)-C(42)-C(47)	121.32(16)
C(43)-C(42)-C(41)	120.29(16)
C(47)-C(42)-C(41)	118.40(16)
C(44)-C(43)-C(42)	118.51(18)
C(44)-C(43)-H(43)	120.7
C(42)-C(43)-H(43)	120.7

C(43)-C(44)-C(45)	120.58(18)
C(43)-C(44)-H(44)	119.7
C(45)-C(44)-H(44)	119.7
C(44)-C(45)-C(46)	120.45(17)
C(44)-C(45)-H(45)	119.8
C(46)-C(45)-H(45)	119.8
C(47)-C(46)-C(45)	120.37(18)
C(47)-C(46)-H(46)	119.8
C(45)-C(46)-H(46)	119.8
C(46)-C(47)-C(42)	118.78(17)
C(46)-C(47)-H(47)	120.6
C(42)-C(47)-H(47)	120.6
C(61)-B(1)-C(51)	104.23(13)
C(61)-B(1)-C(71)	112.98(13)
C(51)-B(1)-C(71)	112.03(14)
C(61)-B(1)-C(81)	111.28(14)
C(51)-B(1)-C(81)	113.25(13)
C(71)-B(1)-C(81)	103.35(13)
C(52)-C(51)-C(56)	115.48(15)
C(52)-C(51)-B(1)	121.20(14)
C(56)-C(51)-B(1)	122.97(15)
C(53)-C(52)-C(51)	122.60(15)
C(53)-C(52)-H(52)	118.7
C(51)-C(52)-H(52)	118.7
C(54)-C(53)-C(52)	120.84(16)
C(54)-C(53)-C(57)	120.34(15)
C(52)-C(53)-C(57)	118.79(15)
C(53)-C(54)-C(55)	117.95(15)
C(53)-C(54)-H(54)	121.0
C(55)-C(54)-H(54)	121.0
C(54)-C(55)-C(56)	120.88(16)
C(54)-C(55)-C(58)	120.13(15)
C(56)-C(55)-C(58)	118.98(16)
C(55)-C(56)-C(51)	122.25(16)
C(55)-C(56)-H(56)	118.9
C(51)-C(56)-H(56)	118.9
F(1)-C(57)-F(3)	106.83(15)
F(1)-C(57)-F(2)	105.91(15)
F(3)-C(57)-F(2)	104.83(15)
F(1)-C(57)-C(53)	113.81(15)
F(3)-C(57)-C(53)	112.19(15)
F(2)-C(57)-C(53)	112.61(15)
F(4A)-C(58)-F(5A)	106.3(10)
F(5)-C(58)-F(4)	106.4(2)
F(4A)-C(58)-F(6A)	105.1(10)
F(5A)-C(58)-F(6A)	103.0(9)
F(5)-C(58)-F(6)	106.0(2)
F(4)-C(58)-F(6)	105.1(2)
F(4A)-C(58)-C(55)	112.5(8)
F(5A)-C(58)-C(55)	117.2(6)
F(5)-C(58)-C(55)	113.85(19)
F(4)-C(58)-C(55)	112.65(17)
F(6A)-C(58)-C(55)	111.7(7)
F(6)-C(58)-C(55)	112.14(18)
C(66)-C(61)-C(62)	115.47(15)
C(66)-C(61)-B(1)	123.98(15)
C(62)-C(61)-B(1)	120.26(15)
C(63)-C(62)-C(61)	122.32(17)
C(63)-C(62)-H(62)	118.8
C(61)-C(62)-H(62)	118.8
C(62)-C(63)-C(64)	120.92(17)
C(62)-C(63)-C(67)	117.84(18)
C(64)-C(63)-C(67)	121.23(17)
C(65)-C(64)-C(63)	117.98(16)
C(65)-C(64)-H(64)	121.0
C(63)-C(64)-H(64)	121.0
C(64)-C(65)-C(66)	120.70(17)
C(64)-C(65)-C(68)	120.44(17)
C(66)-C(65)-C(68)	118.83(18)
C(65)-C(66)-C(61)	122.57(17)
C(65)-C(66)-H(66)	118.7
C(61)-C(66)-H(66)	118.7
F(9)-C(67)-F(8)	106.30(17)
F(9)-C(67)-F(7)	106.02(17)
F(8)-C(67)-F(7)	105.91(19)
F(9)-C(67)-C(63)	113.43(19)
F(8)-C(67)-C(63)	112.78(16)
F(7)-C(67)-C(63)	111.84(17)
F(10A)-C(68)-F(11A)	114.2(7)
F(11)-C(68)-F(10)	107.5(4)
F(10A)-C(68)-F(12A)	105.7(6)
F(11A)-C(68)-F(12A)	104.9(6)
F(11)-C(68)-F(12)	104.8(4)
F(10)-C(68)-F(12)	101.3(4)
F(10A)-C(68)-C(65)	111.2(4)
F(11A)-C(68)-C(65)	111.8(5)
F(11)-C(68)-C(65)	116.0(3)
F(10)-C(68)-C(65)	112.7(4)
F(12A)-C(68)-C(65)	108.4(5)
F(12)-C(68)-C(65)	113.2(2)
C(76)-C(71)-C(72)	115.81(15)
C(76)-C(71)-B(1)	122.25(14)
C(72)-C(71)-B(1)	121.52(14)
C(73)-C(72)-C(71)	122.05(15)
C(73)-C(72)-H(72)	119.0
C(71)-C(72)-H(72)	119.0
C(72)-C(73)-C(74)	121.01(15)
C(72)-C(73)-C(77)	120.67(16)
C(74)-C(73)-C(77)	118.29(15)
C(75)-C(74)-C(73)	118.15(16)
C(75)-C(74)-H(74)	120.9
C(73)-C(74)-H(74)	120.9
C(74)-C(75)-C(76)	120.77(16)
C(74)-C(75)-C(78)	120.34(16)
C(76)-C(75)-C(78)	118.89(16)
C(75)-C(76)-C(71)	122.22(16)
C(75)-C(76)-H(76)	118.9
C(71)-C(76)-H(76)	118.9
F(14)-C(77)-F(15)	106.97(16)
F(14)-C(77)-F(13)	105.71(15)
F(15)-C(77)-F(13)	105.98(16)
F(14)-C(77)-C(73)	112.73(15)

F(15)-C(77)-C(73)	113.06(15)
F(13)-C(77)-C(73)	111.85(15)
F(16)-C(78)-F(17)	104.5(6)
F(17A)-C(78)-F(16A)	109.1(7)
F(16)-C(78)-F(18)	106.8(6)
F(17)-C(78)-F(18)	107.8(6)
F(17A)-C(78)-F(18A)	106.1(7)
F(16A)-C(78)-F(18A)	100.4(6)
F(17A)-C(78)-C(75)	115.1(6)
F(16)-C(78)-C(75)	112.7(4)
F(17)-C(78)-C(75)	112.1(5)
F(16A)-C(78)-C(75)	112.9(6)
F(18)-C(78)-C(75)	112.4(4)
F(18A)-C(78)-C(75)	112.1(4)
C(82)-C(81)-C(86)	115.45(15)
C(82)-C(81)-B(1)	122.08(14)
C(86)-C(81)-B(1)	122.21(15)
C(83)-C(82)-C(81)	122.45(16)
C(83)-C(82)-H(82)	118.8
C(81)-C(82)-H(82)	118.8
C(84)-C(83)-C(82)	120.68(17)
C(84)-C(83)-C(87)	120.73(17)
C(82)-C(83)-C(87)	118.59(17)
C(83)-C(84)-C(85)	117.99(17)
C(83)-C(84)-H(84)	121.0
C(85)-C(84)-H(84)	121.0
C(86)-C(85)-C(84)	121.22(17)
C(86)-C(85)-C(88)	119.74(18)
C(84)-C(85)-C(88)	119.04(17)
C(85)-C(86)-C(81)	122.18(16)
C(85)-C(86)-H(86)	118.9
C(81)-C(86)-H(86)	118.9
F(21)-C(87)-F(20)	107.03(17)
F(21)-C(87)-F(19)	105.80(18)
F(20)-C(87)-F(19)	105.92(17)
F(21)-C(87)-C(83)	112.57(17)
F(20)-C(87)-C(83)	112.93(17)
F(19)-C(87)-C(83)	112.06(16)
F(22A)-C(88)-F(23A)	116.4(7)

F(23)-C(88)-F(22)	111.7(7)
F(23)-C(88)-F(24)	104.0(3)
F(22)-C(88)-F(24)	99.3(6)
F(22A)-C(88)-F(24A)	97.7(4)
F(23A)-C(88)-F(24A)	98.8(7)
F(23)-C(88)-C(85)	117.7(3)
F(22A)-C(88)-C(85)	116.9(2)
F(23A)-C(88)-C(85)	115.4(7)
F(22)-C(88)-C(85)	114.2(6)
F(24)-C(88)-C(85)	107.7(2)
F(24A)-C(88)-C(85)	107.6(2)
C(1S)-C(2S)-H(2S1)	109.5
C(1S)-C(2S)-H(2S2)	109.5
H(2S1)-C(2S)-H(2S2)	109.5
C(1S)-C(2S)-H(2S3)	109.5
H(2S1)-C(2S)-H(2S3)	109.5
H(2S2)-C(2S)-H(2S3)	109.5
O(1S)-C(1S)-C(2S)	108.84(19)
O(1S)-C(1S)-H(1S1)	109.9
C(2S)-C(1S)-H(1S1)	109.9
O(1S)-C(1S)-H(1S2)	109.9
C(2S)-C(1S)-H(1S2)	109.9
H(1S1)-C(1S)-H(1S2)	108.3
C(1S)-O(1S)-C(3S)	111.94(19)
O(1S)-C(3S)-C(4S)	108.9(2)
O(1S)-C(3S)-H(3S1)	109.9
C(4S)-C(3S)-H(3S1)	109.9
O(1S)-C(3S)-H(3S2)	109.9
C(4S)-C(3S)-H(3S2)	109.9
H(3S1)-C(3S)-H(3S2)	108.3
C(3S)-C(4S)-H(4S1)	109.5
C(3S)-C(4S)-H(4S2)	109.5
H(4S1)-C(4S)-H(4S2)	109.5
C(3S)-C(4S)-H(4S3)	109.5
H(4S1)-C(4S)-H(4S3)	109.5
H(4S2)-C(4S)-H(4S3)	109.5

Computational Details. Unless stated otherwise, DFT calculations were carried out using the Gaussian 09[12] or Gaussian 16[13] suite of programs. Geometry optimizations were performed without any symmetry restrictions using the PBE0 functional,[14] the D3(BJ) empirical dispersion correction[15] and the Def2-SV(P)[16] basis set. Moreover, some geometry optimizations were carried out using the Def2-SVP[16] larger basis set for some selected species and significantly the same results were obtained. In any case, all stationary points were optimized in the gas phase by computing analytical energy gradients. The obtained stationary points were characterized by performing energy second derivatives, confirming them as minima or transition states by the number of negative eigenvalues of the Hessian matrix of the energy. To further refine the energies, single-point calculations in toluene solution using the IEF-PCM solvation method[17] on the previously optimized gas phase structures were finally performed using the larger Def2-TZVP[16] basis set. Computed electronic energies at the PBE0-D3(BJ)-PCM/Def2-TZVP level were corrected for zero-point energy, thermal energy and entropic effects calculated at the PBE0-D3(BJ)-PCM/Def2-SV(P) level to determine ΔH^0(298 K) and ΔG^0(298 K) values. Likewise, analogous calculations were also carried out with the B3LYP[18] density functional for some selected species and similar results were obtained. Minimum energy crossing points (MECP)[19] were obtained with ORCA 4.2.[20] Figures of the optimized structures were done with Avogadro 1.2.0.[21]

Selected structural parameters for adducts of **1** and **2** optimized by DFT are collected in Tables S19 and S20. Excellent agreement is observed between the computed results and experimental structural data obtained by X-ray crystallography as shown in Table S18. Likewise, good agreement between computed and experimental ν_{CN} IR wavenumbers is generally also observed (see Table S21). Finally, thermochemical data for ligand binding to **1** and **2** computed

by DFT calculations are collected in Table S22. Due to the weak computed binding for many ligands, as well as experimental difficulties, limited comparison can be made between computed thermochemical and experimental data. In spite of that, a reasonable correlation is seen between calculated and available experimental enthalpies of nitrile binding with differences generally lower than 2.5 kcal·mol^{-1} as shown in Table S23. Moreover, the thermochemical values computed for MeCN binding to **1** (ΔH = −5.5 kcal·mol^{-1}) are in good agreement with those determined experimentally for other alkyl nitriles (ΔH = −6 ± 2 kcal·mol^{-1} for AdCN binding to **1**[4]). However, as can be seen in Table S23, larger differences between DFT-calculated and experimentally measured thermochemical values are found for nitrile binding to **2** as compared to the data for complex **1**. The computed data for ΔH reported with respect to the **A** configuration of **2** is more exothermic by 6.0 and 8.7 kcal·mol^{-1} for the DFBN-**2** and AdNC-**2** adducts respectively. These differences are probably due to solvation effects in the binding process not completely considered in the calculations. The computational results were obtained using the Polarizable Continuum Model (PCM) to simulate the solvent, but in this solvation method the dispersion interactions with the solvent are neglected. However, as pointed out in the main text, there is a stabilization of **2** by interaction with the solvent (Figure 7) and the same interaction is conceivable for the free ligand in solution. Thus, thermochemical values for the reaction in Scheme S2 including explicitly a benzene solvent molecule for both complex **2** and the free nitrile were also derived from DFT calculations to simulate better what is occurring in toluene solution in the binding of ligands to **2**. The values obtained using this procedure are collected between brackets in Table S23 and a good agreement is observed now between the thermochemical values obtained by DFT calculations and experimentally in this work based on variable temperature FTIR studies or solution calorimetric data.

Finally, Table S17 supports the reliability of the DFT-computed values for the stabilization energy due to π-stacking or C-H··· π interactions as those described in the structure of DFBN-**2** (Figure 1 in the main text) and the interaction of benzene solvent with complex **2** in the **B** configuration (Figure 7, main text). The interaction energy between benzene and different arenes were computed and the results are collected in Table S17 along with available data previously computed at high-level ab-initio calculations (CCSD(T)/CBS level).[22] The good agreement between the values obtained with DFT calculations using the D3(BJ) empirical dispersion correction[15] in the current study and those estimated at the CCSD(T)/CBS level for both T-shape and parallel displaced configurations give confidence to the estimation of London dispersion energies calculated in the present work."

Table S17. Interaction energy calculated for the benzene dimer in the T-shape (T) and parallel displaced (PD) configuration and for different benzene-arene complexes in the PD configuration. All values in kcal·mol^{-1}.[a]

	B···B (T)	B···B (PD)	B···tol[b] (PD)	B···FB (PD)	B···BN (PD)	B···DFBN (PD)
DFT-D3(BJ)	−3.03	−3.01	−3.81<br>−4.45	−3.58	−4.82	−5.76
CCSD(T)/CBS[22]	−2.84	−2.73	−4.00	−3.44	−4.35	
Difference[c]	−0.19	−0.28	0.19<br>−0.45	−0.14	−0.47	

[a] B: benzene; tol: toluene; FB: fluorobenzene; BN: benzonitrile; DFBN: 2,6-difluorobenzonitrile; [b] Two different minima were calculated; [c] Value computed in the current work – value obtained in the previous study.[22]

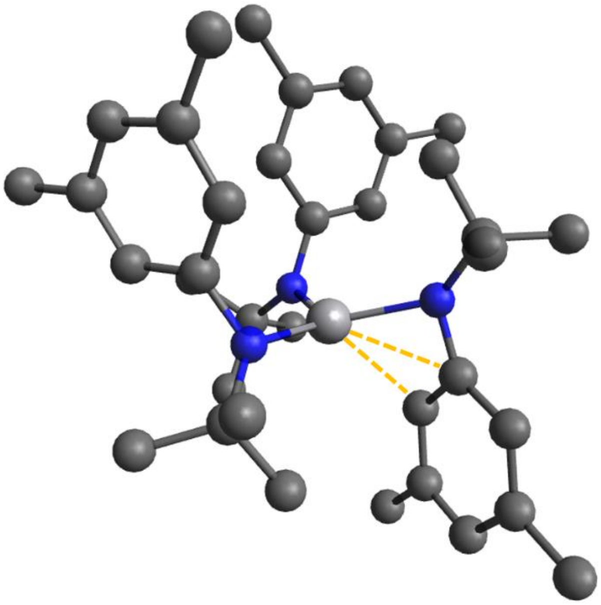

Figure S20. Optimized structure of other minimum of **2** (C) at the PBE0-D3(BJ)/Def2-SV(P) level of theory. Values related to the A configuration are ΔH = −1.8 kcal·mol^{-1}; ΔS = −10.6 cal·mol^{-1}·K^{-1}; and ΔG(25° C) = 1.3 kcal·mol^{-1}.

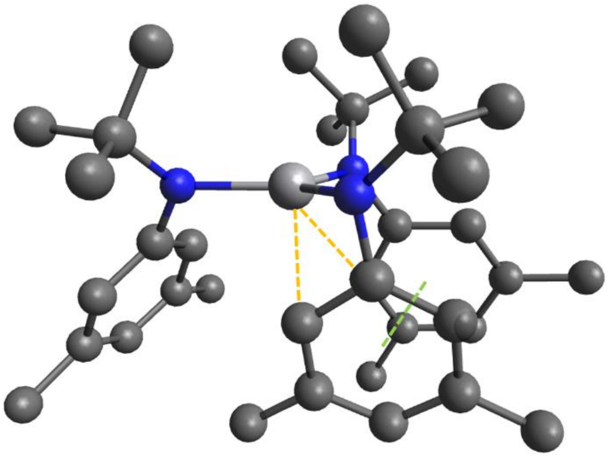

Figure S21. Optimized structure of other minimum of **2** (D) at the PBE0-D3(BJ)/Def2-SV(P) level of theory. Values related to the A configuration are ΔH = −0.4 kcal·mol^{-1}, ΔS = −15.8 cal·mol^{-1}·K^{-1}, ΔG(25° C) = 4.3 kcal·mol^{-1}.

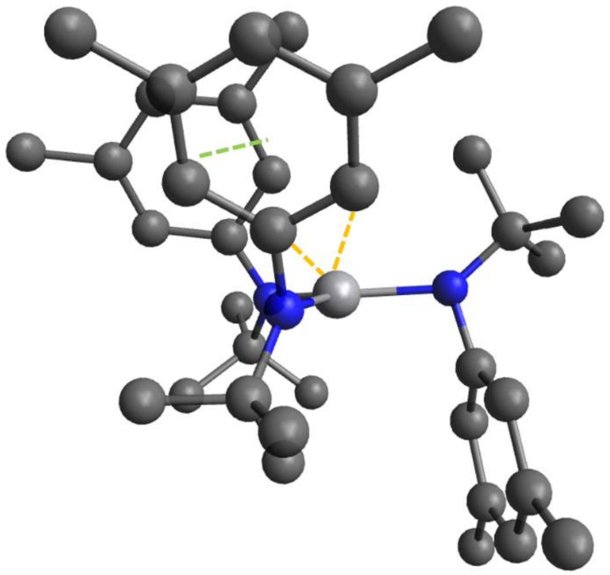

Figure S22. Optimized structure of other minimum of **2** (E) at the PBE0-D3(BJ)/Def2-SV(P) level of theory. Values related to the A configuration are ΔH = −1.3 kcal·mol^{-1}, ΔS = −11.6 cal·mol^{-1}·K^{-1}, ΔG(25º C) = 2.2 kcal·mol^{-1}.

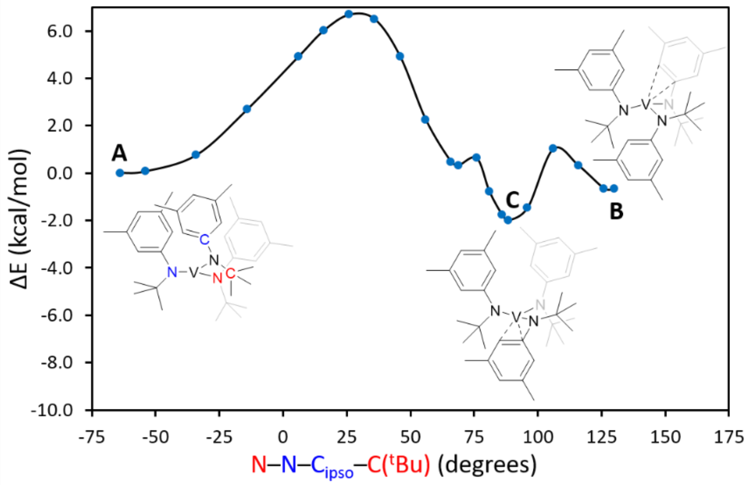

Figure S23. Energetic profile for the rotation of one of the anilide ligands in **2** for interconversion between the A and B configurations.

Table S18. Comparison between selected structural parameters for the optimized structures computed and experimental (between brackets) values.

Species	V-L (Å)	N≡C (Å)	M-N≡C (º)	N≡C-R (º)
AdNC-**2**	2.057 [2.068][a]	1.164 [1.159][a]	178.2[b] [177.0][a,b]	177.9[c]
η^1-Me_2NCN-**2**	2.045 [2.038]	1.164 [1.151]	178.2 [180.0]	179.4
DFBN-**2**	2.032 [2.042]	1.157 [1.151]	162.0 [161.8]	174.3

[a] Values taken from reference 6; [b] M-C≡N; [c] C≡N-R.

Table S19. Selected structural parameters for the adducts of **2** optimized by DFT calculations (PBE0-D3(BJ)/Def2-SV(P) level of theory).

Species	M-L (Å)	N≡C (Å)	M-N≡C (□)	N≡C-R (□)
AdNC-**2**	2.057	1.164[a]	178.2[b]	177.9
MeCN-**2**	2.060	1.153	169.6	177.4
η^1-Me_2NCN-**2**	2.050	1.162	168.7	178.7
PhCN-**2**	2.040	1.157	168.4	177.1
MesCN-**2**	2.053	1.159	161.6	175.1
DFBN-**2**	2.032	1.157	162.0	174.3
C_6F_5CN-**2**	2.024	1.157	160.5	173.5
4-$CF_3C_6H_4CN$-**2**	2.032	1.157	164.1	173.6
4-$Me_2NC_6H_4CN$-**2**	2.041	1.158	165.8	176.5

[a] M-C≡N; [b] C≡N-R.

Table S20. Selected structural parameters for the adducts of **1** optimized by DFT calculations (PBE0-D3(BJ)/Def2-SV(P) level of theory).

Species	M-L (Å)	N≡C (Å)	M-N≡C (□)	N≡C-R (□)
AdNC-**1**	1.894	1.221[a]	172.8[b]	137.7
MeCN-**1**	1.913	1.192	176.2	149.0
PhCN-**1**	1.929	1.180	173.8	172.6
MesCN-**1**	1.946	1.181	172.8	175.4
DFBN-**1**	1.916	1.183	173.0	167.6
C_6F_5CN-**1**	1.900	1.188	173.9	158.2
4-$CF_3C_6H_4CN$-**1**	1.917	1.183	173.5	167.8
4-$Me_2NC_6H_4CN$-**1**	1.945	1.178	173.5	174.0

[a] M-C≡N; [b] C≡N-R.

Table S21. Computed and experimental (between brackets) ν_{CN} IR wavenumbers for some nitrile and isonitrile adducts of **1** and **2**. All values in cm^{-1}. All computed values are multiplied by an empirical 0.928 factor chosen to match the experimental values.

L	L-**2**	L-**1**
	$\nu_{CN,calc}$ [$\nu_{CN,expt}$]	$\nu_{CN,calc}$ [$\nu_{CN,expt}$]
AdNC	2153 [2151]	1744 [1762]
MeCN	2257	1930
Me_2NCN	2244 [2256]	---
PhCN	2214 [2218]	2040 [2035]
MesCN	2191	2028
DFBN	2208 [2218]	2028 [1962]
C_6F_5CN	2204 [2207]	1986 [2000]
4-$CF_3C_6H_4CN$	2209 [2218]	2018 [2014]
4-$Me_2NC_6H_4CN$	2206 [2224]	2061 [2019]

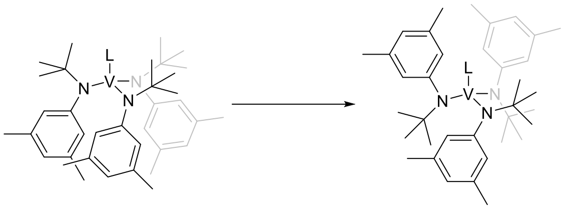

Configuration **A** Configuration **B**

L = MeCN: ΔH = -1.9; ΔG_{298K} = -3.2
L = Me_2NCN: ΔH = -2.9; ΔG_{298K} = -3.0
L = PhCN: ΔH = -3.3; ΔG_{298K} = -3.4
L = DFBN: ΔH = -2.4; ΔG_{298K} = -2.4
L = AdNC: ΔH = -3.8; ΔG_{298K} = -2.2

Scheme S1. Thermodynamic parameters computed for the isomerization reaction converting the adduct with a B configuration from the analogous structure in the A configuration at the PBE0-D3(BJ)/Def2-TZVP, IEFPCM(toluene)//PBE0-D3(BJ)/Def2-SV(P) level of theory. All values in kcal·mol^{-1}.

Table S22. Computed Thermochemical data for Ligand Binding to **1** and **2** computed at the PBE0-D3(BJ)/Def2-TZVP,IEFPCM(toluene)//PBE0-D3(BJ)/Def2-SV(P) level of theory. ΔH and ΔG values in kcal·mol^{-1} and ΔS in cal·mol^{-1}·K^{-1}.[a] Between brackets values obtained using the reaction shown in Scheme S2.

	**1**		**2**	
L	ΔH	ΔS	ΔH	ΔS
MeCN	−14.2 [−10.5]	−40.1	−5.5	−51.9
Me_2NCN	−18.3 [−13.8]	−46.1	−23.7[b]	−63.0[b]
PhCN	−17.7 [−13.4]	−47.1	−13.5	−57.1
MesCN	−19.4 [−13.8]	−57.6	−13.1	−65.1
DFBN	−16.4 [−10.5]	−52.3	−15.5	−61.7
C_6F_5CN	−16.3 [−9.8]	−55.9	−17.8	−65.2
4-$CF_3C_6H_4CN$	−17.5 [−12.3]	−52.9	−16.1	−60.7
4-$Me_2NC_6H_4CN$	−18.8 [−13.6]	−45.9	−10.6	−56.3
MeNC	−20.0 [−16.4]	−41.9	−31.4	−53.6
AdNC	−25.8 [−21.0]	−51.7	−32.4	−63.5

[a] Values reported with respect to the **A** configuration for complex **1** and **2**; [b] Me_2NCN forms an η^2 species when bound.[8]

Table S23. Comparison of experimental and computational thermochemical data for adduct formation from RCN or RNC ligand addition to **1** and **2**. Values in kcal·mol^{-1}. Between brackets values obtained using the reaction shown in Scheme S2.

	ΔH_{calc}	ΔH_{expt}	$\Delta\Delta H_{expt\text{-}calc}$
Me2NCN-**1**	−23.7	−22.0±1.0[4,a]	1.7
PhCN-**1**	−13.5	−14.5±1.5[4]	−1.0
MesCN-**1**	−13.1	−15.4±1.5[4]	−2.3
AdNC-**1**	−32.4	−29.1±0.5[5]	3.3
Me2NCN-**2**	−18.3 [−13.8]	−16.4±0.8[b,c]	1.9 [−2.6]
PhCN-**2**	−17.7 [−13.4]	−14.4±1.5[c]	3.3 [−1.0]
DFBN-**2**	−16.4 [−10.5]	−10.4±0.8[d]	6.0 [0.1]
AdNC-**2**	−25.8 [−21.0]	−17.1±0.7[8]	8.7 [3.9]

[a] η^2-binding mode; [b] η^1-binding mode; [c] Determined in this work by Calorimetry; [d] Determined in this work by variable temperature FTIR studies.

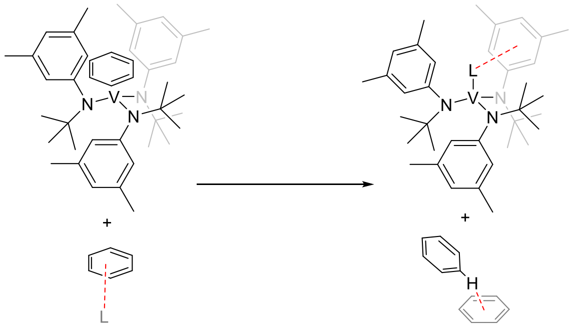

Scheme S2. Reaction of the most stable structure from Figure 7 and L containing a π-stacking or C-H··· π interactions with a benzene molecule to yield complex L-**2** and the most stable tilted T-shape benzene dimer.[22]

Table S24. Computed values of relative energy V···N≡C angle and Energy as a function of the V···$N_{nitrile}$ distance for DFBN-**2** and AdCN-**2** computed at the B3LYP-D3(BJ)/Def2-TZVP, IEFPCM(toluene)//B3LYP-D3(BJ)/Def2-SV(P) level of theory.

DFBN			AdCN		
V···$N_{nitrile}$ (Å)	V···N≡C (degrees)	Relative Energy (kcal·mol^{-1})	V···$N_{nitrile}$ (Å)	V···N≡C (degrees)	Relative Energy (kcal·mol^{-1})
2.023	162.2	0.0	2.049	179.6	0.0
2.123	160.3	0.8	2.149	175.5	0.7
2.223	158.6	2.5	2.249	175.1	2.4
2.323	156.4	4.5	2.349	174.4	4.6
2.423	153.4	6.7	2.449	173.6	6.9
2.523	150.5	8.8	2.549	172.6	9.1
2.623	147.1	10.7	2.649	171.0	11.2
2.723	150.2	12.0	2.749	171.1	13.0
2.823	135.9	13.0	2.849	169.4	14.6
2.923	131.4	13.4	2.949	162.3	16.2
3.023	128.0	13.7	3.049	147.4	17.8
3.223	127.0	14.1	3.249	118.6	17.8
3.423	122.4	14.4	3.449	113.1	17.7

Table S25. Computed values of relative energy V···N≡C angle and Energy as a function of the V···$N_{nitrile}$ distance for MeCN-**2** and MesCN-**2** computed at the B3LYP-D3(BJ)/Def2-TZVP, IEFPCM(toluene)//B3LYP-D3(BJ)/Def2-SV(P) level of theory.

MeCN			MesCN		
V···$N_{nitrile}$ (Å)	V···N≡C (degrees)	Relative Energy (kcal·mol^{-1})	V···$N_{nitrile}$ (Å)	V···N≡C (degrees)	Relative Energy (kcal·mol^{-1})
2.064	169.0	0.0	2.024	161.8	0.0
2.164	166.7	0.8	2.124	159.7	0.6
2.264	164.1	2.4	2.224	158.2	2.0
2.364	162.0	4.3	2.324	156.1	4.0
2.464	161.6	6.1	2.424	153.2	6.3
2.564	159.4	7.9	2.524	150.8	8.4
2.664	156.4	9.5	2.624	149.6	10.2
2.764	153.6	11.0	2.724	147.0	11.9
2.864	151.0	12.2	2.824	144.2	13.4
2.964	148.7	13.3	2.924	140.2	14.3
3.064	124.0	13.4	3.024	134.9	14.8
3.264	115.9	13.8	3.424	120.0	16.1
3.464	108.0	14.0			

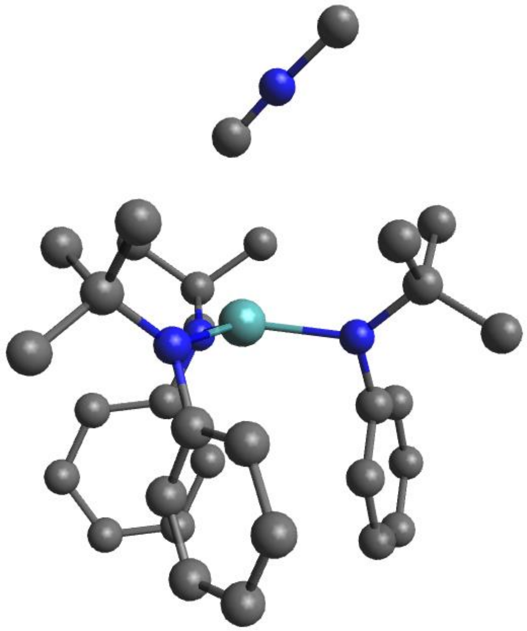

Figure S24. Optimized structure of the transition state for binding MeNC in the high spin quartet state of **1’** at the PBE0-D3(BJ)/Def2-SV(P) level of theory. Hydrogen atoms omitted for clarity. Selected structural and thermochemical parameters for this species are collected in Tables S26 and S27 respectively.

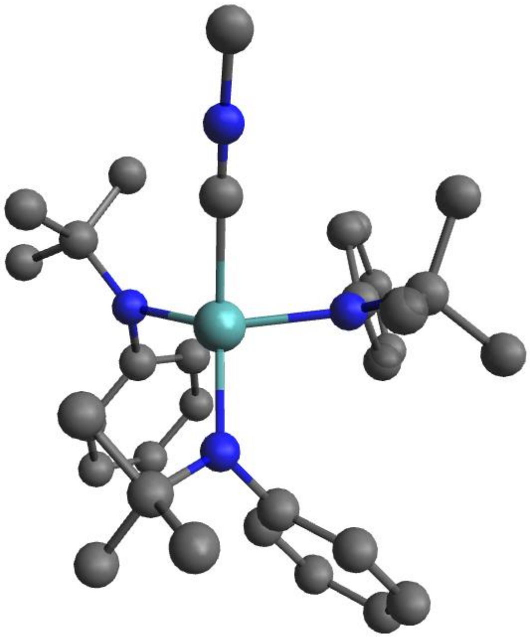

Figure S25. Optimized structure of a minimum for binding MeNC in the high spin quartet state of **1’** at the PBE0-D3(BJ)/Def2-SV(P) level of theory. Hydrogen atoms omitted for clarity. Selected structural and thermochemical parameters for this species are collected in Tables S26 and S27 respectively.

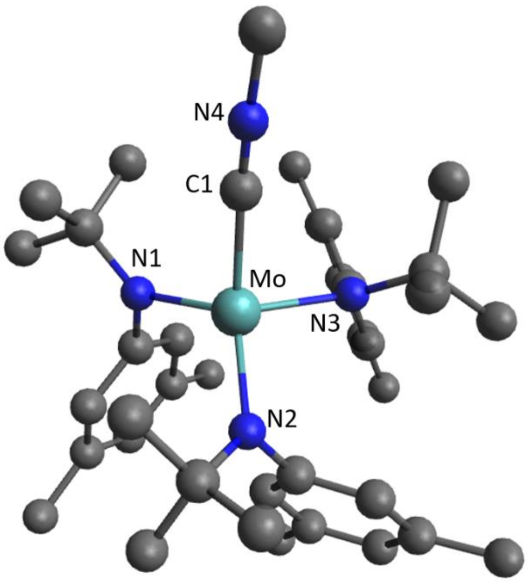

Figure S26. Optimized structure of a minimum for binding MeNC in the high spin quartet state of **1** at the PBE0-D3(BJ)/Def2-SV(P) level of theory. Hydrogen atoms omitted for clarity. Selected distances (Å) and angles (°):Mo–C1 = 2.099; Mo–N1 = 2.017; Mo–N2 = 2.018; Mo–N3 = 2.059; C1–N4 = 1.179; N2–Mo–C1 = 139.8; N1–Mo–C1 = 95.5; N3–Mo–C1 = 86.2; N2–Mo–N1 = 111.0; N2–Mo–N3 = 96.7; N1–Mo–N3 = 132.3; Mo–C1–N4 = 168.2.

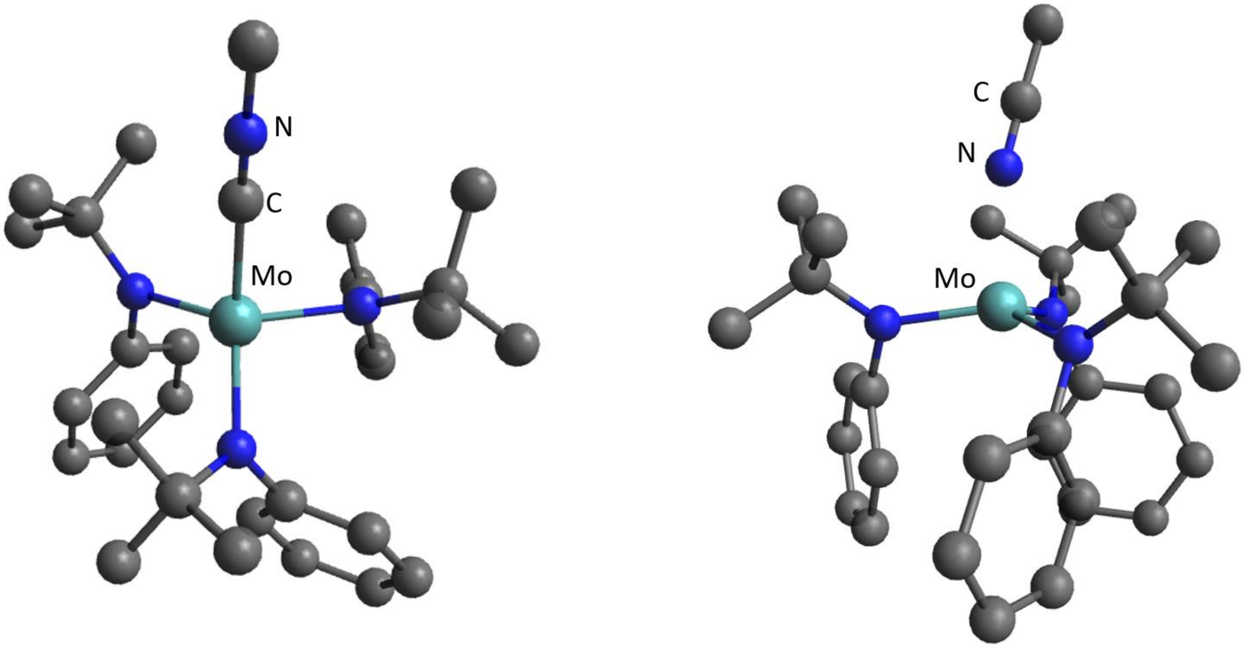

Figure S27. Computed structures of MECP between the quartet and doublet potential energy surfaces for MeNC (left) and for MeCN (right) binding to **1’**. Hydrogen atoms omitted for clarity. Selected structural and thermochemical parameters for the MECP in the MeNC case are collected in Tables S26 and S27 respectively.

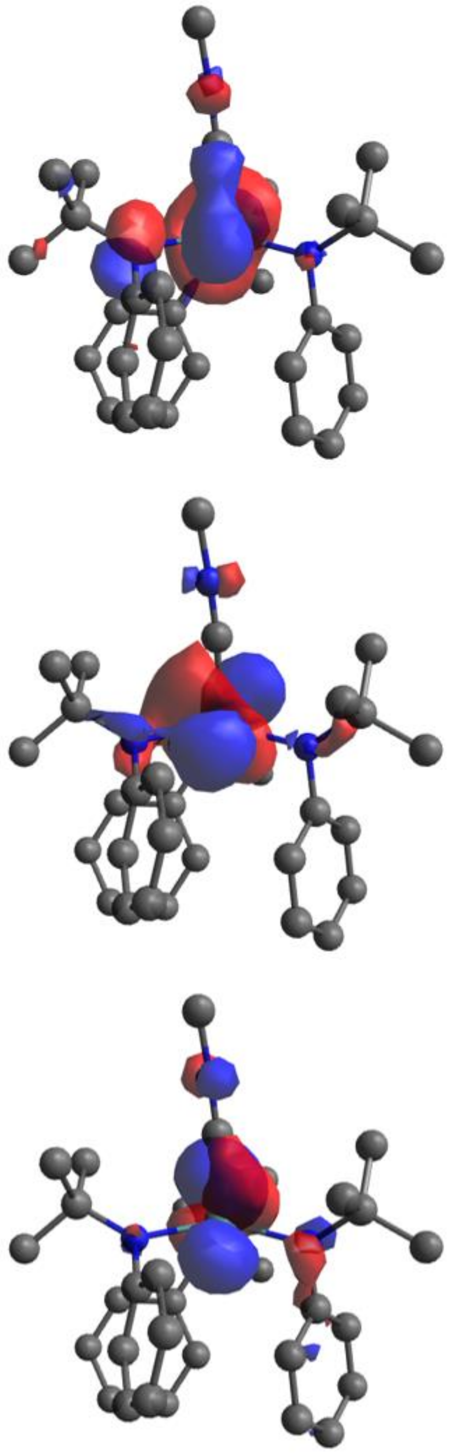

Figure S28. SOMOS of the optimized structure of the minima for MeNC binding to **1**’ in the quartet state (structure shown in Figure S25) at the PBE0-D3(BJ)/Def2-SV(P) level of theory. Hydrogen atoms omitted for clarity. Isovalue = 0.04.

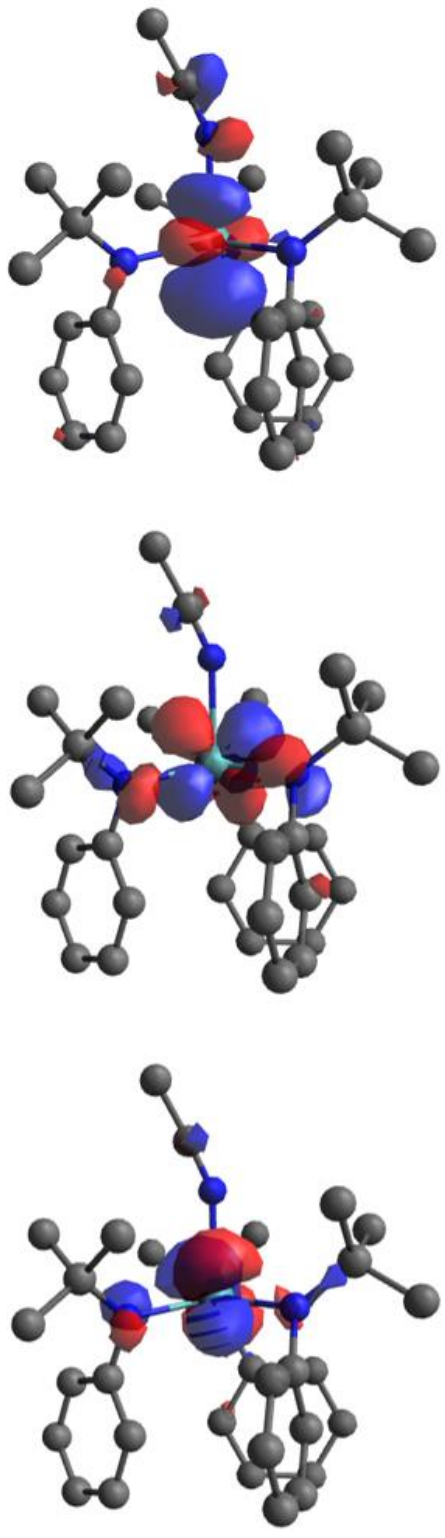

Figure S29. SOMOS of the optimized structure of the MECP between the quartet and doublet states in MeCN binding to **1**’ (structure shown in Figure S27 (right)) at the PBE0-D3(BJ)/Def2-SV(P) level of theory. Hydrogen atoms omitted for clarity. Isovalue = 0.04.

Table S26. Selected structural parameters for the species in the mechanism of the reaction: MeNC + **1**’ → MeNC-**1**’.

Compound	Spin multiplicity[a]	Mo–C (Å)	C≡N (Å)	Mo–C≡N (°)	C≡N–Me (°)
MeNC	S	---	1.172	---	179.9
TS	Q	3.166	1.172	128.5	176.7
Min	Q	2.099	1.179	168.2	177.5
MECP	Q-D	2.069	1.182	167.6	179.0
MeNC-**1**’	D	1.887	1.223	173.7	134.5

[a] S = singlet, D = doublet, Q = quartet.

Table S27. Selected thermodynamic parameters for the species in the mechanism of the reaction: MeNC + **1**’ → MeNC-**1**’. Energy, enthalpy and Gibbs energy values in kcal·mol^{-1} and entropies in cal·K^{-1}·mol^{-1}.

	MeNC + **1**’	TS	Min	MECP	MeNC-**1**’
Δ*E*	0.0	1.1	−6.0	−5.2	−32.9
Δ*H*	0.0	1.8	−4.6	---	−31.2
Δ*S*	0.0	−37.9	−43.6	---	−47.6
Δ*G*(25°C)	0.0	13.2	8.4	---	−17.0

References

1. Fickes, M. G. Synthesis and Reactivity of Vanadium and Niobium Complexes Containing Sterically Demanding Amido Ligands, Ph.D Thesis, Massachusetts Institute of Technology, 1998.

2. Nafady, A.; Chin, T. T.; Geiger, W. E. Manipulating the Electrolyte Medium to Favor Either One-Electron or Two-Electron Oxidation Pathways for (Fulvalendiyl)dirhodium Complexes. *Organometallics* **2006**, *25*, 1654-1663.

3. Fulmer, G. R.; Miller, A. J. M.; Sherden, N. H.; Gottlieb, H. E.; Nudelman, A.; Stoltz, B. M.; Bercaw, J. E.; Goldberg, K. I. NMR Chemical Shifts of Trace Impurities: Common Laboratory Solvents, Organics, and Gases in Deuterated Solvents Relevant to the Organometallic Chemist. *Organometallics* **2010**, *29*, 2176-2179.

4. Germain, M. E.; Temprado, M.; Castonguay, A.; Kryatova, O. P.; Rybak-Akimova, E. V.; Curley, J. J.; Mendiratta, A.; Tsai, Y.-C.; Cummins, C. C.; Prabhakar, R.; McDonough, J. E.; Hoff, C. D. Coordination-Mode Control of Bound Nitrile Radical Complex Reactivity: Intercepting End-on Nitrile−Mo(III) Radicals at Low Temperature, *J. Am. Chem. Soc.* **2009**, *131*, 15412–15423.

5. Stephens, F. H.; Figueroa, J. S.; Cummins, C. C.; Kryatova, O.; Kryatov, S. V.; Rybak-Akimova, E. V.; McDonough, J. E.; Hoff, C. D.; Small Molecule Activation by Metallaziridine Hydride Complexes: Mechanistic Sequence of the Small Molecule-Binding and Metallaziridine Ring-Opening Steps, *Organometallics*, **2004**, *23*, 3126-3138.

6. Majumdar, S.; Stauber, J. M.; Palluccio, T. D.; Cai, X.; Velian, A.; Rybak-Akimova, E. V.; Temprado, M.; Captain, B.; Cummins, C. C.; Hoff, C. D., Role of Axial Base Coordination in Isonitrile Binding and Chalcogen Atom Transfer to Vanadium(III) Complexes. *Inorg. Chem.* **2014**, *53*, 11185-11196.

7. While data here has been reported earlier for most ligands (references 16, 18 and 24), it has been repeated and checked in this work and some data revised slightly.

8. Tsai, Y. C.; Stephens, F. H.; Meyer, K.; Mendiratta, A.; Gheorghiu, M. D.; Cummins, C. C. Reactions of Organic Nitriles with a Three-Coordinate Molybdenum(III) Complex and with a Related Molybdaziridine-Hydride. *Organometallics* **2003**, *22*, 2902-2913.

9. Sheldrick, G. M. SHELXT - Integrated space-group and crystal-structure determination. *Acta Cryst.* **2015**, *A71*, 3-8.

10. Sheldrick, G. M. Crystal structure refinement with SHELXL. *Acta Cryst.* **2015**, *C71*, 3-8.

11. Müller, P. Practical suggestions for better crystal structures. *Crystallography Reviews* **2009**, *15*, 57-83.

12. Gaussian 09, Revision D.01, Frisch, M. J.; Trucks, G. W.; Schlegel, H. B.; Scuseria, G. E.; Robb, M. A.; Cheeseman, J. R.; Scalmani, G.; Barone, V.; Mennucci, B.; Petersson, G. A.; Nakatsuji, H.; Caricato, M.; Li, X.; Hratchian, H. P.; Izmaylov, A. F.; Bloino, J.; Zheng, G.; Sonnenberg, J. L.; Hada, M.; Ehara, M.; Toyota, K.; Fukuda, R.; Hasegawa, J.; Ishida, M.; Nakajima, T.; Honda, Y.; Kitao, O.; Nakai, H.; Vreven, T.; Montgomery, J. A. Jr.; Peralta, J. E.; Ogliaro, F.; Bearpark, M.; Heyd, J. J.; Brothers, E.; Kudin, K. N.; Staroverov, V. N.; Keith, T.; Kobayashi, R.; Normand, J.; Raghavachari, K.; Rendell, A.; Burant, J. C.; Iyengar, S. S.; Tomasi, J.; Cossi, M.; Rega, N.; Millam, J. M.; Klene, M.; Knox, J. E.; Cross, J. B.; Bakken, V.; Adamo, C.; Jaramillo, J.; Gomperts, R.; Stratmann, R. E.; Yazyev, O.; Austin, A. J.; Cammi, R.; Pomelli, C.; Ochterski, J. W.; Martin, R. L.; Morokuma, K.; Zakrzewski, V. G.; Voth, G. A.; Salvador,

P.; Dannenberg, J. J.; Dapprich, S.; Daniels, A. D.; Farkas, O.; Foresman, J. B.; Ortiz, J. V.; Cioslowski, J.; Fox, D. J. Gaussian, Inc., Wallingford CT, 2013.

13. Gaussian 16, Revision B.01, Frisch, M. J.; Trucks, G. W.; Schlegel, H. B.; Scuseria, G. E.; Robb, M. A.; Cheeseman, J. R.; Scalmani, G.; Barone, V.; Petersson, G. A.; Nakatsuji, H.; Li, X.; Caricato, M.; Marenich, A. V.; Bloino, J.; Janesko, B. G.; Gomperts, R.; Mennucci, B.; Hratchian, H. P.; Ortiz, J. V.; Izmaylov, A. F.; Sonnenberg, J. L.; Williams-Young, D.; Ding, F.; Lipparini, F.; Egidi, F.; Goings, J.; Peng, B.; Petrone, A.; Henderson, T.; Ranasinghe, D.; Zakrzewski, V. G.; Gao, J.; Rega, N.; Zheng, G.; Liang, W.; Hada, M.; Ehara, M.; Toyota, K.; Fukuda, R.; Hasegawa, J.; Ishida, M.; Nakajima, T.; Honda, Y.; Kitao, O.; Nakai, H.; Vreven, T.; Throssell, K.; Montgomery, J. A., Jr.; Peralta, J. E.; Ogliaro, F.; Bearpark, M. J.; Heyd, J. J.; Brothers, E. N.; Kudin, K. N.; Staroverov, V. N.; Keith, T. A.; Kobayashi, R.; Normand, J.; Raghavachari, K.; Rendell, A. P.; Burant, J. C.; Iyengar, S. S.; Tomasi, J.; Cossi, M.; Millam, J. M.; Klene, M.; Adamo, C.; Cammi, R.; Ochterski, J. W.; Martin, R. L.; Morokuma, K.; Farkas, O.; Foresman, J. B.; Fox, D. J. Gaussian, Inc.: Wallingford, CT, 2016.

14. Adamo, C.; Barone, V. Toward reliable density functional methods without adjustable parameters: The PBE0 model. *J. Chem. Phys.* **1999**, *110*, 6158-6170.

15. a) Becke, A. D.; Johnson, E. R. A Density-Functional Model of the Dispersion Interaction. *J. Chem. Phys.* **2005**, *123*, 154101; b) Grimme, S.; Ehrlich, S.; Goerigk, L. Effect of the damping function in dispersion corrected density functional theory. *J. Comp. Chem.* **2011**, *32*, 1456-1465.

16. Weigend, F.; Ahlrichs, R. Balanced basis sets of split valence, triple zeta valence and quadruple zeta valence quality for H to Rn: Design and assessment of accuracy. *Phys. Chem. Chem. Phys.* **2005**, *7*, 3297-3305.

17. a) Miertuš, S.; Scrocco, E.; Tomasi. Electrostatic interaction of a solute with a continuum. A direct utilizaion of AB initio molecular potentials for the prevision of solvent effects. *J. Chem. Phys.* **1981**, *55*, 117-129; b) Tomasi, J.; Mennucci, B.; Cammi, R. Quantum Mechanical Continuum Solvation Models. *Chem. Rev.* **2005**, *105*, 2999-3094; c) Scalmani, G.; Frisch, M. J. Continuous surface charge polarizable continuum models of solvation. I. General formalism. *J. Chem. Phys.* **2010**, *132*, 114110.

18. (a) Becke, A. D. Density- functional thermochemistry. III. The role of exact exchange. *J. Chem. Phys.* **1993**, *98*, 5648-5652; (b) Lee, C.; Yang, W.; Parr, R. G. Development of the Colle-Salvetti correlation-energy formula into a functional of the electron density. *Phys. Rev. B*, **1988**, *37*, 785-789.

19. a) Harvey, J. N.; Aschi, M.; Schwarz, H.; Koch, W. The singlet and triplet states of phenyl cation. A hybrid approach for locating minimum energy crossing points between non-interacting potential energy surfaces. *Theor. Chem. Acc.* **1998**, *99*, 95–99; b) Smith, K. M.; Poli, R.; Harvey, J. N. Ligand dissociation accelerated by spin state change: locating the minimum energy crossing point for phosphine exchange in $CpMoCl_2(PR_3)_2$ complexes. *New. J. Chem.* **2000**, *24*, 77–80; c) Harvey, J. N.; Poli, R.; Smith, K. M. Understanding the reactivity of transition metal complexes involving multiple spin states. *Coord. Chem. Rev.* **2003**, *238*, 347-361; d) Poli, R.; Harvey, J. N. Spin forbidden chemical reactions of transition metal compounds. New ideas and new computational challenges. *Chem. Soc. Rev.* **2003**, *32*, 1–8; e) Carreón-Macedo, J. L.; Harvey, J. N. Do spin state changes matter in organometallic chemistry? A computational study. *J. Am. Chem. Soc.* **2004**, *126*, 5789–5797.

20. a) Neese, F. The ORCA program system. *WIREs Comput. Mol. Sci.* **2012**, *2*, 73–78; b) Neese, F. Software update: the ORCA program system, version 4.0. *WIREs Comput. Mol. Sci.* **2018**, *8*, e1327; c) Neese, F.; Wennmohs, F.; Becker, U.; Riplinger, C. The ORCA quantum chemistry program package. *J. Chem. Phys.* **2020**, *152*, 224108.

21. a) Avogadro: an open-source molecular builder and visualization tool. Version 1.2.0. http://avogadro.cc/; b) Hanwell, M. D.; Curtis, D. E.; Lonie, D. C.; Vandermeersch, T.; Zurek, E.;

Hutchison, G. R. Avogadro: An advanced semantic chemical editor, visualization, and analysis platform. *J. Cheminformatics*, **2012**, *4*, 17.

22. Lee, E. C.; Kim, D.; Jurečka, P.; Tarakeshwar, P.; Hobza, P.; Kim, K. S. Understanding of assembly phenomena by aromatic−aromatic interactions: benzene dimer and the substituted systems. *J. Phys. Chem. A*, **2007**, *111*, 3446–3457.
